# Practical Anemia Bundle and Hemoglobin Recovery in Critical Illness: A Randomized Clinical Trial

**DOI:** 10.1001/jamanetworkopen.2025.2353

**Published:** 2025-03-28

**Authors:** Matthew A. Warner, Matthew L. Johnson, Andrew C. Hanson, Emma Fortune, Gerald W. Flaby, Phillip J. Schulte, Valerie M. Hazelton, Ronald S. Go, W. Brian Beam, Jonathan E. Charnin, Brenda K. Anderson, Brad Karon, Andrea L. Cheville, Ognjen Gajic, Daryl J. Kor

**Affiliations:** 1 Division of Critical Care Medicine, Department of Anesthesiology and Perioperative Medicine, Mayo Clinic, Rochester, Minnesota; 2 Anesthesia Clinical Research Unit, Department of Anesthesiology and Perioperative Medicine, Mayo Clinic, Rochester, Minnesota; 3 Division of Clinical Trials and Biostatistics, Department of Quantitative Health Sciences, Mayo Clinic, Rochester, Minnesota; 4 Division of Health Care Delivery Research, Mayo Clinic, Rochester, Minnesota; 5 Robert D. and Patricia E. Kern Center for the Science of Health Care Delivery, Mayo Clinic, Rochester, Minnesota; 6 Department of Hematology, Mayo Clinic, Rochester, Minnesota; 7 Department of Laboratory Medicine and Pathology, Mayo Clinic, Rochester, Minnesota; 8 Department of Physical Medicine and Rehabilitation, Mayo Clinic, Rochester, Minnesota; 9 Division of Pulmonary and Critical Care Medicine, Department of Medicine, Mayo Clinic, Rochester, Minnesota

## Abstract

**Importance:**

Anemia is a common complication of surgery and acute illness that is associated with adverse clinical outcomes. The role of anemia prevention and treatment strategies in this setting remains unclear.

**Objective:**

To evaluate the effect of a multifaceted anemia management bundle vs standard care on posthospitalization hemoglobin recovery and multidimensional functional outcomes in survivors of acute illness.

**Design, Setting, and Participants:**

This parallel group randomized clinical trial, known as the Practical Anemia Bundle for Sustained Blood Recovery, was conducted at postsurgical and medical intensive care units at a large US medical center. Critically ill patients (aged ≥18 years) with moderate to severe anemia (hemoglobin concentration <10 g/dL) were enrolled between March 2022 and November 2023. Participants were randomly assigned 1:1 to the intervention or standard care group. Intention-to-treat analyses were performed between July 2024 and January 2025.

**Intervention:**

The intervention bundle was delivered throughout the duration of hospitalization and included optimized phlebotomy practices, clinical decision support, and pharmacological anemia treatment with intravenous iron.

**Main Outcomes and Measures:**

The primary outcome was the mean difference in hemoglobin concentration at 1 month after hospital discharge.

**Results:**

A total of 100 patients (median [IQR] age, 68 [61-72] years; 57 men [57.0%]; 65 [65.0%] with postsurgical admission to the intensive care unit) were enrolled during acute illness. Forty-nine patients (49.0%) were assigned to receive the intervention, and 51 (51.0%) were assigned to receive standard care. Hemoglobin concentration at 1 month after discharge was greater in patients receiving the intervention vs standard care (median [IQR], 12.2 [11.8-13.0] g/dL vs 11.5 [10.2-12.6] g/dL; adjusted mean difference, 0.69 [95% CI, 0.13-1.20] g/dL; *P* = .02).

**Conclusions and Relevance:**

This randomized clinical trial found that a multifaceted anemia prevention and treatment bundle was feasible, was well tolerated, and improved posthospitalization hemoglobin concentrations up to 3 months in critically ill adults. These findings can inform the design of future trials.

**Trial Registration:**

ClinicalTrials.gov Identifier: NCT05167734

## Key Points

**Question:**

Does a multifaceted anemia prevention and treatment bundle vs standard care improve hemoglobin recovery and functional outcomes in adults with postsurgical or medical critical illness?

**Findings:**

In this randomized clinical trial that included 100 critically ill adults, receipt of the anemia intervention bundle achieved significantly higher hemoglobin recovery up to 3 months after hospital discharge compared with standard care.

**Meaning:**

The findings of this trial indicate that using an anemia prevention and treatment bundle improves hemoglobin concentrations after acute illness; these data can inform the design of future trials.

## Teaser

This randomized clinical trial assesses the postoperative and postdischarge changes in hemoglobin concentration and functional outcomes among critically ill adults who received anemia prevention and treatment vs those who underwent usual care.

## Introduction

Anemia is remarkably common after surgery and during critical illness.^1,2,3^ Over the past 20 years, clinicians have become more tolerant of anemia during acute illness,^3,4^ a phenomenon stemming largely from the Transfusion Requirements in Critical Care trial,^5^ which found similar survival with restrictive and liberal red blood cell (RBC) transfusion strategies. However, survival from surgery and critical illness does not guarantee that quality of life is preserved; up to half of survivors of critical illness have functional deficits in 1 or multiple domains of physical function, cognition, mental health, or quality of life.^6,7,8,9,10,11^ Thus, it is important to identify modifiable risk factors for impaired functional recovery. Anemia is a potential factor.^12^ Anemia in those surviving major surgery or critical illness is associated with impaired physical performance, unanticipated hospital readmissions, and mortality.^3,12,13^

Anemia management strategies in acute illness may be preventive or therapeutic. Preventive strategies aim to minimize iatrogenic contributions to anemia. Examples include low-volume blood sampling and use of clinical decision support to promote appropriate laboratory use, minimize excessive blood draws, and facilitate appropriate bleeding prevention. Therapeutic nontransfusion-based anemia management options include intravenous (IV) iron and erythropoietin. While these therapies augment hemoglobin recovery, they have not been widely adopted in clinical practice given that trials found that they had inconsistent effects on transfusion use and hospital mortality.^14,15,16,17,18,19^ Beyond these short-term effects, there is limited information regarding how strategies to augment hemoglobin recovery during and after critical illness might affect posthospitalization outcomes, such as daily functioning and hospital readmissions.^12,13,20,21,22,23^

In this randomized clinical trial, we aimed to evaluate the effect of a multifaceted anemia prevention and treatment bundle vs standard care on posthospitalization hemoglobin recovery and multidimensional functional outcomes in survivors of acute illness. We hypothesized that anemia management would improve hemoglobin concentrations while also generating benefits for transfusion and multidimensional functional recovery.

## Methods

### Trial Design

This investigator-initiated, parallel group, single-center randomized clinical trial, known as the Practical Anemia Bundle for Sustained Blood Recovery, was conducted at a large US medical center and enrolled participants from March 2022 through November 2023. Its rationale and design have been reported previously,^24^ and the trial protocol is provided in Supplement 1. The trial was approved by the Mayo Clinic Institutional Review Board. Written informed consent was obtained from participants or their surrogates. A data safety monitoring plan was used, with daily capture and review of adverse events up to 3 months after hospitalization. We followed the Consolidated Standards of Reporting Trials (CONSORT) reporting guideline.^25^

### Trial Population

Eligible patients were critically ill adults (aged ≥18 years) who were admitted to surgical or medical intensive care units (ICUs) with moderate to severe anemia (hemoglobin concentration <10 g/dL within 24 hours of enrollment; to convert to g/L, multiply by 10.0) and were living within 60 miles (96.5 km) of the study institution to facilitate in-person follow-up assessments. Exclusion criteria were severe anemia preceding hospitalization (hemoglobin concentration <9 g/dL), receipt of more than 10 units of allogeneic RBCs in the preceding 48 hours, use of IV iron or erythropoiesis-stimulating agents within 30 days, inability to complete outcome assessments, pregnancy, acute thrombosis or myocardial ischemia without revascularization, stroke in the past 3 months, use of mechanical circulatory support, absence of venous thromboembolic chemoprophylaxis apart from patients with acute bleeding or recent surgery, and sepsis with fewer than 48 hours of appropriate antimicrobial therapy and/or lack of source control.

Trial coordinators reviewed ICU censuses daily to identify eligible patients, with formal adjudication by the principal investigator (M.A.W.). Patients satisfying the inclusion or exclusion criteria, or their legal proxies, were approached by trial coordinators for written informed consent. Patients with modifiable exclusions (eg, sepsis, mechanical circulatory support) were rescreened daily during their index ICU admission and approached if they met the inclusion or exclusion criteria.

### Intervention and Randomization

Participants were randomly assigned 1:1 to intervention vs standard care using a stratified permuted block design by anemia etiologic cause (iron responsive vs non–iron responsive) and by ICU admission indication (surgical vs nonsurgical) via the REDCap randomization module (Vanderbilt University).^26^ Iron responsive was defined as acute blood loss anemia secondary to major bleeding (ie, acute blood loss of >500 mL associated with hemoglobin concentration decrease of >2 g/dL or requiring surgery or procedural intervention for hemorrhage control), ferritin level of less than 100 ng/mL (to convert to μg/L, multiply by 1.0), or transferrin saturation of less than 20% on immediate prerandomization laboratory studies.

The intervention (ie, anemia prevention and treatment bundle) consisted of 3 components that were applied throughout the duration of the index hospitalization, as detailed previously^24^: (1) optimized phlebotomy practices—low-volume blood draws, bundled laboratory timings, and closed-loop blood sampling performed by a dedicated phlebotomy team independent from the clinical or study team; (2) clinical decision support—best practice alerts embedded in the electronic health record and a daily rounding checklist prompting care team members to minimize nonessential laboratory tests, provide appropriate bleeding prophylaxis, decrease unnecessary IV fluids, and promote nutrition; and (3) pharmacological anemia treatment—1000-mg IV iron dextran administered immediately after enrollment to participants with iron-responsive anemia or 40 000 units of subcutaneous erythropoietin (plus IV iron, if ferritin level was <1000 ng/mL) for participants with non–iron-responsive anemia. The intervention did not include guidance on transfusion decisions, and institutional guidelines for RBC transfusion were used in both arms (ie, hemoglobin concentration <7 g/dL, or <8 g/dL in patients with coronary ischemia or impaired oxygen delivery).

The standard care group received clinical care at the discretion of the clinical team throughout the duration of hospitalization. The anemia prevention and treatment bundle was not provided. Although clinicians were instructed not to modify clinical care for this group, pharmacological anemia treatments could be used if considered to be standard clinical practice.

Participants and clinical teams were not blinded to treatment assignment. Outcome assessors and data analysts were blinded, however.

### Outcome Measures

Outcomes were assessed at 1 month and 3 months after hospital discharge. The primary outcome was the mean difference in hemoglobin concentration at the 1-month follow-up, with differences also assessed at ICU discharge, hospital discharge, and 3-month follow-up.

Prespecified secondary outcomes, incorporating the Core Outcome Measurement Set,^27^ were patient-reported (1) quality of life, as measured with the EuroQol 5 Dimension (EQ-5D) Visual Analogue Scale (EQ-VAS) of perceived health status (score range: 0-100, with higher scores indicating better quality of life); (2) fatigue, as measured with the Functional Assessment of Chronic Illness Therapy (FACIT) Fatigue Subscale (inverted score range: 0-52, with higher scores indicating less fatigue); (3) physical function, as measured with a 6-minute walk distance (6MWD; greater distance walked indicates greater ambulatory capacity) and Katz Index of Independence in Activities of Daily Living (Katz-ADL; score range: 0-6, with higher scores indicating better physical function); (4) cognition, as measured with the Montreal Cognitive Assessment, Blind (MoCA-Blind; score range: 0-22, with higher scores indicating better cognition); and (5) mental health, as measured with the Hospital Anxiety and Depression Scale (HADS) Depression and Anxiety Subscales (score range: 0-21, with higher scores indicating greater symptom burden) and the Impact of Events Scale–Revised (IES-R) for posttraumatic stress (score range: 0-88, with higher scores indicating greater symptom burden). Each outcome was assessed at 1 month and 3 months after hospitalization.

Additionally, we evaluated differences in RBC transfusion rates, unplanned hospital readmissions, and mortality. Differences in phlebotomy frequency, volumes, and redraws were compared during hospitalization. Compliance with treatment assignment was based on appropriate receipt of anemia-directed pharmacological therapy after randomization for the intervention group.

### Statistical Analysis

Patient demographics, admission characteristics, and outcomes were summarized according to treatment group as median (IQR) for continuous variables and as frequency (percentage) for categorical variables. Baseline was defined as the time of randomization (ie, baseline laboratory and clinical variables obtained before randomization). Outcomes were evaluated using a prespecified analysis plan, as described previously.^24^ A linear mixed-effects model with random intercept and fixed effects of time point, treatment, and time point by treatment interaction term was used to assess the association between intervention and hemoglobin concentration outcomes, adjusting for baseline hemoglobin concentration, age, sex, and surgical vs nonsurgical admission. A linear combination of treatment and time point by treatment interaction term estimated the treatment effect at the 1-month follow-up.

Similarly, linear or generalized linear mixed-effects models were used to assess secondary outcomes using proportional odds models for score outcomes and logistic models for yes-or-no outcomes. Models assessing 6MWD were also adjusted for baseline activities of daily living. Missing data were assumed to be missing at random in the primary analysis that used mixed-effects models, with the assumption being the probability of missing a hemoglobin value at follow-up was potentially related to other observed data in the model, including randomized group, adjustment covariates, or other observations of hemoglobin concentration at other time points. However, missing at random assumed that the probability of missing a hemoglobin value was not related to the unobserved (missing) hemoglobin value itself. A similar assumption was made for other outcomes analyzed with mixed-effects models.

A sensitivity analysis explored a missing-not-at-random assumption for the primary outcome analysis, which included an imputation for no hemoglobin level change from baseline, with 95% CIs estimated by 1000 bootstrap samples. Participants who died prior to outcome measurement at the 1-month or 3-month follow-up were treated as censored. Model assumptions were assessed visually by plotting residuals, and no violations requiring additional methods were observed. Results were presented as estimates with 95% CIs and *P* values for the increase in mean or for the multiplicative increase in either odds of higher score or odds for the given event associated with intervention.

Using a 2-sample *t* test, we determined that 74 participants would provide the trial with 80% power (2-sided α = .05) to detect a 1-g/dL between-group difference in 1-month hemoglobin concentration based on a historical mean (SD) 1-month hemoglobin concentration of 10.8 (1.5) g/dL. The sample size was inflated to 100 to account for anticipated dropout (eg, death, loss to follow-up).

*P* < .05 was considered statistically significant. Intention-to-treat analyses were performed between July 2024 and January 2025 using R, version 4.2.2 (R Project for Statistical Computing) and SAS, version 9.4M7 (SAS Institute Inc).

## Results

### Patients and Interventions

**Figure 1.**
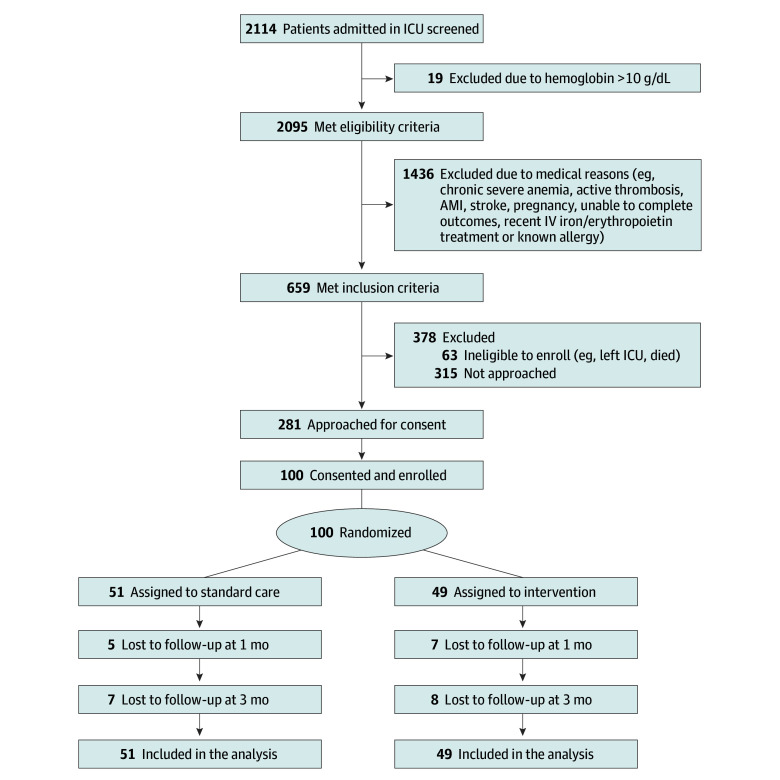
Study Flow Diagram Patients were enrolled during their index intensive care unit (ICU) admission. Those who were not enrolled during index ICU admission but were subsequently readmitted to the ICU during the index hospitalization were not eligible for enrollment. AMI indicates acute myocardial infarction; IV, intravenous.

A total of 100 patients were enrolled and included in the analysis, with 49 patients (49.0%) randomly assigned to receive the intervention and 51 (51.0%) to receive standard care (Figure 1). Participants had a median (IQR) age of 68 (61-72) years and included 43 women (43.0%) and 57 men (57.0%) (Table 1). Seventy-six patients (76.0%) were admitted to surgical ICUs, with 65 of 76 (85.5%) admitted after surgery (46 of 65 [70.8%] after cardiac surgery). Among nonoperative admissions, cardiovascular admission diagnoses were the most common (16 of 35 [45.7%]) (eTable 1 in Supplement 2). Median (IQR) hemoglobin concentration at enrollment was 8.9 (8.4-9.4) g/dL. Anemia was deemed to be iron responsive in all cases, with a median (IQR) transferrin saturation of 12.0% (8.0%-18.5%). Sixty patients (60.0%) experienced major bleeding (estimated blood loss ≥500 mL), and 46 (46.0%) received allogeneic RBCs prior to enrollment, with a median (IQR) of 2 (2-3) units. Median (IQR) ICU length of stay (LOS) was 3 (2-4) days prior to randomization, with median (IQR) postrandomization ICU LOS of 1 (0-2) day in both treatment groups. Median (IQR) postrandomization hospital LOS was 5 (3-8) days in the intervention group and 6 (4-7) days in the standard care group. One patient (2.0%) died during index hospitalization in the intervention group vs 0 in the standard care group.

**Table 1.** Participant Demographics and Admission Characteristics

Characteristic	Patients, No. (%)		
	Standard care group (n = 51)	Intervention group (n = 49)	Total (N = 100)
Age, median (IQR), y	68 (61-72)	67 (63-70)	68 (61-72)
Sex			
Female	23 (45.1)	20 (40.8)	43 (43.0)
Male	28 (54.9)	29 (59.2)	57 (57.0)
BMI, median (IQR)	30.5 (26.3-34.6)	31.1 (27.7-33.9)	30.8 (27.1-34.5)
Ethnicity^a^			
Hispanic or Latino	1 (2.0)	0	1 (1.0)
Not Hispanic or Latino	50 (98.0)	48 (98.0)	98 (98.0)
Unknown	0	1 (2.0)	1 (1.0)
Prior hospital LOS, median (IQR), d	3 (1-4)	2 (1-4)	2 (1-4)
APACHE II score, median (IQR)	17 (12-20)	14 (12-17)	14 (12-19)
SOFA score, median (IQR)	4 (2-7)	4 (2-6)	4 (2-6)
ICU admission type, surgical	38 (74.5)	38 (77.6)	76 (76.0)
Unscheduled vs elective surgical admission	18 (47.4)	18 (47.4)	36 (47.4)
ICU admitting diagnosis, postoperative	31 (60.8)	34 (69.4)	65 (65.0)
EBL ≥500 mL prior to enrollment	29 (56.9)	31 (63.3)	60 (60.0)
RBC transfusion prior to enrollment	23 (45.1)	23 (46.9)	46 (46.0)
RBC units prior to enrollment, median (IQR), No. (n = 46)	2 (2-3)	2 (2-3)	2 (2-3)
Baseline laboratory values			
Hemoglobin, median (IQR), g/dL	8.8 (8.4-9.4)	8.9 (8.3-9.2)	8.9 (8.4-9.4)
Ferritin, median (IQR), ng/mL (n = 99)	238 (136-437)	202 (126-407)	223 (129-430)
Transferrin saturation, median (IQR), % (n = 99)	12.0 (8.0-19.8)	12.0 (9.0-17.0)	12.0 (8.0-18.5)
Platelet count, median (IQR), ×10^3^/μL	158 (116-218)	143 (118-183)	149 (116-204)
WBC count, median (IQR), /μL	12 300 (8800-15 900)	11 100 (8500-14 100)	11 900 (8600-15 600)
Creatinine, median (IQR), mg/dL	0.9 (0.8-1.9)	1.0 (0.8-1.2)	1.0 (0.8-1.4)

Abbreviations: APACHE II, Acute Physiology and Chronic Health Evaluation (score range: 0-71, with the highest score indicating greater illness severity); BMI, body mass index (calculated as weight in kilograms divided by height in meters squared); EBL, estimated blood loss; ICU, intensive care unit; LOS, length of stay; RBC, red blood cell; SOFA, Sequential Organ Failure Assessment (score range: 0-24, with the highest score indicating greater illness severity); WBC, white blood cell.

SI conversion factors: To convert creatinine to micromoles per liter, multiply by 88.4; ferritin to micrograms per liter, multiply by 1.0; hemoglobin to grams per liter, multiply by 10.0; platelet count to ×10^9^ per liter, multiply by 1.0; and WBC count to ×10^9^ per liter, multiply by 0.001.

^a^ Ethnicity was self-identified by patients or their surrogates at study enrollment.

Adherence to assigned therapy was 98.0%, with 1 patient in the intervention group receiving only a partial study dose of IV iron due to inadvertent discontinuation by the clinical team. There were no infusion reactions to IV iron. No patients received erythropoietin. Six patients (11.8%) in the standard care group received IV iron later in hospitalization as directed by the clinical team, which was not considered a protocol violation.

### Trial Outcomes

#### Posthospitalization Anemia and Transfusion

**Figure 2.**
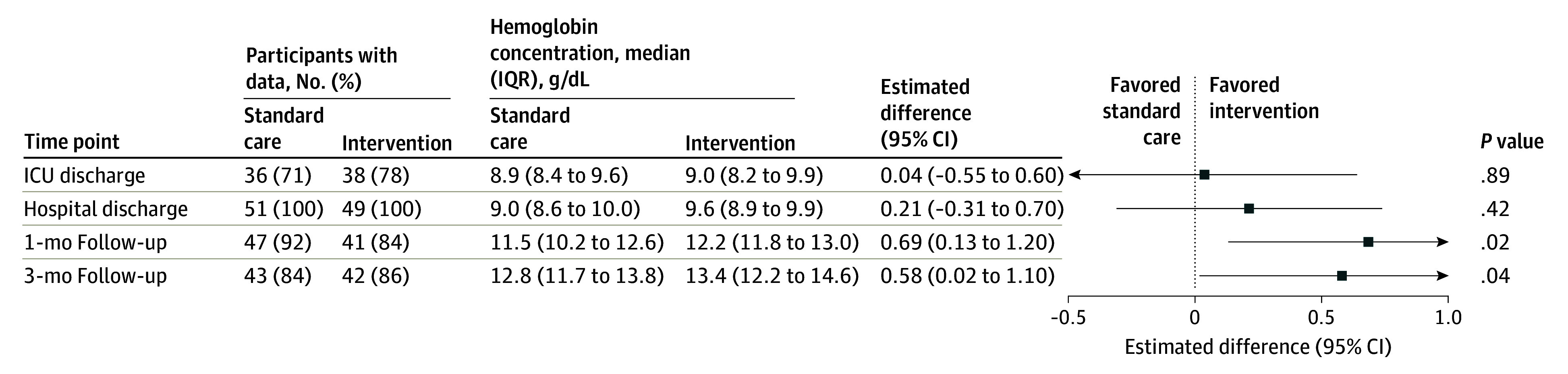
Hemoglobin Concentrations Over Time, With Primary Outcome Mean Difference at the 1-Month Follow-Up There were 26, 0, 12, and 15 participants missing intensive care unit (ICU) discharge, hospital discharge, 1-month follow-up, and 3-month follow-up hemoglobin values, respectively. Estimates were calculated using a linear mixed-effects model adjusted for baseline hemoglobin concentration, age, sex, and surgical vs medical ICU admission type. Estimates show the intervention-associated increase in hemoglobin concentration (to convert g/dL to g/L, multiply by 10.0) at a given time point.

Hemoglobin concentration at 1 month after hospital discharge (primary outcome) was significantly greater in the intervention group compared with the standard care group (median [IQR], 12.2 [11.8-13.0] g/dL vs 11.5 [10.2-12.6] g/dL; adjusted mean difference, 0.69 [95% CI, 0.13-1.20] g/dL; *P* = .02) (Figure 2). Similar findings were observed at the 3-month follow-up. Hemoglobin concentrations did not significantly differ at ICU or hospital discharge (eFigure 1 in Supplement 2). Differences in baseline and hospital features between patients with (n = 88) and without (n = 12) 1-month hemoglobin concentration assessments are provided in eTable 2 in Supplement 2. In a worst-case imputation approach, hemoglobin concentration differences were not statistically significant, with wider CIs but with a point estimate favoring the intervention (eTable 3 in Supplement 2).

Posthospitalization RBC transfusion within 3 months occurred in 1 patient (2.0%) in the intervention group vs 6 patients (11.8%) in the standard care group (*P* = .09) (Table 2). Hospital readmission within 3 months occurred in 12 participants (24.5%) receiving the intervention vs 16 participants (31.4%) receiving standard care (*P* = .48). Two patients (4.1%) in the intervention group died within 3 months vs 1 (2.0%) in the standard care group (*P* = .54). There were 13 adverse events among 11 patients (5 in intervention group; 6 in standard care group) over the 3-month follow-up (eTable 4 in Supplement 2). All adverse events were deemed unrelated or unlikely to be related to study procedures.

**Table 2.** Transfusions, Readmissions, and Mortality Up to 3 Months After Hospitalization

Outcome	Patients, No. (%)		Treatment effect^a^	
	Standard care group (n = 51)	Intervention group (n = 49)	Estimate, OR (95% CI)	*P* value
RBC transfusion	6 (11.8)	1 (2.0)	0.16 (0.02-1.40)	.09
Hospital readmission	16 (31.4)	12 (24.5)	0.73 (0.30-1.80)	.48
Mortality^b^	1 (2.0)	2 (4.1)	2.13 (0.19-24.0)	.54

Abbreviations: OR, odds ratio; RBC, red blood cell.

^a^ Linear or generalized linear mixed-effects models were used in calculations. All estimates correspond to the intervention-associated multiplicative increase in odds of a higher score or odds for a given event.

^b^ Mortality includes death during index hospitalization (n = 1 in the intervention group) and after hospitalization.

#### Phlebotomy Frequency and Volumes

Phlebotomy frequency and volumes were lower in the intervention group compared with the standard care group (eTable 5 in Supplement 2). The median (IQR) number of specimens sent for analysis was 32 (24-55) in the intervention group vs 46 (20-74) in the standard care group (*P* = .35). Median (IQR) phlebotomy volume from enrollment through hospital discharge was 32 (22-54) mL in the intervention group vs 142 (68-195) mL in the standard care group (*P* < .001). There were no differences in redraws (median [IQR], 0 [0-2] in each group; *P* = .93).

#### Posthospitalization Multidimensional Functional Recovery and Interaction Analyses

**Figure 3.**
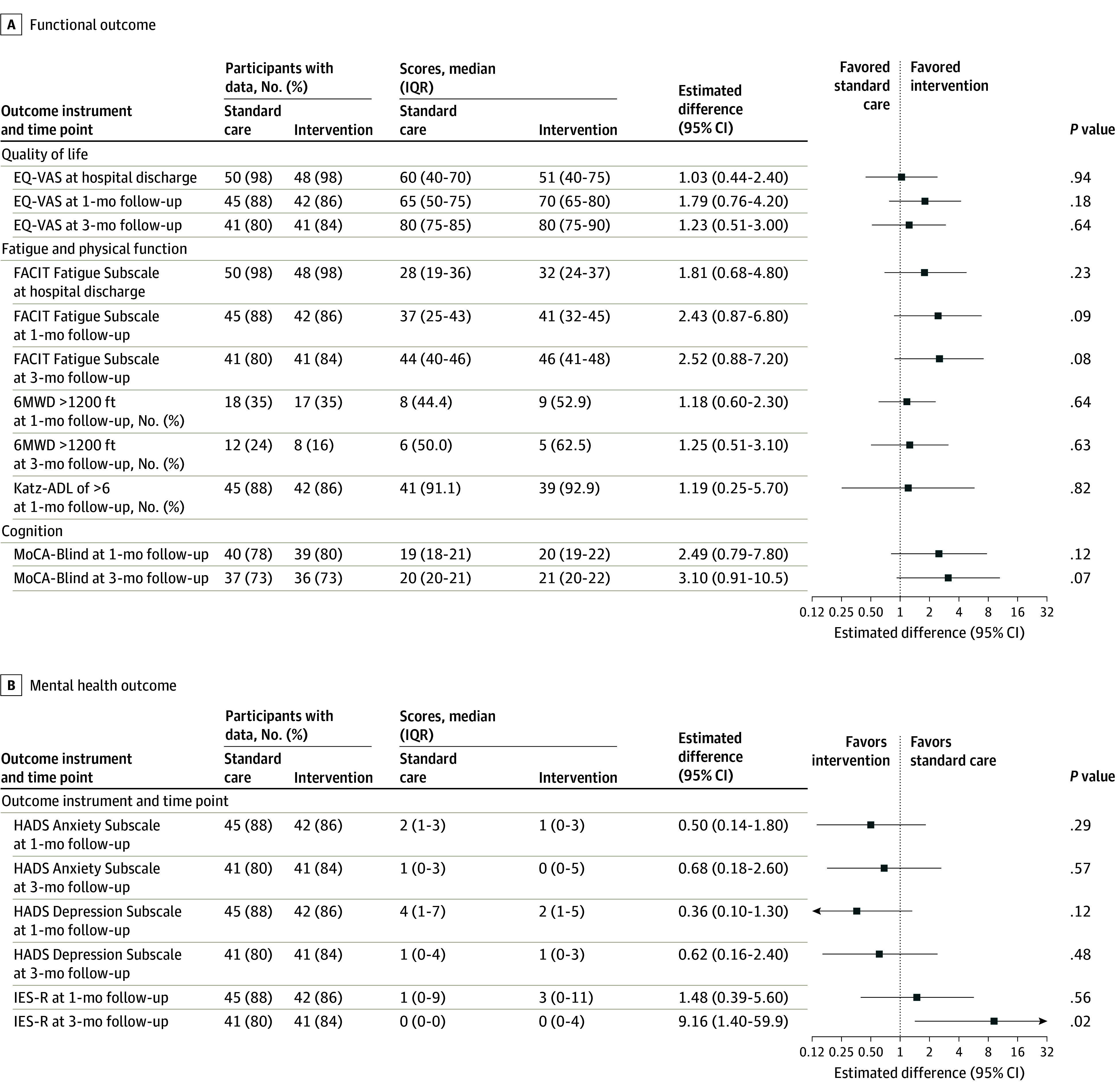
Multidimensional Functional Outcomes Up to 3 Months After Hospitalization Estimates were calculated using linear or generalized linear mixed-effects models. All estimates are odds ratios (ORs) and correspond to the intervention-associated multiplicative increase in odds of a higher score or odds for the given event. Quality of life and fatigue and physical function outcomes were also adjusted for baseline Katz Index of Independence in Activities of Daily Living (Katz-ADL; score range: 0-6, with higher scores indicating better physical function). 6MWD indicates 6-minute walk distance (greater distance walked indicates greater ambulatory capacity); EQ-VAS, EuroQol Visual Analogue Scale (score range: 0-100, with higher scores indicating better quality of life); FACIT, Fatigue Subscale, Functional Assessment of Chronic Illness Therapy (inverted score range: 0-52, with higher scores indicating less fatigue); HADS, Hospital Anxiety and Depression Scale (score range: 0-21, with higher scores indicating greater symptom burden); IES-R, Impact of Events Scale–Revised (score range: 0-88, with higher scores indicating greater symptom burden); and MoCA-Blind, Montreal Cognitive Assessment, Blind (score range: 0-22, with higher scores indicating better cognition).

Functional outcomes did not significantly differ between the intervention and standard care groups at 1 month after discharge (Figure 3). Median (IQR) EQ-VAS scores were 70 (65-80) vs 65 (50-75), respectively, with the odds ratio (OR) for whether treatment was associated with higher scores being 1.79 (95% CI, 0.76-4.20; *P* = .18); EQ-5D-3 level domain scores are provided in eTable 6 in Supplement 2. Median (IQR) FACIT Fatigue Subscale inverted scores were 41 (32-45) vs 37 (25-43), respectively (OR, 2.43; 95% CI, 0.87-6.80; *P* = .09). Median (IQR) 6MWD distance was 1211 (960-1480) feet vs 1080 (790-1250) feet, respectively (adjusted difference, 218 [95% CI, –72.7 to 510] feet; *P* = .13]; to convert to meters, multiply by 0.3) (eFigure 2 in Supplement 2). Median (IQR) MoCA-Blind scores were 20 (19-22) vs 19 (18-21), respectively (OR, 2.49; 95% CI, 0.79-7.80; *P* = .12). Median (IQR) HADS Depression Subscale scores were 2 (1-5) and 4 (1-7), respectively (OR, 0.36; 95% CI, 0.10-1.30; *P* = .12). Median (IQR) HADS Anxiety Subscale scores were 1 (0-3) and 2 (1-3), respectively (OR, 0.50; 95% CI, 0.14-1.80; *P* = .29). Median (IQR) IES-R scores were 3 (0-11) and 1 (0-9), respectively, (OR, 1.48; 95% CI, 0.39-5.60; *P* = .56). Similar findings were observed at the 3-month follow-up (Figure 3). For the primary outcome of hemoglobin concentration at the 1-month follow-up, there were no significant interactions between treatment group and age, sex, or ICU admission type (eTable 7 in Supplement 2).

## Discussion

Critically ill adults who were randomly assigned to an anemia prevention and treatment bundle vs standard care experienced greater posthospitalization hemoglobin recovery. Although the trial was not powered to detect changes in secondary outcomes reflecting multidimensional functional recovery, the intervention was consistently favored in point estimates for quality of life, fatigue, physical function, cognition, depressive symptoms, and anxiety, providing requisite information for a subsequent multicenter clinical trial.

These findings build on previous work evaluating the effects of IV iron after surgery and during critical illness. Several small trials in various postsurgical populations have shown that IV iron effectively augments hemoglobin recovery,^28,29,30^ although with limited assessments of clinical outcomes and without incorporation of anemia prevention strategies. In critically ill populations specifically (including both surgical and medical patients), a multicenter, placebo-controlled trial found that IV iron administered during critical illness improved hemoglobin concentrations by 0.7 g/dL by hospital discharge but had no significant impact on inpatient transfusion use.^19^ More recently, a multicenter feasibility trial comparing IV iron administration at ICU discharge vs usual care found greater hemoglobin recovery (mean difference, 1.1 [95% CI, 0.5-1.7] g/dL) up to 3 months after randomization.^31^ These results are consistent with findings in the present trial and those of a recent meta-analysis showing improved hemoglobin recovery through at least 30 days after discharge among critically ill patients who received IV iron during inpatient admission.^32^ Hemoglobin recovery in response to iron replacement is not instantaneous, with this trial’s findings highlighting that most recovery will occur after index hospitalization. This notion is supported by numerically lower transfusion rates in the intervention vs standard care group through the first 3 months after hospitalization (2.0% vs 11.8%), which accompanied improved posthospitalization hemoglobin recovery.

Previous trials primarily evaluated changes in hemoglobin concentration and/or RBC transfusion use as outcomes. To our knowledge, this trial was the first to evaluate, albeit in exploratory fashion, multiple prespecified secondary outcomes representative of multidimensional functional recovery. Survival from surgery and critical illness does not equate to meaningful recovery of function. We hypothesized that anemia prevention and treatment would improve patient-centered outcomes in addition to hemoglobin recovery. Although this trial was not powered to detect significant differences, effect estimates suggest that anemia treatment may indeed have beneficial effects on posthospitalization fatigue, quality of life, physical function, cognition, and symptoms of depression and anxiety. These findings are encouraging given the known associations between anemia and/or iron deficiency and multidimensional functional impairments across numerous patient groups and practice environments.^12,21,33,34,35,36,37,38^ Although statistically higher posttraumatic stress symptoms (measured with IES-R) were observed at the 3-month follow-up in the intervention group, symptoms were low in both groups (median values of 0), making it unlikely that effects were causally associated with the intervention. Definitive evaluation of how anemia management affects functional outcomes will require adequately powered clinical trials, with the data from this trial providing effect size estimates for trial design.

Nonetheless, the pattern of improvements in functional recovery is consistent with findings in prior literature. Improvements in physical function (eg, greater ambulatory distance) have generally accompanied IV iron administration in heart failure trials,^39,40,41,42^ whose populations may have considerable overlap with this trial’s population (>50% admitted after cardiac surgery or had cardiac admission diagnoses). Differences in 6MWD in this trial, although not statistically significant and limited in patient numbers, exceeded the minimal clinically important difference described in patients with iron deficiency and heart failure.^43^ Additionally, findings of trials of IV iron during recovery from critical illness suggest potential beneficial effects on hospital readmissions and postdischarge mortality.^18,31^ In this trial, we found nonsignificant lower readmissions among treated patients (24.5% in the intervention group vs 31.4% in the standard care group).

Regarding anemia prevention, observational and clinical trial data highlight that low-volume blood collection tubes may modestly attenuate hemoglobin concentration decreases and RBC transfusion during acute illness without increasing the frequency of blood redraws.^44,45,46^ In this trial, the intensity of phlebotomy was reduced through the innovative application of visual and electronic clinician-decision aids encouraging clinicians to reconsider the need for laboratory assessments, bundling of similarly timed laboratory studies, closed-loop phlebotomy, and low-volume blood sampling (ie, filling standard blood tubes to the minimum volume required for automated laboratory processing). Regarding low-volume blood sampling, this process did not require the introduction of new sampling tubes, which has potential implications for resource acquisition and compatibility with automated laboratory processes. There were no increases in redraws, suggesting that patient safety can be preserved while simultaneously reducing iatrogenic blood loss. Hence, optimization of phlebotomy practice should be pursued for all critically ill patients. Alternatively, future trials may consider the Multiphase Optimization Strategies method to evaluate the individual components of a multifaceted anemia bundle.

### Limitations

This trial has several limitations. First, the intervention assignment was not blinded to clinicians and patients, which may have influenced clinical decision-making by ICU team members or self-reported patient outcomes. Second, the trial was small, not powered to detect differences in functional outcomes, and not adjusted for multiple comparisons, elevating the risk of type I error; however, we obtained important information for the design of a large multicenter trial. Third, most patients experienced surgery or major bleeding, and all patients were deemed to have iron-responsive anemias for which they received IV iron. While this finding reflects acute blood loss and iron deficiency as major factors of ICU-acquired anemia, the effectiveness and safety of erythropoietin were unexplored. Additionally, ferritin and transferrin saturation values may be affected by inflammation, making actual iron deficits unclear for any given participant. Fourth, ICU and hospital LOSs were modest, with most participants receiving the intervention toward the end of their ICU stay. It is unclear if earlier enrollment would have changed treatment effects. Additionally, the treatment bundle did not extend after discharge. Fifth, we did not evaluate the acceptability of the anemia prevention and treatment bundle among ICU team members. Sixth, although we were pleased that more than one-third of the patients that we approached consented to participate, considering the nature of their critical illness, this proportion raises the possibility of participation bias. We also excluded certain patients who may have potentially benefited from the intervention (eg, those with severe prehospitalization anemia). Seventh, we were unable to distinguish potential differential effects of the various components of the bundle. Eighth, the single-center design may limit the generalizability of results. Nevertheless, we have shown that implementation of an anemia treatment bundle was feasible within our practice and was accompanied by improvement in hemoglobin recovery without adverse effects.

## Conclusions

A multifaceted anemia prevention and treatment bundle delivered predominantly during postsurgical critical illness was feasible, well tolerated, and improved hemoglobin concentrations up to 3 months after hospital discharge. We obtained effect size estimates for how anemia management affects multiple functional domains; these estimates can inform the design of future trials evaluating how anemia management strategies may improve patient outcomes after critical illness. Key outcomes to consider include those related to quality of life, fatigue, physical function, cognition, depression, and anxiety. Future trials may also explore optimal intervention timing, the role of erythropoiesis-stimulating agents or other therapies, and cost-effectiveness across more diverse patient populations.

## Notes

Supplement 1.
Trial Protocol and Statistical Analysis Plan

Supplement 2. **eTable 1.** Additional Patient and Hospitalization Characteristics **eTable 2.** Demographic, Clinical, and Laboratory Features for Those With and Without 1-Month Post-Hospitalization Hemoglobin Assessments **eTable 3.** Estimated Treatment Effect for Hemoglobin Using Worst-Case Imputation (No Change From Baseline) Approach **eTable 4.** Adverse Events **eTable 5.** Phlebotomy Summaries **eTable 6.** EQ-5D-3L Domain Scores **eTable 7.** Interaction Analyses **eFigure 1.** Hemoglobin Values Over Time for Individual Participants and Study Groups **eFigure 2.** 6-Minute Walk Distances at 1- and 3-Months

Supplement 3.
Data Sharing Statement
