# Supplementary material for: Practical Anemia Bundle and Hemoglobin Recovery in Critical Illness: A Randomized Clinical Trial
Source: JAMA Netw Open. 2025 Mar 28;8(3):e252353. doi: 10.1001/jamanetworkopen.2025.2353 (PMC11953759; doi:10.1001/jamanetworkopen.2025.2353)
Supplement: Supplement 1. — Trial Protocol and Statistical Analysis Plan [file jamanetwopen-e252353-s001.pdf]

The Practical Anemia Bundle for Sustained Blood Recovery (PABST-BR) in Critical Illness Randomized Clinical Trial

Protocol Number: IRB # 21-006511

Principal Investigator: Matthew A. Warner, MD

Funded by: NHLBI

Initial version: 5 October 2021

Table of Contents

STATEMENT OF COMPLIANCE 1
1 PROTOCOL SUMMARY 1
1.1 Synopsis 1
1.2 Schema 3
1.3 Schedule of Activities (SoA) 4
2 INTRODUCTION 4
2.1 Study Rationale 4
2.2 Background 5
2.3 Risk/Benefit Assessment 7
2.3.1 Known Potential Risks 7
2.3.2 Known Potential Benefits 8
2.3.3 Assessment of Potential Risks and Benefits 8
3 OBJECTIVES AND ENDPOINTS 10
4 STUDY DESIGN 11
4.1 Overall Design 11
4.2 Scientific Rationale for Study Design 13
4.3 Justification for Dose 13
4.4 End of Study Definition 13
5 STUDY POPULATION 13
5.1 Inclusion Criteria 13
5.2 Exclusion Criteria 14
5.3 Lifestyle Considerations 14
5.4 Screen Failures 14
5.5 Strategies for Recruitment and RetentioN 15
6 STUDY INTERVENTION 16
6.1 Study Intervention(s) Administration 16
6.1.1 Study Intervention Description 16
6.2 Preparation/Handling/Storage/Accountability 17
6.2.1 Acquisition and accountability 17
6.2.2 Formulation, Appearance, Packaging, and Labeling 17
6.2.3 Product Storage and Stability 17
6.2.4 Preparation 18
6.3 Measures to Minimize Bias: Randomization and Blinding 18
6.4 Study Intervention Compliance 18
6.5 Concomitant Therapy 18
6.5.1 Rescue Medicine 18
7 STUDY INTERVENTION DISCONTINUATION AND PARTICIPANT DISCONTINUATION/WITHDRAWAL. 18
7.1 Discontinuation of Study Intervention 18
7.2 Participant Discontinuation/Withdrawal from the Study 19

7.3 Lost to Follow-Up 19
8 STUDY ASSESSMENTS AND PROCEDURES 19
8.1 Efficacy Assessments 19
8.2 Safety and Other Assessments 20
8.3 Adverse Events and Serious Adverse Events 20
8.3.1 Definition of Adverse Events (AE) 20
8.3.2 Definition of Serious Adverse Events (SAE) 21
8.3.3 Classification of an Adverse Event 21
8.3.4 Time Period and Frequency for Event Assessment and Follow-Up 22
8.3.5 Adverse Event Reporting 22
8.3.6 Serious Adverse Event Reporting 22
8.3.7 Reporting Events to Participants 23
8.3.8 Events of Special Interest 23
8.3.9 Reporting of Pregnancy 23
8.4 Unanticipated Problems 23
8.4.1 Definition of Unanticipated Problems (UP) 23
8.4.2 Unanticipated Problem Reporting 23
8.4.3 Reporting Unanticipated Problems to Participants 24
9 STATISTICAL CONSIDERATIONS 24
9.1 Statistical Hypotheses 24
9.2 Sample Size Determination 24
9.3 Populations for Analyses 24
9.4 Statistical Analyses 25
9.4.1 General Approach 25
9.4.2 Analysis of the Primary Efficacy Endpoint(s) 25
9.4.3 Analysis of the Secondary Endpoint(s) 26
9.4.4 Safety Analyses 26
9.4.5 Baseline Descriptive Statistics 26
9.4.6 Planned Interim Analyses 26
9.4.7 Sub-Group Analyses 26
9.4.8 Tabulation of Individual participant Data 27
9.4.9 Exploratory Analyses 27
10 SUPPORTING DOCUMENTATION AND OPERATIONAL CONSIDERATIONS 27
10.1 Regulatory, Ethical, and Study Oversight Considerations 27
10.1.1 Informed Consent Process 27
10.1.2 Study Discontinuation and Closure 27
10.1.3 Confidentiality and Privacy 28
10.1.4 Future Use of Stored Specimens and Data 29
10.1.5 Key Roles and Study Governance 29
10.1.6 Safety Oversight 29
10.1.7 Clinical Monitoring 29

10.1.8 Quality Assurance and Quality Control 29
10.1.9 Data Handling and Record Keeping 30
10.1.10 Protocol Deviations 30
10.1.11 Publication and Data Sharing Policy 31
10.1.12 Conflict of Interest Policy 31
10.2 Additional Considerations 31
10.3 Abbreviations 34
10.4 Protocol Amendment History ...... **Error! Bookmark not defined.**
11 REFERENCES 36

STATEMENT OF COMPLIANCE

The trial will be carried out in accordance with International Conference on Harmonisation Good Clinical Practice (ICH GCP) and the following:

- United States (US) Code of Federal Regulations (CFR) applicable to clinical studies (45 CFR Part 46, 21 CFR Part 50, 21 CFR Part 56, 21 CFR Part 312, and/or 21 CFR Part 812)

National Institutes of Health (NIH)-funded investigators and clinical trial site staff who are responsible for the conduct, management, or oversight of NIH-funded clinical trials have completed Human Subjects Protection and ICH GCP Training.

The protocol, informed consent form(s), recruitment materials, and all participant materials will be submitted to the Institutional Review Board (IRB) for review and approval. Approval of both the protocol and the consent form must be obtained before any participant is enrolled. Any amendment to the protocol will require review and approval by the IRB before the changes are implemented to the study. In addition, all changes to the consent form will be IRB-approved; a determination will be made regarding whether a new consent needs to be obtained from participants who provided consent, using a previously approved consent form.

1 PROTOCOL SUMMARY

1.1 SYNOPSIS

Title: The Practical Anemia Bundle for SusTained Blood Recovery (PABST-BR) in Critical Illness Randomized Clinical Trial

Study Description: The goal of this investigation is to test a multi-faceted anemia prevention and targeted treatment bundle (optimized phlebotomy, decision support, targeted pharmacologic treatment) to attenuate anemia development and promote hemoglobin and functional recovery in adults with critical illness. We hypothesize that the bundle will improve hemoglobin recovery and functional outcomes through 3 months post-hospitalization.

Objectives: Primary Objective: To assess the efficacy of the intervention on mean difference in hemoglobin concentrations at 1 month after hospitalization.

Secondary Objectives:

To assess the impact of the intervention on:

- hemoglobin concentrations through 3-months post hospitalization
- functional outcomes (Core Outcome Measurement Set [COMS], PROMIS-FATIGUE) at 1 and 3 months post-hospitalization
- RBC transfusions through 3 months post-hospitalization
- hospital readmissions through 12 months post-hospitalization
- mortality through 12 months post-hospitalization

Endpoints: Primary Endpoint: Hemoglobin concentrations (mean difference in hemoglobin concentrations at 1-month post-hospitalization)

Secondary Endpoints:

- Hemoglobin concentrations (through 3 months post-hospitalization)
- Functional outcomes (EQ-5D, PROMIS-FATIGUE, 6-minute walk distance, activities of daily living survey, MOCA-BLIND, HADS, IES-R) at 1 and 3-months postop-hospitalization
- RBC transfusions through 3-months post-hospitalization

	• Readmissions in first 12-months post-hospitalization<br>• All-cause mortality through 12 months post-hospitalization
**Study Population:**	100 patients, male and females, age ≥ 18 years, moderate-to-severe comorbid illness burden, moderate-to-severe acute illness burden, in ICUs at Mayo Clinic Rochester
**Phase:**	2
**Description of Sites/Facilities Enrolling Participants:**	ICUs at Mayo Clinic Rochester, MN; a large tertiary care academic medical center
**Description of Study Intervention:**	Participants will be randomized 1:1 to active intervention vs. standard of care using a stratified permuted block design by anemia type (iron-responsive vs. inflammatory) and ICU admission indication (surgical vs. non-surgical).<br><br>The intervention arm is multi-faceted with 3 primary components:<br>1) **Optimized phlebotomy**, defined by:<br>• minimal volume draws<br>• closed-loop blood sampling<br>• bundling of similarly timed labs, all performed by a dedicated phlebotomy team independent from the treatment team<br><br>2) **Decision support aids**, including:<br>• visual and electronic alerts reminding the care team to minimize non-essential laboratory testing, mitigate patient-specific bleeding risk<br>• daily communication with the ICU care team during clinical rounds to reiterate the purpose of the study<br><br>3) **Pharmacologic anemia treatment** (given immediately following randomization) targeted to 2 broad groups:<br>1) Anemia responsive to iron supplementation (i.e. 1000 mg IV low molecular weight iron dextran), and<br>2) Anemia of inflammation requiring erythropoietic stimulation (i.e. 40,000 units of subcutaneous erythropoietin +/- iron supplementation as needed to augment iron stores prior to EPO dosing)
**Study Duration:**	2 years
**Participant Duration:**	3 months post-hospitalization, EMR review by study staff at 12 months post-hospitalization

1.2 SCHEMA

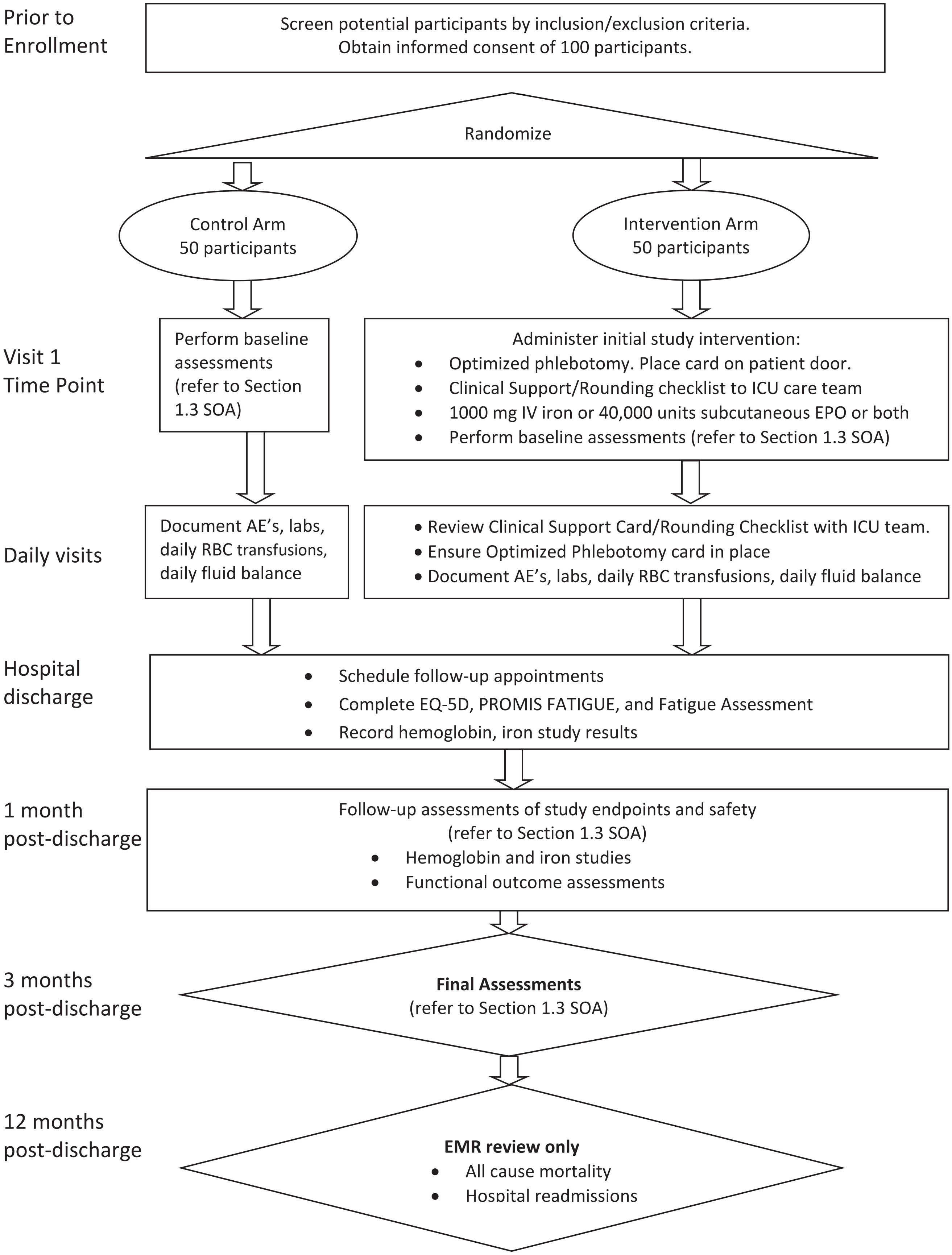

Prior to Enrollment
Screen potential participants by inclusion/exclusion criteria.
Obtain informed consent of 100 participants.
Randomize
Control Arm
50 participants
Intervention Arm
50 participants
Visit 1 Time Point
Perform baseline assessments (refer to Section 1.3 SOA)
Administer initial study intervention:
Optimized phlebotomy. Place card on patient door.
Clinical Support/Rounding checklist to ICU care team
1000 mg IV iron or 40,000 units subcutaneous EPO or both
Perform baseline assessments (refer to Section 1.3 SOA)
Daily visits
Document AE's, labs, daily RBC transfusions, daily fluid balance
Review Clinical Support Card/Rounding Checklist with ICU team.
Ensure Optimized Phlebotomy card in place
Document AE's, labs, daily RBC transfusions, daily fluid balance
Hospital discharge
Schedule follow-up appointments
Complete EQ-5D, PROMIS FATIGUE, and Fatigue Assessment
Record hemoglobin, iron study results
1 month post-discharge
Follow-up assessments of study endpoints and safety
(refer to Section 1.3 SOA)
Hemoglobin and iron studies
Functional outcome assessments
3 months post-discharge
Final Assessments
(refer to Section 1.3 SOA)
12 months post-discharge
EMR review only
All cause mortality
Hospital readmissions

1.3 SCHEDULE OF ACTIVITIES (SOA)

Procedures	Enrollment/ Baseline Study day 1	Daily visits throughout hospitalization	Hospital Discharge	1-month post-hospitalization +/- 7 days	3-months post-hospitalization +/- 14 days	12-months post-hospitalization EMR review
Informed consent	X					
Demographics	X					
Medical history	X					
Randomization	X					
Administer study intervention	X					
Vital signs- (BP, HR, RR, O2 sat)	X					
Height	X					
Weight	X		X			
Pregnancy test if needed	X					
Anemia labs[a]	X	X	X	X	X	
Platelet count[d]	X		X			
White blood cell count[d]	X		X			
Creatinine[d]	X		X			
Functional outcome assessment [b]	X		X	X	X	
Con medication review [c]	X	X				
Fluid balance	X		X			
RBC transfusions documented	X	X	X	X	X	
Daily phlebotomies for labs (excluding RMGs)		X	X			
Fatigue assessment			X	X	X	
AE review and evaluation	X	X	X	X	X	
Research Samples[e]	X			X	X	
Hospital readmissions documentation				X	X	X
All cause mortality status						X
Complete Case Report Forms	X	X	X	X	X	X

a. **Hemoglobin, ferritin, and transferrin saturation will be obtained for all patients at enrollment, hospital discharge, 1-month, and 3-months post-hospitalization.** Other anemia labs to be reported at these and other timepoints include, if available Hemoglobin, MCV, RDW, ferritin, iron, transferrin saturation, reticulocyte hemoglobin, absolute reticulocyte count; record all values available recognizing that not all non-hemoglobin values will be available at each interval.
b. Functional outcomes according to the Core Outcome Measurement Set will be obtained in the following fashion: **Enrollment** – ADL survey (by patient or proxy); at **Hospital discharge** – EQ-5D, PROMIS-FATIGUE; **at 1-month and 3-months** – EQ-5D, PROMIS-FATIGUE, 6MWD, ADL Survey, MoCA-BLIND, HADS, IES-R, activity monitoring (optional study component).
c. Concomitant meds review to include y/n for antiplatelet agents (i.e. aspirin, clopidogrel), anticoagulants (i.e. heparin, warfarin, direct oral anticoagulants, low molecular weight heparins), iron, and erythropoiesis stimulating agent use (i.e. darbepoetin, EPO). Daily visits only need to report if they received iron or EPO/darbepoetin.
d. Platelets, WBC and Creatinine will be collected if done as standard of care. Baseline results used should be from within 24 hrs prior to enrollment. Hospital discharge results used should be the closest to discharge up to 72 hours prior.
e. Research samples (10 ml phlebotomy) will be obtained at enrollment, 1-month, and 3-months and stored for potential biomarker assessment (i.e. hepcidin, IL-6, C-reactive protein) for future mechanistic studies on anemia development and recovery in critical illness survivors.

2 INTRODUCTION

2.1 STUDY RATIONALE

Anemia is common in the critically ill and is associated with poor patient outcomes both during and after hospitalization. Recent data suggests that anemia may also represent a potentially modifiable risk factor for impaired post-hospitalization physical function. The goal of this investigation is to test a multi-faceted anemia prevention and targeted treatment bundle (optimized phlebotomy practice, decision support, targeted pharmacologic anemia treatment) to attenuate anemia development and promote hemoglobin and functional recovery in the setting of critical illness. Specifically, we aim to assess the impact of the intervention on hemoglobin concentrations and functional outcomes (i.e. physical, cognitive, mental health) through 3-months after hospitalization.

2.2 BACKGROUND

Anemia is remarkably common in the critically ill.[1–3] Over the last 20 years, clinicians have become more tolerant of anemia during hospitalization and critical illness, a phenomenon driven in large part by the landmark Transfusion Requirements in Critical Care (TRICC) trial,[4] which revealed similar 30-day mortality with restrictive versus liberal red blood cell (RBC) transfusion strategies. However, we now realize that survival of critical illness does not guarantee the quality of the life preserved. Up to 50% of survivors have substantial functional deficits in one or multiple domains related to physical function, cognition, mental health, and quality of life.[5] This is increasingly relevant given the growing number of survivors of COVID-19-related critical illness.[6] It is therefore paramount that we identify modifiable risk factors for impaired functional recovery in ICU survivors, of which anemia may be one potential target.[7] Importantly, anemia has consistently been linked with impaired functional outcomes in non-critically ill populations, including the elderly,[8–11] post-surgical patients,[12–14] and those with hematologic disease[15,16] and menstrual bleeding.[17] However, our knowledge of post-hospitalization recovery from anemia in survivors of critical illness and its relationship with patient outcomes is limited, despite increasing anemia prevalence at the time of hospital discharge.[3,18,19]

Regarding post-hospitalization hemoglobin recovery, a prospective investigation of 19 critically ill patients with anemia discovered that approximately 50% remained anemic 6-months later.[20] In our data from 6460 survivors of critical illness enrolled in large population-based health study,[21,22] 80% of subjects were discharged from the hospital with anemia.[3] At 12 months post-hospitalization, only half of those alive with available hemoglobin assessments had recovered to non-anemic status, with recovery varying in accordance with anemia severity at hospital discharge. These data confirm that for many patient's anemia persists long after the resolution of critical illness. Additionally, higher hemoglobin concentrations at hospital discharge were associated with reduced post-hospitalization mortality in adjusted analyses (HR 0.95 [95% CI 0.90-0.99], per 1 g/dL increase; p=0.020), suggesting that anemia may have important implications for downstream clinical outcomes. Preliminary data from this cohort also shows that hemoglobin concentrations are strongly associated with unplanned hospital readmissions in the first 30-days after hospital discharge, with each 1 g/dL increase in hemoglobin associated with a 15% reduction in the instantaneous hazard for readmission after multivariable adjustment (HR 0.85, 95% CI 0.78, 0.93; p< 0.001; unpublished). Patient readmission status over time by the severity of anemia at hospital discharge for critical illness survivors is shown in Figure 1.

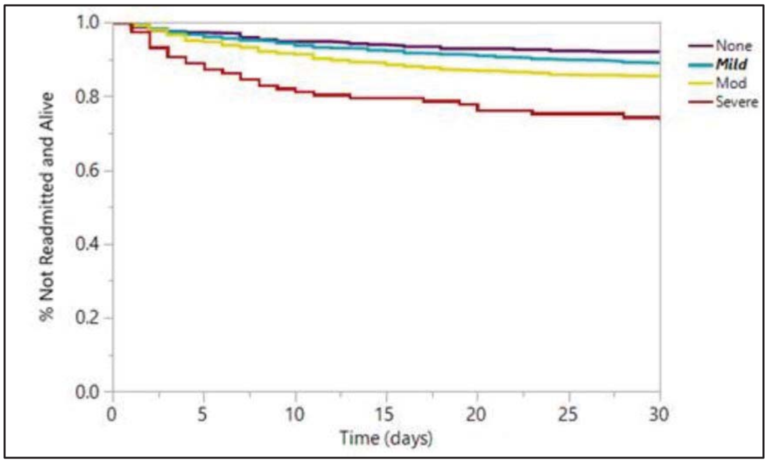

Figure 1. The percentage of patients still alive and not readmitted to the hospital (y-axis) over time (x-axis) is shown for critical illness survivors without anemia (hemoglobin >12 g/dL females, >13 g/dL males) and with mild (hemoglobin 10-12 g/dL female, 10-13 g/dL male), moderate (hemoglobin 8-10 g/dL), and severe anemia (hemoglobin < 8 g/dl) at hospital discharge.

While the relationships between anemia and functional recovery after critical illness remain incompletely defined, our recent data from a multi-center prospective cohort of 195 survivors of acute respiratory distress syndrome discovered that anemia after critical illness was associated with impaired physical function 3-months later,[7] including reductions in ambulatory capacity (i.e. 6-minute walk distance [6MWD]) and activities of daily living (ADLs). This suggests that anemia may represent a modifiable risk factor for improved physical outcomes after critical illness. However, relationships with other functional outcomes (cognition, mental health, quality of life) remain unknown, and it is unclear what effect the extent and timing of recovery from anemia after critical illness may have on outcomes. These multidimensional functional outcomes have been deemed of the highest importance for contemporary critical illness research.[23]

Anemia management strategies include prevention, attenuation, and treatment. Prevention and attenuation strategies are largely related to minimizing iatrogenic anemia development or progression secondary to phlebotomy and hemodilution. Most prominently, this includes the use of low-volume blood sampling strategies. Observational suggests that these low-volume blood draw strategies may decrease iatrogenic blood loss and transfusions,[24] though clinical trial data is limited.[25,26] In addition to low-volume phlebotomy, the use of clinical decision support is another method to promote appropriate lab utilization and to minimize excessive blood draws, though this has not been formally studied in critically ill patients. Regarding non-transfusion-based anemia treatment options, iron and erythropoietin (EPO) have been used in numerous clinical trials in the setting of critical illness.[27–32] While these therapies have consistently augmented hemoglobin recovery during hospitalization, they have had inconsistent results on RBC reductions and mortality, for which reason they have not been widely adopted into clinical practice. However, we now recognize that survival of hospitalization is not the final hurdle for critical illness survivors, and there is increasing recognition that anemia may contribute to persistent post-hospitalization impairments in daily functioning.[7] As such, strategies to augment hemoglobin recovery during critical illness may favorably influence post-hospitalization outcomes, though this remains incompletely studied. In one recent randomized clinical trial of intravenous iron with or without EPO therapy administered at the time of ICU discharge versus standard care, there was no difference between groups in the primary outcome of post-hospitalization length of stay. However, patients receiving the treatment had a significant reduction in 90-day mortality (17% vs. 8%). Additionally, there is growing evidence in surgical patients that anemia treatment can have positive consequences for patients that extend beyond hospitalization.[33] Hence, future research is clearly warranted.

Briefly, this is a randomized clinical trial of a multifaceted anemia prevention and treatment bundle versus standard of care to assess the impact of the intervention on hemoglobin recovery and post-hospitalization functional outcomes. Each patient randomized to the intervention will receive 3 treatment components: 1) optimized phlebotomy practice; 2) clinical decision support; and 3) pharmacologic anemia treatment. With regards to pharmacologic anemia treatment, patients may receive either a single dose of intravenous (IV) iron therapy in isolation if they have an iron-responsive anemia or a single dose of EPO if they have an anemia of inflammation requiring erythropoietic stimulation. Further, some patients receiving EPO may also receive a single dose of IV iron to replenish iron stores prior to EPO administration

(i.e. if serum ferritin is < 1000 ng/ml at the time of randomization). Patients randomized to the standard of care arm will receive usual ICU cares.

2.3 RISK/BENEFIT ASSESSMENT

2.3.1 KNOWN POTENTIAL RISKS

There is minimal patient risk associated with the employment of 1) optimized phlebotomy practices, or 2) decision-support aids. Changes to phlebotomy practice include: default low-volume blood sampling (e.g. 0.1-2 ml for most lab draws rather than 3-10 ml), which exposes the patient to no direct risk, and utilization of closed-loop blood sampling systems in order to minimize blood draw waste, again with no direct risk to the patient. Low-volume blood sampling is already used clinically in our ICU environments for patients with severe anemia. In rare circumstances (<1%), a low-volume sample may be insufficient for laboratory testing and additional phlebotomy may be required. Closed-loop blood sampling for those with pre-existing arterial or central venous catheters provides a method of returning "waste blood" directly to the patient rather than disposing of this "waste" volume, which typically is 10 ml per sample. It is routinely used throughout our pediatric ICU practices with no harm to patients, including no increased risk of infection. It has not been adopted into adult ICU practice given higher cost. At 1 and 3-month follow-up, patients will again undergo phlebotomy testing. There is a risk of patient discomfort with the additional blood draws at these follow-up assessments, which will be disclosed at the time of enrollment. Patients will retain the right to refuse these blood draws or any other follow-up measures. Additionally, a total of 5cc of blood will be removed for laboratory sampling at each follow-up assessment; this is not expected to have any substantial negative clinical consequence.

The third component of the clinical intervention is the utilization of EPO and/or iron for the treatment of anemia, which does carry tangible patient risks. **Iron** will be administered as a single 1000 mg dose of IV low molecular-weight iron dextran. This is an approved therapy for patients with iron-deficiency anemia that is utilized in current clinical practice for critically ill patients with anemia and contraindications to transfusion therapies (i.e. Jehovah's witness patients) and post-surgical patients after large volume blood loss. The estimated incidence of SAEs with newer IV iron formulations, such as low molecular weight iron dextran, is less than 1 in 250,000 administrations.[34] Nevertheless, immediate risks of IV iron are real and include allergic reactions (<1%) and non-allergic infusions reactions (e.g. myalgias, arthralgias, dizziness, <1%). Additionally, as a long-term risk, repeated doses of iron administration, particularly to patients with iron storage disorders (i.e. hemochromatosis) or those requiring frequent and recurrent RBC transfusions, can culminate in iron overload. Patients with hemochromatosis or elevated iron stores (i.e. ferritin > 1000 ng/ml) will not be eligible to receive iron therapies in this study.

EPO (i.e. Epoetin alpha), the erythropoiesis stimulating agent utilized in this trial, will be administered as a single 40,000 unit subcutaneous injection after iron supplementation, though iron supplementation will be withheld for those with ferritin > 1000 ng/ml. It has been used in previous clinical trials of anemia management in critical illness, and it is utilized in our current clinical practice for critically ill anemic and post-surgical patients with contraindications to transfusion therapies (i.e. Jehovah's witness patients). There are short-term risks associated with this therapy, including minor non-allergic adverse reactions (nausea, dizziness, high blood pressure, pruritis; estimated <10%) and major rare adverse reactions (e.g. deep venous thrombosis, uncontrolled hypertension, myocardial infarction; estimated <1%). There is a black box warning for myocardial infarction, stroke, venous thromboembolism, vascular access thrombosis, and mortality when targeting hemoglobin levels >11 g/dL, data which is derived from <u>repeated</u> use of EPO to achieve near-normal hemoglobin levels for patients with chronic kidney disease. In a recent systematic review and meta-analysis of 21 trials in critical illness, the relative risk (RR) for mortality with EPO was 0.82 (95% CI 0.71-0.94).[35] There were no significant differences in serious adverse events (RR 1.11, 95% CI 0.94-1.31) or VTE (RR 1.17, 95% CI 0.87-1.58), though these data do not exclude the potential for a clinically significant increase in the risk for adverse events with EPO therapy. As a longer-term risk, EPO may theoretically increase the risk of tumor progression or recurrence in patients with cancer. However, these concerns are derived from trials in non-surgical oncologic patients utilizing large doses of ESAs for extended periods of time, particularly when targeting normal hemoglobin levels.[36–38] However, the

overwhelming evidence suggests that EPO use has no significant impact on cancer progression or other adverse oncologic outcomes, particularly when used in low-doses and when pre-treatment hemoglobin concentrations do not exceed 12 g/dL.[39,40] To this end, EPO is used extensively for preoperative anemia management in patients undergoing oncologic surgery.[41] Additionally, EPO is now recommended for the treatment of anemia in critical ill adults by the French Society of Anesthesia & Intensive Care Medicine (SFAR).[42]

2.3.2 KNOWN POTENTIAL BENEFITS

Immediate potential benefits of the intervention include reduced development of iatrogenic anemia (e.g. less blood taken for phlebotomy) and higher hemoglobin concentrations. Additionally, reduced volume phlebotomy techniques have shown transfusion reductions.[24] Further, studies consistently link treatment with iron and/or EPO to higher hemoglobin recovery during critical illness. While the impact of iron on RBC transfusions is equivocal in critical illness, EPO reduces RBC transfusion requirements and may reduce mortality.[42]

Regarding intermediate and long-range potential benefits, observational data suggests that patients with higher hemoglobin concentrations may have improved hospital and post-hospitalization outcomes.[7] Recent clinical trial data also suggests that anemia treatment with iron +/- EPO may reduce mortality through 1-year after hospitalization.[43] Further, trial data in surgical patients suggests that anemia treatment with IV iron leads to fewer hospital readmissions.[33] Hence, patients in the intervention arm may potentially benefit by 1) achieving higher hemoglobin levels, 2) experiencing improvement in functional outcomes after hospitalization (physical, cognitive, mental health, quality of life), 3) experiencing fewer hospital readmissions, and 4) experiencing greater long-term survival. All patients will receive the benefit of being formally evaluated by trained study personnel after critical illness. There are no other direct benefits to participation.

2.3.3 ASSESSMENT OF POTENTIAL RISKS AND BENEFITS

There is negligible patient risk associated with optimized phlebotomy and clinical decision support. Despite being used safely in multiple clinical trials in critical illness, there are tangible risks of IV iron and EPO therapies. Risks for pharmacologic anemia treatments (IV iron, EPO) will be minimized in multiple ways. First, we will exclude patients at highest risk for potential harm from these therapies, as outlined in the table below:

Exclusion Criteria	Rationale
Iron (IV) or Erythropoiesis-Stimulating Agents (ESA) use in 30 days prior to admission	Already receiving anemia treatment
Pregnancy or breastfeeding	Unclear safety of EPO
Weight <40 kg	Unable to standardize EPO dosing
Uncontrolled sepsis*	Potential infectious risk with IV iron
Allergy to IV iron or erythropoietin	Risk for allergic reaction
Inability to receive VTE chemoprophylaxis, apart from those with recent bleeding or surgery	Increased thrombosis risk with EPO
Suspected or active thrombosis or myocardial ischemia	Incremental risk with EPO
Uncontrolled hypertension (SBP $\geq$190 or DBP $\geq$110)	Risk for worsening with EPO
Stroke within 3 months prior	Risk for cerebral ischemia with EPO
Mechanical circulatory support devices (e.g. ECMO, ventricular assist device, Impella)	Risk for device thrombosis with EPO

Hb-hemoglobin. VTE-venous thromboembolism; SBP- systolic blood pressure; DBP-diastolic blood pressure.
*Uncontrolled sepsis defined by <48 hours of appropriate antimicrobial therapy and/or lack of definitive source control, given theoretical risk for worsening infection with iron supplementation.[44]

Additionally, pharmacological treatments will be targeted to the underlying etiology of anemia to enhance safety. For example, anemia in acute blood loss and iron deficiency is iron-restricted and likely to respond to iron supplementation alone; hence, the potential benefits of IV iron in this group are high while the potential harms of EPO administration (e.g. thrombosis) likely outweigh any incremental benefit on hemoglobin recovery. Further, IV iron will be given as a single total dose infusion, which provides complete restoration of usable iron for >4 weeks.[45,46] For anemias requiring erythropoietic stimulation, EPO will be given as a single 40,000 unit dose after ensuring restoration of iron stores, a strategy that has resulted in sustained hemoglobin improvement and is widely used in anemia clinics.[46,47] Patients with laboratory evidence (i.e. ferritin > 1000 ng/mL) or clinical conditions (i.e. hemochromatosis) indicating states of iron overload will not receive iron supplementation prior to EPO therapy. A single EPO dose carries a lower risk for thrombotic complications than repeat dosing, which is particularly relevant in the critically ill.

Regarding the safety of IV iron (i.e. low molecular weight iron dextran), this medication is used extensively in modern medical practice. The estimated incidence of serious adverse events (SAEs) is less than 1 in 250,000 administrations.[34] The medication will be administered by critical care nurses who have received training in iron administration in accordance with institutional medication administration protocols. A small test dose of 25 mg will be given over 5 minutes to ensure no symptoms of adverse reaction with continuous assessment by the bedside nurse, including continuous monitoring of pulse oximetry, heart rate, and blood pressure (continuously for those with an invasive arterial catheter, and every 5 minutes for those without) and visual inspection of the patient for rashes or signs of physical and/or respiratory distress. At the discretion of the clinical team, patients deemed to be at high-risk for infusion reactions (e.g. inflammatory arthritis) may be given steroid premedication (e.g. 125 mg methylprednisolone). If a patient develops or is suspected of developing an infusion reaction, therapy will be immediately halted. All infusion reactions will be immediately reported to study personnel for accurate characterization and reporting. Those deemed to have a minor infusion reaction (i.e. rash in absence of hemodynamic or respiratory compromise) will be observed for 15 minutes for signs of clinical progression. If symptoms abate within 15 minutes, the infusion will be restarted at a lower rate. If the patient has persistent mild symptoms, recurrent symptoms, and/or urticaria, they will be treated with an H2-blocker antihistamine (e.g. ranitidine 50 mg), in accordance with institutional policy, prior to restarting the infusion at a lower rate. If there is concern for a moderate infusion reaction or further symptom progression (e.g. hypotension, worsening rash), patients may also receive IV steroids (e.g. methylprednisolone 1-2 mg/kg) +/- a 1L bolus of intravenous isotonic crystalloid (at discretion of ICU team) in addition to H2 blockers. Symptoms should abate completely prior to re-challenging with IV iron, and an alternative iron formulation with comparable dosing should be considered (e.g. iron sucrose). Should a severe reaction be observed (i.e. respiratory distress, anaphylaxis), the patient will receive immediate treatment with IV epinephrine (e.g. 0.1 mg) and additional cardiopulmonary support as dictated by the ICU treatment team. Patients without any apparent reaction to IV iron will be observed clinically for 1 hour post-infusion for the development of delayed reactions with blood pressure measurements at least every 15 minutes, continuous pulse oximetry, and telemetry. Those experiencing infusion reactions will be observed for longer times as dictated by the severity of the reaction. It is commonly thought that iron may predispose patients to bacterial infections;[44] however, this has not been shown in multiple clinical trials in the critically ill.[32,48,49] Out of an abundance of caution, we will exclude patients with uncontrolled sepsis, defined as <48 hours of appropriate antimicrobial therapy and/or lack of definitive source control.

Regarding EPO, several actions are being taken to mitigate risk: 1) excluding patients with relative contraindications to EPO therapy (e.g. pregnancy, active thrombosis, an inability to receive VTE pharmacoprophylaxis, active myocardial ischemia, poorly controlled hypertension, recent stroke); 2) tailoring pharmacotherapy to anemia etiology, such that EPO is not administered to patients that are likely to mount an appropriate erythropoietic response with iron therapy alone; and 3) providing only a single dose of EPO therapy for those receiving this therapy. Of note, the optimal dosing of EPO is unknown in the critically ill, though clinical trials in surgical patients have shown that a single dose of EPO results in sustained hemoglobin improvement.[47] All adverse events will be closely evaluated by study personnel as outlined in the data safety monitoring plan. Similar to iron, EPO will be administered by critical care nurses in accordance with institutional medical administration protocols. Iron will be co-administered immediately prior to EPO to ensure adequate iron stores for EPO-induced augmentation of bone marrow erythropoiesis. Patients will be continuously

monitored at the time of administration for immediate adverse effects (e.g. cutaneous reactions, hypertension, dizziness, nausea). Those without immediate reaction will be observed clinically for 1 hour post-infusion for the development of delayed reactions with blood pressure measurements at least every 15 minutes, continuous pulse oximetry, and telemetry. Those experiencing infusion reactions will be observed for longer times as dictated by the severity of the reaction.

Confidentiality of all participants in the proposed research will be fully protected. Participant privacy and confidentiality will be maintained by conducting the proposed activities in accordance with strict institutional guidelines, which require that formal approval be obtained from all appropriate committees before medical records are reviewed or patient contact is initiated. All study records will be kept in a password-protected study folder and/or locked file cabinets. Individual participants are identified in all computer files and analyses only by a unique study number, which bears no relationship to personal identifiers including name, initials, address, telephone number, social security number, or patient number. All study staff will be trained in HIPAA requirements. Moreover, there is intensive orientation on the confidentiality of medical records and protected health information. Data sharing policies include the requirement that all "identifiers" be removed. All data are tracked in databases by anonymous but linkable study numbers. No identifiable information is explicitly released. During hospitalization, patients will continue to receive care as directed by the primary clinical team. Any adverse reactions to iron and/or EPO will be managed by the primary clinical team per unit-specific protocol. Dosing regiments for these medications have been tailored to minimize any potential risks. Additionally, patients at greatest potential for risk will be excluded. There is minimal risk to patients at follow-up assessments; however, if a patient needs medical evaluation, there is a medical emergency response team immediately available (within 5 minutes) to direct cares, which may include emergency department transfer and/or hospital admission.

3 OBJECTIVES AND ENDPOINTS

OBJECTIVES	ENDPOINTS	JUSTIFICATION FOR ENDPOINTS
Primary		
To assess the efficacy of the intervention on mean difference in hemoglobin concentrations at 1 month post-hospitalization	Hemoglobin concentrations [Time frame: 1 month post-hospitalization]	Hemoglobin concentrations are widely available to infer changes in RBC mass from anemia management interventions
Secondary		
To assess the impact of the intervention on hemoglobin concentrations through 3-months post hospitalization	Hemoglobin concentrations [Time frame: through 3 months post-hospitalization]	Hemoglobin concentrations are widely available to infer changes in RBC mass from anemia management interventions
To assess the impact of the intervention on phlebotomy draws and volumes throughout hospitalization	Phlebotomy draws and volumes [Time frame: through hospitalization]	Phlebotomy draws and volumes are utilized to assess the efficacy of optimized phlebotomy and clinical decision support
To assess the impact of the intervention on quality of life after critical illness	EQ-5D [Time frame: hospital discharge, 1 month, and 3 months post-hospitalization]	Quality of life patient-reported outcome recommended as part of Core Outcome Measurement Set (COMS) for ICU survivors
To assess the impact of the intervention on fatigue after critical illness	PROMIS-Fatigue [Time frame: hospital discharge, 1 month, and 3 months post-hospitalization]	Anemia-related fatigue patient-reported outcome

OBJECTIVES	ENDPOINTS	JUSTIFICATION FOR ENDPOINTS
To assess the impact of the intervention on physical function after critical illness	6-minute walk distance, activities of daily living survey [Time frame: 1 and 3-months post-hospitalization]	Recommended as part of Core Outcome Measurement Set (COMS) for ICU survivors
To assess the impact of the intervention on cognitive function after critical illness	MoCA-BLIND [Time frame: 1 and 3-months post-hospitalization]	Recommended as part of Core Outcome Measurement Set (COMS) for ICU survivors
To assess the impact of the intervention on mental health after critical illness	HADS, IES-R [Time frame: 1 and 3-months post-hospitalization]	Recommended as part of Core Outcome Measurement Set (COMS) for ICU survivors
To assess the impact of the intervention on RBC transfusions	Allogeneic RBC transfusions (units) [Time frame: hospitalization, through 3-months post-hospitalization]	Anemia management may directly result in reductions in RBC transfusions
To assess the impact of the intervention on unplanned hospital readmissions through 12-months post-hospitalization.	Unplanned hospital readmissions [Time frame: 12 months]	Previous data suggests that anemia treatment with iron therapies may reduce hospital readmissions
To assess the impact of the intervention on mortality through 12-months post-hospitalization	All-cause mortality [Time frame: 12 months]	Previous data suggests that IRON and EPO may reduce mortality during and after critical illness

4 STUDY DESIGN

4.1 OVERALL DESIGN

This is a pragmatic, open-label, single-center, randomized phase 2 pilot clinical trial for superiority of a multi-faceted anemia prevention and treatment strategy assessing the impact of the intervention on hemoglobin concentrations and functional outcomes after hospitalization. We hypothesize that the multifaceted anemia intervention will increase hemoglobin concentrations and will improve multi-faceted functional recovery after hospitalization when compared to standard care.

Patients satisfying inclusion/exclusion criteria, or their legal proxies, will be approached by trained study coordinators for written informed consent. Participants will be randomized 1:1 to active intervention vs. standard care using a stratified permuted block design by anemia etiology (iron-responsive or not) and ICU admission indication (surgical vs. non-surgical). The intervention arm is multi-faceted with 3 primary components: 1) **Optimized phlebotomy**, defined by minimal volume draws (i.e. 0.1-2 ml vs. standard 3-10 ml per laboratory order) and closed-loop blood sampling (eliminates 10 ml waste volume per draw), all performed by a dedicated phlebotomy team independent from the treatment team; 2) **Decision support aids**, including visual and electronic alerts reminding the care team to minimize non-essential laboratory testing and mitigate patient-specific bleeding risk (e.g. stress ulcer prophylaxis in high-risk patients); and 3) **Pharmacologic anemia treatment** (given immediately following enrollment when hemoglobin first observed <10 g/dL) targeted to 2 broad groups: 1) <u>anemias responsive to iron supplementation</u> alone (i.e. acute blood loss, true iron deficiency [i.e. ferritin <100 ng/ml, transferrin saturation < 20%]), and 2) <u>anemias requiring erythropoietic stimulation</u> (e.g. anemia of inflammation, anemia of renal disease). Etiology of anemia will be determined immediately prior to randomization upon review of laboratory values (i.e. iron studies) and clinical history (i.e. admission diagnoses, surgery/acute blood loss) with treatment decisions adjudicated pre-randomization by the PI or Co-I's. Subjects then randomized to the intervention arm will receive pharmacologic anemia treatment in accordance with their anemia etiology. Patients with anemias responsive to iron supplementation will receive a single total dose infusion (1000 mg) of intravenous (IV) iron dextran. Patients with anemias requiring erythropoietic stimulation will receive a single dose of 40,000 units of subcutaneous EPO, which will be immediately preceded by 1000 mg of IV iron dextran to replenish

usable iron stores if the ferritin level is < 1000 ng/ml. All patients randomized to the intervention group will receive either IV iron, EPO, or both. Targeted pharmacologic anemia therapies are being employed given that anemia in acute blood loss/post-surgery and true iron deficiency is iron-restricted and responds to iron supplementation alone; hence, the potential harms of EPO (e.g. thrombosis) likely outweigh benefits.

Outcome assessment:

In-person outcome assessments will occur at 1 month and 3 months post-hospitalization, with investigators and outcome assessors blinded to treatment allocation. The **primary outcome** will be the mean difference in hemoglobin between groups at 1 month. Differences in hemoglobin concentrations will also be assessed at ICU discharge, hospital discharge, and 3 month follow-up. **Secondary outcomes** will include changes in phlebotomy practice (i.e. number of draws, total volume of draws) during hospitalization and functional outcomes at 1 and 3 months, employing the NHLBI-funded Core Outcome Measurement Set (COMS) for survivors of critical illness (aim 2b).[23,50] This includes validated measures of physical function (6MWD, ADL survey), cognition (Montreal Cognitive Assessment Blind), mental health (Hospital Anxiety and Depression Scale, Impact of Events Scale-Revised), and quality of life (EQ-5D). We will also assess several Epic-embedded patient-reported outcomes (i.e. PROMIS) with multidimensional computerized adaptive testing (i.e. PROMIS CAT), laying the groundwork for validation in critical illness research. Differences in RBC transfusion rates will be assessed through 3 month follow-up. We will also assess hospital readmissions and mortality through 12-months post-hospitalization. Research samples obtained will be stored for potential biomarker assessment (i.e. hepcidin, IL-6, C-reactive protein) for future mechanistic studies on anemia development and recovery in critical illness survivors.

Sample size estimation:

Power/sample size are calculated to detect a difference in the primary endpoint of hemoglobin at 1 month follow-up. Preliminary data show mean (standard deviation) hemoglobin levels of 10.8 (1.5) g/dL at 1 month among 636 patients with similar inclusion/exclusion criteria receiving standard care. A total sample size of 74 (37 per group) provides 80% power to detect 1.0 g/dL improvement using a two-sample unequal variances t-test with 2-sided alpha of 0.05 to compare 1 month hemoglobin between randomized arms. Actual power is expected to be higher under the analysis approach, adjusting for pre-randomization prognostic variables to reduce residual variation. If adjustment variables account for 25% of the variation in 1 month hemoglobin (R^2 for relationship between pre-randomization adjustment variables and outcome = 0.25), the sample size of 37 per group provides 90% power to detect a 1.0 g/dL improvement using analysis of covariance with 2-sided alpha of 0.05. While the analysis uses a linear mixed-effects model (LMM), power is not appreciably different for the LMM as compared to the unequal variances t-test or ANCOVA described here. Given expected dropout of up to 25% (death, loss to follow-up) resulting in loss of information, 100 subjects will be enrolled.

Statistical considerations:

The primary outcome is hemoglobin measured repeatedly on subjects at ICU discharge, hospital discharge, and 1 month and 3 months. The longitudinal trajectory of hemoglobin will be analyzed with a LMM. The primary parameter of interest is a treatment group by time interaction to estimate the effect of treatment at each follow up time-point. The treatment effect on 1 month hemoglobin is the primary outcome; other time-points will reflect secondary outcomes. As the functional form of hemoglobin over time is unknown, a discrete time representation will be used. The model will adjust for pre-randomization hemoglobin, age, sex, anemia etiology, and medical vs surgical ICU setting to reduce residual variation and improve precision of the estimated treatment effect.

Dropout or non-response including skipped study visit and death represent two forms of missing data. Dropout or non-response unrelated to death at ICU discharge and hospital discharge is expected to be negligible but could occur with withdrawal of consent. We assume dropout (unrelated to death) while in the ICU is missing completely at random (MCAR); patients withdrawing consent prior to observation of hemoglobin outcome at ICU discharge will be excluded

from the analysis. Those dropping out after ICU discharge are assumed missing at random (MAR) with missingness possibly related to adjustment covariates, arm, or prior observed hemoglobin values. Analyses using LMM assume dropout, nonresponse, or skipped visits are MAR.

We anticipate up to 10% ICU mortality (despite exclusion of those not expected to survive hospitalization); such subjects will not have observed hemoglobin outcomes. We also anticipate additional post-ICU discharge mortality. In the primary analysis, we assume missing data due to death is MAR. Those with ICU mortality will have ICU discharge hemoglobin multiply imputed under the MAR assumption. Thereafter, LMMs assume additional missing data at other times are MAR. In secondary approaches to the analysis of hemoglobin, we use a worst-case imputation approach, to impute hemoglobins as the worst possible outcome following death. After imputation, if residuals are not reasonably normally distributed, a generalized linear mixed effects proportional odds model will be used or individual timepoints may be assessed by Wilcoxon rank-sum test without covariate adjustment. A similar approach using proportional odds models or Wilcoxon rank-sum test will apply to functional outcomes which are not expected to satisfy regression assumptions including normality of residuals. Similar approaches assuming MAR and MNAR will be applied to missing functional outcome data.

4.2 SCIENTIFIC RATIONALE FOR STUDY DESIGN

This is a randomized parallel arm clinical trial comparing the multifaceted anemia intervention against control (standard care). The control group will receive standard care without an active placebo for several reasons: 1) the multifaceted nature of the study intervention makes it difficult to design a placebo for each unique intervention element, particularly clinical decision support and optimized phlebotomy); 2) the expenses associated with administering a placebo for pharmacologic agents (i.e. iron, EPO) are considerable and would not be possible through the NIH K-23 funding mechanism; and 3) this is a pilot trial which will inform a larger, multi-center definitive clinical trial in which we would have the resources available for placebo administration of pharmacologic therapies. Superiority will be assessed as the goal is to improve anemia in the critically ill, given that the problem of anemia is extremely prevalent in the ICU, rather than to prove non-inferiority against standard care.

4.3 JUSTIFICATION FOR DOSE

IV iron given as a single total dose infusion of 1000 mg of IV low molecular weight iron dextran, which provides complete restoration of usable iron for >4 weeks and is the most commonly used dose in clinical practice for patients with iron deficiency.[45,46] For anemias requiring erythropoietic stimulation, EPO will be given as a single 40,000 unit dose after ensuring restoration of iron stores, a strategy that has resulted in sustained hemoglobin improvement and is widely used in anemia clinics.[46,47] A single EPO dose carries a lower risk for thrombotic complications than repeat dosing, which is particularly relevant in the critically ill. Defining optimal treatment dosing and duration remains a priority for future research.

4.4 END OF STUDY DEFINITION

A participant is considered to have completed the study if they have completed all phases of the study including the last visit shown in the Schedule of Activities (SoA), Section 1.3. Data on mortality and hospital readmission will be extracted at 12-months post-hospitalization for each patient by review of the electronic medical record.

5 STUDY POPULATION

5.1 INCLUSION CRITERIA

To be eligible to participate in this study, an individual must meet all the following criteria:

1. Provision of signed and dated informed consent form (may be completed by legal proxies for those patients unable to provide consent, i.e. sedation/intubation)
2. Stated willingness to comply with all study procedures and availability for the duration of the study, including follow-up assessments
3. Male or female, age $\geq$ 18 years
4. Current ICU admission at Mayo Clinic Rochester
5. Current ICU duration $\leq$ 7 days
6. Patients embedded in the local or regional Mayo Clinic Health System to facilitate post-hospitalization outcome assessment
7. Moderate-to-severe anemia (i.e. hemoglobin concentration < 10 g/dL) at the time of enrollment, with the hemoglobin concentration assessed no more than 24 hours prior to enrollment. If RBC transfusion has been administered between the qualifying hemoglobin assessment and enrollment, a repeat hemoglobin will be required prior to enrollment to ensure that it remains < 10 g/dL.

5.2 EXCLUSION CRITERIA

An individual who meets any of the following criteria will be excluded from participation in this study:

1. IV iron or any ESA use (i.e. darbepoetin, Aranesp, erythropoietin, Epogen, Procrit, Retacrit) within 30 days of enrollment. Oral iron is permitted (e.g. oral iron supplements, multivitamin with iron)
2. Severe anemia prior to hospitalization (i.e. hemoglobin consistently <9 g/dL in the 90 days prior to admission). If hemoglobin is not available or if hemoglobin is ≥9 g/dL at any time within 90 days prior to enrollment, then this criterion is not met, and the patient may be enrolled
3. Known allergic reactions to IV iron or any EPO agent
4. Inability to complete outcome assessments (i.e. not expected to survive hospitalization, unable to make follow-up appointments, non-ambulatory status preceding hospitalization, dementia or other severe cognitive impairment, visual impairment i.e. blind or legally blind, non-English speaking)
5. Pregnancy or breastfeeding at time of enrollment given unclear safety of EPO
6. Inability to receive pharmacologic venous thromboembolic prophylaxis except in patients with acute blood loss anemia (i.e. recent surgery or gastrointestinal bleeding, extracranial bleeding)
7. Active or suspected thrombosis (i.e. DVT, pulmonary embolism, acute arterial thrombus) within 3 months except in patients with acute blood loss anemia (i.e. recent surgery, gastrointestinal bleeding, extracranial bleeding)
8. Uncontrolled sepsis (i.e. lack of definitive source control and/or <48 hours of appropriate antimicrobial therapy)
9. Having received ≥10 units of allogeneic RBCs in the 48 hours before enrollment
10. Acute coronary syndrome (STEMI or NSTEMI) or ischemic stroke within 3 months except in patients with acute blood loss anemia (i.e. recent surgery, gastrointestinal bleeding, extracranial bleeding)
11. Weight less than 40 kg
12. Concerns with study enrollment expressed by the clinical team

5.3 LIFESTYLE CONSIDERATIONS

Not applicable

5.4 SCREEN FAILURES

Screen failures are defined as participants who consent to participate in the clinical trial but are not subsequently randomly assigned to the study intervention or entered in the study. A minimal set of screen failure information is required to ensure transparent reporting of screen failure participants, to meet the Consolidated Standards of Reporting

Trials (CONSORT) publishing requirements and to respond to queries from regulatory authorities. Minimal information includes demography, screen failure details, and eligibility criteria.

Individuals who do not meet the criteria for participation in this trial (screen failure) because of subsequent hemoglobin assessments which prove exclusionary (i.e. hemoglobin > 10 g/dL) may be rescreened as long as they remain in the ICU with a duration not to exceed 7 days at the time of enrollment. Rescreened participants should be assigned the same participant number as for the initial screening.

5.5 STRATEGIES FOR RECRUITMENT AND RETENTION

Recruitment: Trained study coordinators from the Anesthesia Clinical Research Unit will receive daily electronic alerts notifying them of Mayo Clinic Rochester ICU patients $\geq$ 18 years of age with a most recent hemoglobin value <10 g/dL (infrastructure for these near real-time electronic alerts [2-5 minute delay] are already in place through the ICU DataMart). Patients may be present in any adult ICU. These patients will then be screened through Electronic Health Record (EHR) review by study staff to ascertain eligibility in accordance with inclusion/exclusion criteria. Patients meeting inclusion/exclusion criteria will be approached by study coordinators in their patient room, or by telephone if indicated based on current clinical and/or IRB policies in the setting of COVID-19-related clinical practice/research modifications. Study coordinators will meet with patient or their legal proxies in the case of a patient's inability to directly communicate with study personnel (i.e. deep sedation). The study coordinator will review the research study in detail, explaining the purpose of the study including iron and erythropoietin administration, additional necessary phlebotomy and needed follow up post hospital dismissal. It will be made clear that all participants will receive routine clinical care regardless of whether they 1) agree to participate in the trial, or 2) are randomized to the active treatment arm. The informed consent discussion will occur between the patient and a member of the study team at the patient's bedside during ICU admission as soon as the patient meets eligibility criteria (i.e. within 24 hours). The patient/proxy will be allowed time to have all their questions answered questions answered and assure no exclusions to enrollment are identified. Written informed consent will be obtained by study staff with specific training in this procedure. Signed consent forms will be scanned into the patient's HER and a copy will be provided to the study participant. No information will be used from patients that have not given consent to use their medical information. In the event the LAR consents on behalf of the subject, the study coordinator will make sure and visit with the subject once functional status is reestablished to assure the subject wants to continue with participation. By this time, the subject may/may not have already received study drug, so all they would be affirming is their willingness to continue with the follow up visits/labs/questionnaires.

Retention: A high-level of patient follow-up (>75%) is necessary for success, including in-person evaluations for hemoglobin laboratory draws. To achieve this, we have limited the study to residents residing locally (i.e. patients that receive routine medical cares in Mayo Clinic Rochester or the regional Mayo Clinic Health System), a unique population with a high-level of community engagement in clinical research and a high-level of post-hospitalization medical cares obtained primarily at the Mayo Clinic and its affiliated regional sites. Similar studies of Olmsted County residents, including those with laboratory draws, have achieved >90% retention.[51] Additionally, we are partnering with the Mayo Clinic ICU Rehabilitation Program (MCIRP). The MCIRP is a post-ICU clinic staffed by a physician, advanced ICU care nurse practitioner, ICU pharmacist, and occupational therapist, with the recent addition of a nurse coordinator. In the current care model, survivors of critical illness, identified through real-time electronic data "sniffers", and their family members are approached by MCIRP team members for recruitment prior to hospital discharge. Patients are evaluated within 6 months of hospital discharge with a less than 5% no-show rate for enrolled participants and greater than 90% retention for additional visits. Multi-domain functional outcomes (physical, cognitive, mental health, quality of life) are assessed at each study visit in accordance with the NHLBI-funded improveLTO Core Outcome Measure Set (*http://improvelto.com).* Of note, with COVID-19 precautions, most MCIRP visits have been moved to virtual visitations. We will work with the MCIRP to ensure appropriate follow-up of all enrolled patients. Our goal remains in-person evaluations for all study participants in an outpatient setting. Of note, all core functional outcomes can be assessed over the phone with exception of 6MWD. *Additional* retention in this clinical trial will be facilitated through employment of

published cohort retention tools for longitudinal post-hospitalization critical care outcomes research (*http://improveIto.com)*, which have resulted in >90% cohort retention through 12 months.[21] This includes utilization of a defined cohort retention protocol, careful collection of multiple unique sources of patient contact including proxies, frequent patient engagement, reminder notifications (e.g. reminder phone calls starting 2 weeks before each follow-up appointment and subsequently a phone call 1 week and 1 day before the *appointment)*, remuneration (i.e. $25 per follow-up visit – 1 month and 3 months post-hospitalization), and vouchers for parking (4-hour parking passes, given at each follow-up visit). Recognizing that some patients may die or be lost to contact prior to outcome assessment (~25%), we have increased our study sample size from 74 to 100 participants.

6 STUDY INTERVENTION

6.1 STUDY INTERVENTION(S) ADMINISTRATION

6.1.1 STUDY INTERVENTION DESCRIPTION

The intervention arm is multi-faceted with 3 primary components:

1) **Optimized phlebotomy**, defined by minimal volume draws (i.e. 0.1-2 ml vs. standard 3-10 ml per laboratory order) and closed-loop blood sampling, all performed by a dedicated phlebotomy team independent from the treatment team. This intervention simply requires communication with the phlebotomy team rather than any direct change to patient care.
2) **Decision support**. This includes:
    a. A visual clinical support tool (appendix) urging the clinical team to minimize laboratory assessments and optimize patient bleeding risk. This will be available by the clinical workstations for the ICU team and at the patient's bedside.
    b. A daily rounding checklist (appendix) accompanied by brief daily discussion (2-minutes) between trained study coordinators and the ICU care team during clinical rounds to reiterate the purpose of the study. The checklist will be available at the clinical workstations for the ICU team.
    c. Epic direct messages sent twice per day (targeting day and night providers) with the information from the visual support tool to retitrate the purpose of the study.
3) **Pharmacologic anemia** treatment. These therapies will be given immediately following enrollment and targeted to 2 broad groups:
    a. <u>Anemias responsive to iron supplementation</u> alone. This includes patients with anemia secondary to acute blood loss <u>></u> 500 ml and patients with iron deficiency anemia (i.e. ferritin <100 ng/ml or transferrin saturation < 20%).
        - Patients with iron-responsive anemias will receive a single total dose infusion (1000 mg) of intravenous (IV) low molecular weight iron dextran (INFeD)
        - This medication is diluted in 500 ml of 0.9% saline. A 25 mg test dose (12.5) ml is administered over 5 minutes to ensure no immediate hypersensitivity reaction, followed by the completion of the infusion over 1-2 hours (not to exceed 1000 mg/hr). This medication will be given in accordance with Mayo Clinic administration guidelines.
    b. <u>Anemias requiring erythropoietic stimulation</u>. This includes anemia not secondary to acute blood loss or iron deficiency (e.g. anemia of inflammation, anemia of renal disease). Etiology will be determined immediately *prior to randomization* upon review of laboratory values (i.e. iron studies) and clinical history (i.e. admission diagnoses, surgery/acute blood loss) with treatment decisions adjudicated pre-randomization by the PI and trial Co-I's should the PI be unavailable.
        - Patients without iron-responsive anemias will receive a single dose of 40,000 units of subcutaneous EPO (Retacrit). For those with ferritin less than 1000 ng/ml they will also receive 1000 mg IV iron prior to EPO administration to replenish usable iron, given that iron will

invariably be mobilized secondary to EPO stimulation. EPO will be given in accordance with Mayo Clinic administration guidelines.

6.1.2 DOSING AND ADMINISTRATION

Iron dextran shall be administered intravenously as a single dose of 1000 mg (25 mg test dose followed by 975 mg completion dose). EPO shall be administered as a single dose of 40,000 units subcutaneously. There is no patient-specific dose selection. Doses do not need to be modified in relation to meals.

6.2 PREPARATION/HANDLING/STORAGE/ACCOUNTABILITY

6.2.1 ACQUISITION AND ACCOUNTABILITY

This is a multifaceted intervention. Optimized phlebotomy will be coordinated by direct communication to the phlebotomy team by trained study personnel. Clinical decision support will be similarly coordinated by study coordinators. If a patient is randomized to the intervention arm, study coordinators will contact the research pharmacy regarding the need for IV iron +/- EPO. This will be distributed form the research pharmacy to the patient's bedside followed by administration by the bedside critical care nurse. Study coordinators will be available to ensure appropriate administration. Any unused and untampered agents will be immediately returned to the pharmacy.

6.2.2 FORMULATION, APPEARANCE, PACKAGING, AND LABELING

Low molecular weight iron dextran (INFeD®, AbbVie, Inc.), details from package insert: INFeD (iron dextran injection USP) is a dark brown, slightly viscous sterile liquid complex of ferric hydroxide and dextran for intravenous or intramuscular use. Each mL contains the equivalent of 50 mg of elemental iron (as an iron dextran complex), approximately 0.9% sodium chloride, in water for injection. Sodium hydroxide and/or hydrochloric acid may have been used to adjust pH. The pH of the solution is between 5.2 and 6.5.

EPO (Retacrit®, Hospira, Inc), details from package insert: Epoetin alfa-epbx is a 165-amino acid erythropoiesis-stimulating glycoprotein manufactured by recombinant DNA technology. It has a molecular weight of approximately 30,400 daltons and is produced in Chinese Hamster Ovary (CHO) cell line. The product contains the identical amino acid sequence of isolated natural erythropoietin. RETACRIT (epoetin alfa-epbx) injection for intravenous or subcutaneous administration is a sterile, clear, colorless solution in single-dose vials, formulated with an isotonic sodium chloride/sodium phosphate buffered solution. Each 1 mL single-dose vial of 2,000, 3,000, 4,000, and 10,000 Units of epoetin alfa-epbx contains calcium chloride dihydrate (0.01 mg), glycine (7.5 mg), isoleucine (1 mg), leucine (1 mg), L-glutamic acid (0.25 mg), phenylalanine (0.5 mg), polysorbate 20 (0.1 mg), sodium chloride (2.4 mg), sodium phosphate dibasic anhydrous (4.9 mg), sodium phosphate monobasic monohydrate (1.3 mg), and threonine (0.25 mg), in Water for Injection, USP. Each 1 mL vial of 40,000 Units of epoetin alfa-epbx contains calcium chloride dihydrate (0.01 mg), glycine (7.5 mg), isoleucine (1 mg), leucine (1 mg), L-glutamic acid (0.25 mg), phenylalanine (0.5 mg), polysorbate 20 (0.1 mg), sodium chloride (2.2 mg), sodium phosphate dibasic anhydrous (5.7 mg), Reference ID: 4263015 sodium phosphate monobasic monohydrate (1.5 mg), and threonine (0.25 mg), in Water

6.2.3 PRODUCT STORAGE AND STABILITY

Iron (INFeD®): Store at 20°C to 25°C (68°F to 77°F); excursions permitted to 15°C to 30°C (59°F to 86°F).

EPO (Retacrit®): Store at 36°F to 46°F (2°C to 8°C). Do not freeze. Do not shake. Do not use RETACRIT that has been shaken or frozen. Store RETACRIT vials in the original carton until use to protect from light.

6.2.4 PREPARATION

No preparation (thawing, diluting, mixing, reconstitution) is required.

6.3 MEASURES TO MINIMIZE BIAS: RANDOMIZATION AND BLINDING

Participants will be randomized 1:1 to active intervention vs. standard of care using a stratified permuted block design by anemia etiology (iron responsive vs. not) and ICU admission indication (surgical vs. non-surgical). Randomization will occur electronically using the REDCap randomization module. Randomization lists will be developed by the study statisticians and uploaded directly to REDCap. Study staff involved in patient care are unable to access randomization lists in advance and therefore are unlikely to predict future randomization/assignment. Clinicians and subjects will not be blinded to study interventions. However, all data analysts will be blinded to treatment allocation while the study is ongoing.

6.4 STUDY INTERVENTION COMPLIANCE

Compliance will be assessed by the subject's receipt of pharmacologic therapy (iron +/- EPO). This will be assessed on study day 1 by research personnel. The proportion of subjects successfully receiving the intervention will be reported (number, %). Similarly, the proportion of subjects seen for 1-month and 3-month follow-up assessments will be reported.

6.5 CONCOMITANT THERAPY

For this protocol, a prescription medication is defined as a medication that can be prescribed only by a properly authorized/licensed clinician. Medications to be reported at baseline are all concomitant prescription medications, over-the-counter medications and supplements. Medications to be reported at daily visits are only to include if the subject has received iron or EPO/darbepoetin on these days.

Subjects who received iron therapies (exclusive or multivitamins with iron) or erythropoiesis stimulating agents (i.e. darbepoetin , erythropoietin) within 30 days will be excluded, as these subjects have already been receiving anemia treatment prior to enrollment.

6.5.1 RESCUE MEDICINE

Not applicable

7 STUDY INTERVENTION DISCONTINUATION AND PARTICIPANT DISCONTINUATION/WITHDRAWAL.

7.1 DISCONTINUATION OF STUDY INTERVENTION

Discontinuation from the pharmacologic therapies (i.e. iron, EPO) does not mean discontinuation from the study, and remaining study procedures should be completed as indicated by the study protocol. If a clinically significant finding is identified (including, but not limited to severe allergic reactions during medication administration) after enrollment, the investigator or qualified designee will determine if any change in participant management is needed. Any new clinically relevant finding will be reported as an adverse event (AE).

The data to be collected at the time of study intervention discontinuation will include the following:

- Reason for discontinuation
- Date and time of discontinuation
- Subject-initiated or investigator-initiated discontinuation
- Changes in vital signs or clinical status that prompted discontinuation
- Adverse events

7.2 PARTICIPANT DISCONTINUATION/WITHDRAWAL FROM THE STUDY

Participants are free to withdraw from participation in the study at any time upon request.
An investigator may discontinue or withdraw a participant from the study for the following reasons:

- Significant study intervention non-compliance
- If any clinical adverse event (AE), laboratory abnormality, or other medical condition or situation occurs such that continued participation in the study would not be in the best interest of the participant
- If the participant meets an exclusion criterion (either newly developed or not previously recognized) that precludes further study participation

The reason for participant discontinuation or withdrawal from the study will be recorded on the appropriate Case Report Form (CRF). Subjects who sign the informed consent form and are randomized but do not receive the study intervention may be replaced. Subjects who sign the informed consent form, and are randomized and receive the study intervention, and subsequently withdraw, or are withdrawn or discontinued from the study, will not be replaced.

7.3 LOST TO FOLLOW-UP

A participant will be considered lost to follow-up if they fail to return for 2 scheduled post-hospitalization visits and is unable to be contacted by the study site staff.

The following actions must be taken if a participant fails to return to the clinic for a required study visit:

- The site will attempt to contact the participant and reschedule the missed visit within 1 week and counsel the participant on the importance of maintaining the assigned visit schedule and ascertain if the participant wishes to and/or should continue in the study.
- Before a participant is deemed lost to follow-up, the investigator or designee will make every effort to regain contact with the participant (where possible, 3 telephone calls and, if necessary, a certified letter to the participant’s last known mailing address or local equivalent methods). These contact attempts should be documented in the participant’s medical record or study file.
- Should the participant continue to be unreachable, they will be considered to have withdrawn from the study with a primary reason of lost to follow-up.

8 STUDY ASSESSMENTS AND PROCEDURES

8.1 EFFICACY ASSESSMENTS

Study subjects will undergo the following procedures/evaluations:

- **Baseline assessments**. Medical history, surgical history, demographic features, height, weight, concomitant medication review and cumulative fluid balance, including previous exposure to blood transfusions, ADL survey.

- **Biological specimen collection and laboratory evaluations**. Following enrollment, study labs will be obtained according to table 1.3. CBC and creatinine is often done daily as standard of care. If the subject has a CBC and creatinine value obtained within the prior 24 hrs, this result may be used. However, if they received a unit of blood after that timepoint, the CBC would need to be repeated. All subjects will return for followup assessments and blood draws as noted in table 1.3. The research blood samples for storage and future research will be obtained (10 ml per subject) at enrollment and post-hospitalization assessments for future mechanistic studies. These samples will be cold-stored.
- **Administration of questionnaires or other instruments**. Subjects will undergo functional outcome assessments in accordance with the NHLBI-supported COMS of critical illness. These measures, either comprehensively or in part (as outlined in 1.3), will be obtained at enrollment, hospital discharge, and 1-month and 3-months post-hospitalization.

8.2 SAFETY AND OTHER ASSESSMENTS

Subjects will undergo the following procedures/evaluations:

- **Vital signs**. Temperature, pulse, respirations, blood pressure, continuous telemetry, oxygen saturations will be monitored during administration of iron and EPO for those in the intervention group as outlined in section 2.3. All subjects will receive standard ICU monitoring throughout their stay in the ICU.

- **Weight**. Subjects < 40 kg will be excluded due to unclear safety of 40,000 unit of EPO in persons with low weights

- **Assessment of adverse events.** Medical records will be reviewed daily for evaluation of adverse events. Adverse events will be indicated on the data forms for the study and on the specific adverse event report forms and all serious adverse events will be reported to the IRB within 24 hours of the research team learning about the event followed by a more detailed written report to the IRB. The following information about adverse events will be collected: 1) the onset and resolution of the event, 2) an assessment of the severity or intensity of the event, 3) an assessment of the relationship of the event to the intervention, and 4) any action taken because of event. The PI will report all potentially related SAEs to the DSMB and to NHLBI within 7 days of discovery.

8.3 ADVERSE EVENTS AND SERIOUS ADVERSE EVENTS

8.3.1 DEFINITION OF ADVERSE EVENTS (AE)

Adverse event means any untoward medical occurrence associated with the use of an intervention in humans, whether or not considered intervention-related (21 CFR 312.32 (a)).

As our subject population is by definition ‘critically ill’, it is expected that they will have many unrelated adverse health events during their hospital stay. Therefore, we will limit the scope of our adverse event monitoring and recording to focus on the following conditions:

- Study drug infusion reactions
- Venous thromboembolic disease
- Myocardial infarction
- Non-hemorrhagic stroke
- Bloodstream infections
- Hypertensive urgency or emergency in the 1 hour after study drug administration (i.e. SBP $\geq$ 200 or SBP increase by > 40 mmHg from the time of infusion start)

8.3.2 DEFINITION OF SERIOUS ADVERSE EVENTS (SAE)

An adverse event (AE) or suspected adverse reaction is considered "serious" if, in the view of either the investigator or sponsor, it results in any of the following outcomes: death, a life-threatening adverse event, inpatient hospitalization or prolongation of existing hospitalization, or a persistent or significant incapacity or substantial disruption of the ability to conduct normal life functions.

Serious adverse events (SAEs) will be defined as:

- Death, believed to be related to the study procedures, or a death that is unexpected considering the acuity of a subject.
- A life-threatening experience, including severe infusion reactions, believed to be related to the study procedures.
- Persistent or significant disability or incapacity that is of greater frequency or severity than what would be normally expected in the course of critical illness.
- An event that jeopardizes the human subject and may require medical or surgical treatment to prevent one of the preceding outcomes and is not expected in the subject’s ICU course.

8.3.3 CLASSIFICATION OF AN ADVERSE EVENT

8.3.3.1 SEVERITY OF EVENT

For adverse events (AEs), the following guidelines will be used to describe severity.

- **Mild** – Events require minimal or no treatment and do not interfere with the participant’s clinical course.
- **Moderate** – Events result in a low level of inconvenience or concern with the therapeutic measures. Moderate events may cause some interference with the subject’s clinical course.
- **Severe** – Events interrupt a participant’s clinical course and may require systemic drug therapy or other treatment. Severe events are usually potentially life-threatening or incapacitating.

8.3.3.2 RELATIONSHIP TO STUDY INTERVENTION

All adverse events (AEs) must have their relationship to study intervention assessed by the clinician who examines and evaluates the participant based on temporal relationship and his/her clinical judgment. The degree of certainty about causality will be graded using the categories below. In a clinical trial, the study product must always be suspect.

- **Definitely Related** – There is clear evidence to suggest a causal relationship, and other possible contributing factors can be ruled out. The clinical event occurs in a plausible time relationship to study intervention administration and cannot be explained by concurrent disease or other drugs or chemicals. The response to withdrawal of the study intervention (dechallenge) should be clinically plausible. The event must be pharmacologically or phenomenologically definitive, with use of a satisfactory rechallenge procedure if necessary.
- **Probably Related** – There is evidence to suggest a causal relationship, and the influence of other factors is unlikely. The clinical event occurs within a reasonable time after administration of the study intervention, is unlikely to be attributed to concurrent disease or other drugs or chemicals, and follows a clinically reasonable response on withdrawal (dechallenge). Rechallenge information is not required to fulfill this definition.

- **Potentially Related** – There is some evidence to suggest a causal relationship (e.g., the event occurred within a reasonable time after administration of the trial medication). However, other factors may have contributed to the event (e.g., the participant's clinical condition, other concomitant events). Although an AE may rate only as "possibly related" soon after discovery, it can be flagged as requiring more information and later be upgraded to "probably related" or "definitely related", as appropriate.
- **Unlikely to be related** – A clinical event whose temporal relationship to study intervention administration makes a causal relationship improbable (e.g., the event did not occur within a reasonable time after administration of the study intervention) and in which other drugs or chemicals or underlying disease provides plausible explanations (e.g., the participant's clinical condition, other concomitant treatments).
- **Not Related** – The AE is completely independent of study intervention administration, and/or evidence exists that the event is definitely related to another etiology. There must be an alternative, definitive etiology documented by the clinician.

8.3.3.3 EXPECTEDNESS

The PI will be responsible for determining whether an adverse event (AE) is expected or unexpected. An AE will be considered unexpected if the nature, severity, or frequency of the event is not consistent with the risk information previously described for the study intervention.

8.3.4 TIME PERIOD AND FREQUENCY FOR EVENT ASSESSMENT AND FOLLOW-UP

The occurrence of an adverse event (AE) or serious adverse event (SAE) may come to the attention of study personnel during study visits and interviews of a study participant presenting for medical care, or upon review by a study monitor.

As noted earlier, for this study we plan to observe for a specific set of AEs and this information will be captured on the appropriate case report form (CRF). Information to be collected includes event description, time of onset, clinician's assessment of severity, relationship to study product (assessed only by those with the training and authority to make a diagnosis), and time of resolution/stabilization of the event. All AEs will be followed to adequate resolution.

Changes in the severity of an AE will be documented to allow an assessment of the duration of the event at each level of severity to be performed.

Study coordinators will record all listed reportable events with start dates occurring any time after informed consent is obtained through 3-months after hospitalization. At each study visit, the investigator will inquire about the occurrence of AE/SAEs since the last visit. Events will be followed for outcome information until resolution or stabilization.

8.3.5 ADVERSE EVENT REPORTING

All adverse events will be indicated on the data forms for the study and on the specific adverse event report forms and all serious adverse events will be reported to the IRB within 24 hours of the research team learning about the event followed by a more detailed written report to the IRB. The following information about adverse events will be collected: 1) the onset and resolution of the event, 2) an assessment of the severity or intensity of the event, 3) an assessment of the relationship of the event to the intervention, and 4) any action taken because of event. The PI will report all potentially related SAEs to the NHLBI immediately after discovery (within 7 days).

8.3.6 SERIOUS ADVERSE EVENT REPORTING

The study clinician will immediately report to the IRB any serious adverse event, whether or not considered study intervention related, including those listed in the protocol or investigator brochure and must include an assessment of whether there is a reasonable possibility that the study intervention caused the event. Study endpoints that are serious adverse events (e.g., all-cause mortality) must be reported in accordance with the protocol unless there is evidence suggesting a causal relationship between the study intervention and the event (e.g., death from anaphylaxis). In that case, the investigator must immediately report the event to the sponsor. All serious adverse events (SAEs) will be followed until satisfactory resolution or until the site investigator deems the event to be chronic or the participant is stable.

8.3.7 REPORTING EVENTS TO PARTICIPANTS

Study coordinators will disclose any adverse events that are probably or definitively-related to the enrolled subject after review by the PI.

8.3.8 EVENTS OF SPECIAL INTEREST

Not applicable

8.3.9 REPORTING OF PREGNANCY

Not applicable

8.4 UNANTICIPATED PROBLEMS

8.4.1 DEFINITION OF UNANTICIPATED PROBLEMS (UP)

The Office for Human Research Protections (OHRP) considers unanticipated problems involving risks to participants or others to include, in general, any incident, experience, or outcome that meets **all** the following criteria:

- Unexpected in terms of nature, severity, or frequency given (a) the research procedures that are described in the protocol-related documents, such as the Institutional Review Board (IRB)-approved research protocol and informed consent document; and (b) the characteristics of the participant population being studied;
- Related or possibly related to participation in the research ("possibly related" means there is a reasonable possibility that the incident, experience, or outcome may have been caused by the procedures involved in the research); and
- Suggests that the research places participants or others at a greater risk of harm (including physical, psychological, economic, or social harm) than was previously known or recognized.

8.4.2 UNANTICIPATED PROBLEM REPORTING

The investigator will report unanticipated problems (UPs) to the reviewing Institutional Review Board (IRB) and to the principal investigator (PI). The UP report will include the following information:

- Protocol identifying information: protocol title and number, PI's name, and the IRB project number;
- A detailed description of the event, incident, experience, or outcome;
- An explanation of the basis for determining that the event, incident, experience, or outcome represents an UP;
- A description of any changes to the protocol or other corrective actions that have been taken or are proposed in response to the UP.

To satisfy the requirement for prompt reporting, UPs will be reported using the following timeline:

- UPs that are serious adverse events (SAEs) will be reported to the IRB within 7-days of the investigator becoming aware of the event.
- Any other UP will be reported to the IRB and to the DCC/study sponsor within 10-days of the investigator becoming aware of the problem.
- All UPs should be reported to appropriate institutional officials (as required by an institution's written reporting procedures), the supporting agency head (or designee), and the Office for Human Research Protections (OHRP) within 10 days of the IRB's receipt of the report of the problem from the investigator.

8.4.3 REPORTING UNANTICIPATED PROBLEMS TO PARTICIPANTS

Study coordinators will disclose UPs that are probably or definitively-related to the enrolled subject.

9 STATISTICAL CONSIDERATIONS

9.1 STATISTICAL HYPOTHESES

In this study, we will perform a pilot pragmatic clinical trial testing a multi-faceted anemia intervention (optimized phlebotomy practice, decision support, targeted pharmacologic anemia treatment) to attenuate anemia development and promote functional recovery in the setting of critical illness

Primary endpoint: To assess the impact of the intervention on 1 month post-hospitalization hemoglobin concentrations.

Secondary endpoint: To assess the impact of the intervention on post-hospitalization functional outcomes and hemoglobin concentrations measured at 3 months.

<u>Hypotheses</u>: A multifaceted anemia intervention will increase hemoglobin concentrations and will improve functional recovery through 3 months after hospitalization.

9.2 SAMPLE SIZE DETERMINATION

Power/sample size are calculated to detect a difference in the primary endpoint of hemoglobin at 1 month follow-up. Preliminary data show mean (standard deviation) hemoglobin levels of 10.8 (1.5) g/dL at 1 month among 636 subjects with similar inclusion/exclusion criteria receiving standard care. A total sample size of 74 (37 per group) provides 80% power to detect 1.0 g/dL improvement using a two-sample unequal variances t-test with 2-sided alpha of 0.05 to compare 1 month hemoglobin between randomized arms. Actual power is expected to be higher under the analysis approach, adjusting for pre-randomization prognostic variables to reduce residual variation. If adjustment variables account for 25% of the variation in 1 month hemoglobin (R^2 for relationship between pre-randomization adjustment variables and outcome = 0.25), the sample size of 37 per group provides 90% power to detect a 1.0 g/dL improvement using analysis of covariance with 2-sided alpha of 0.05. While the analysis uses a linear mixed-effects model (LMM), power is not appreciably different for the LMM as compared to the unequal variances t-test or ANCOVA described here. Given expected dropout of up to 25% (death, loss to follow-up) resulting in loss of information, 100 subjects will be enrolled.

The study has not been powered for exploratory outcomes such as RBC transfusion, mortality, and readmissions, but this data will serve for hypothesis-generation for future, larger clinical trials.

9.3 POPULATIONS FOR ANALYSES

As the primary approach, subjects will be analyzed using an intention-to-treat (ITT) approach. Secondarily, analysis will be performed using modified intention-to-treat.

9.4 STATISTICAL ANALYSES

9.4.1 GENERAL APPROACH

Baseline demographic and clinical characteristics will be presented using descriptive statistics, as applicable. Unadjusted outcome summary statistics will be described for each randomized group. Continuous variables will be summarized by mean (standard deviation), median (25th, 75th) percentiles, and range. Categorical variables will be summarized by percentage.

The primary analysis dataset consists of all randomized participants and will be analyzed using intention-to-treat (ITT) principles such that each subject is analyzed based on their allocated arm. Mortality and dropout may occur, and subjects will be analyzed as described in 9.4.2 and 9.4.3.

A secondary analysis approach may consider a per-protocol analysis, analyzing those participants who completed study procedures without major protocol violations. The per-protocol analysis definition will be finalized prior to database lock and data analysis. The ITT analysis will be considered primary.

9.4.2 ANALYSIS OF THE PRIMARY EFFICACY ENDPOINT(S)

The primary outcome is hemoglobin measured repeatedly on subjects at ICU discharge, hospital discharge, and 1 month and 3 months. The longitudinal trajectory of hemoglobin will be analyzed with a LMM. The primary parameter of interest is a treatment group by time interaction to estimate the effect of treatment at each follow up time-point. The treatment effect on 1 month hemoglobin is the primary outcome; other time-points will reflect secondary outcomes. As the functional form of hemoglobin over time is unknown, a discrete time representation will be used. The model will adjust for pre-randomization hemoglobin, age, sex, anemia etiology, and medical vs surgical ICU setting to reduce residual variation and improve precision of the estimated treatment effect.

Dropout or non-response including skipped study visit and death represent two forms of missing data. Dropout or non-response unrelated to death at ICU discharge and hospital discharge is expected to be negligible but could occur with withdrawal of consent. We assume dropout (unrelated to death) while in the ICU is missing completely at random (MCAR); subjects withdrawing consent prior to observation of hemoglobin outcome at ICU discharge will be excluded from the analysis. Those dropping out after ICU discharge are assumed missing at random (MAR) with missingness possibly related to adjustment covariates, arm, or prior observed hemoglobin values. Analyses using LMM assume dropout, nonresponse, or skipped visits are MAR.

We anticipate up to 10% ICU mortality (despite exclusion of those not expected to survive hospitalization); such subjects will not have observed hemoglobin outcomes. We also anticipate additional post-ICU discharge mortality. In the primary analysis, we assume missing data due to death is MAR. Those with ICU mortality will have ICU discharge hemoglobin multiply imputed under the MAR assumption. Thereafter, LMMs assume additional missing data at other times are MAR. In secondary approaches to the analysis of hemoglobin, we use a worst-case imputation approach, to impute hemoglobins as the worst possible outcome following death. After imputation, if residuals are not reasonably normally distributed, a generalized linear mixed effects proportional odds model will be used or individual timepoints may be assessed by Wilcoxon rank-sum test without covariate adjustment.

In the primary analysis, the point estimate, 95% confidence interval, and p-value will be reported from the LMM for the 1 month hemoglobin comparison. A point estimate in the direction favoring higher hemoglobin for the intervention arm

and two-sided p-value<0.05 will reject the null hypothesis of no treatment benefit in favor of the conclusion that intervention improves 1 month hemoglobin.

9.4.3 ANALYSIS OF THE SECONDARY ENDPOINT(S)

Secondary and exploratory endpoints (besides hemoglobin at 3 months) include EQ-5D [Time frame: hospital discharge, 1 month, and 3 months post-hospitalization], PROMIS-Fatigue [Time frame: hospital discharge, 1 month, and 3 months post-hospitalization], 6-minute walk distance, activity monitoring (i.e. daily step counts, daily energy expenditure), and activities of daily living survey [Time frame: 1 and 3-months post-hospitalization], MoCA-BLIND [Time frame: 1 and 3-months post-hospitalization], HADS and IES-R [Time frame: 1 and 3-months post-hospitalization].

Analyses will use Wilcoxon rank-sum tests to compare the distribution of each outcome between groups at each timepoint since assumptions including normality of residuals are not expected to be satisfied for these outcomes. Additional, when applicable, a cutpoint defining a clinically actionable adverse outcome may be defined, for example, defining depression by HADS-Depression ≥8. Binary outcomes will be summarized as proportion and compared by randomized arm using a Chi-square test. Death and dropout will be assumed MAR in the primary approach and multiple imputation will be used to impute missing values. Analyses will be conducted on each imputed dataset and results combined to reflect uncertainty due to missing data. An additional analysis will consider a worst-value imputation for mortality, assigning death the worst possible outcome.

Mortality and readmission through 1 year post-discharge will be described by randomized arm using cumulative incidence estimates, censoring subjects at last known contact with the Mayo medical system when status is unknown at 1 year. Inferential analyses will use log-rank tests and Gray's test for mortality and readmission outcomes, respectively.

Since the goal is to provide robust data for further clinical trial evaluation, secondary and exploratory outcomes will be assessed without adjustment for multiplicity, with conclusions from each based on a two-sided alpha level 0.05 statistical test.

9.4.4 SAFETY ANALYSES

All AEs and SAEs will be presented and compared between groups. Mortality and hospital readmission rates will also be presented and compared between groups.

9.4.5 BASELINE DESCRIPTIVE STATISTICS

Descriptive statistics will be utilized to present baseline demographic, clinical, and laboratory features for both groups. No inferential statistics will be utilized.

9.4.6 PLANNED INTERIM ANALYSES

Not applicable

9.4.7 SUB-GROUP ANALYSES

We do not expect treatment effect heterogeneity of this intervention. However, exploratory analyses will assess potential for heterogeneity of treatment effect using interaction analyses in LMM models for the primary endpoint. An interaction term between randomized arm and each of sex, age, anemic etiology, and surgical vs. medical admissions,

will be evaluated separately. The estimate of the treatment effect will be reported in subgroups using linear contrasts of with the interaction analysis when evidence supports a statistically significant interaction.

9.4.8 TABULATION OF INDIVIDUAL PARTICIPANT DATA

Not applicable.

9.4.9 EXPLORATORY ANALYSES

Multiple exploratory analyses have been planned as outlined throughout the protocol. These will be utilized to inform a definitive multicenter phase III clinical trial.

10 SUPPORTING DOCUMENTATION AND OPERATIONAL CONSIDERATIONS

10.1 REGULATORY, ETHICAL, AND STUDY OVERSIGHT CONSIDERATIONS

10.1.1 INFORMED CONSENT PROCESS

10.1.1.1 CONSENT/ASSENT AND OTHER INFORMATIONAL DOCUMENTS PROVIDED TO PARTICIPANTS

Consent forms describing in detail the study intervention, study procedures, and risks are given to the participant and written documentation of informed consent is required prior to starting intervention/administering study intervention. The following consent materials are submitted with this protocol: Consent form.

10.1.1.2 CONSENT PROCEDURES AND DOCUMENTATION

Informed consent is a process that is initiated prior to the individual's agreeing to participate in the study and continues throughout the individual's study participation. Consent forms will be Institutional Review Board (IRB)-approved and the participant/LAR will be asked to read and review the document. The study coordinator will explain the research study to the participant &/or LAR and answer any questions that may arise. A verbal explanation will be provided in terms suited to the participant's comprehension of the purposes, procedures, and potential risks of the study and of their rights as research participants. Participants/LAR will have the opportunity to carefully review the written consent form and ask questions prior to signing. The participants should have the opportunity to discuss the study with their family or surrogates or think about it prior to agreeing to participate. The participant/LAR will sign the informed consent document prior to any procedures being done specifically for the study. Participants must be informed that participation is voluntary and that they may withdraw from the study at any time, without prejudice. A copy of the informed consent document will be given to the participants for their records. The informed consent process will be conducted and documented in the source document (including the date), and the form signed, before the participant undergoes any study-specific procedures. The rights and welfare of the participants will be protected by emphasizing to them that the quality of their medical care will not be adversely affected if they decline to participate in this study.

10.1.2 STUDY DISCONTINUATION AND CLOSURE

This study may be temporarily suspended or prematurely terminated if there is sufficient reasonable cause. Written notification, documenting the reason for study suspension or termination, will be provided by the suspending or terminating party to study participants, investigator, funding agency, and regulatory authorities. If the study is prematurely terminated or suspended, the PI will promptly inform study participants, IRB, and sponsor and will provide

the reason(s) for the termination or suspension. Study participants will be contacted, as applicable, and be informed of changes to study visit schedule.

Circumstances that may warrant termination or suspension include, but are not limited to:

- Determination of unexpected, significant, or unacceptable risk to participants
- Demonstration of efficacy that would warrant stopping
- Insufficient compliance to protocol requirements
- Data that are not sufficiently complete and/or evaluable
- Determination that the primary endpoint has been met
- Determination of futility

Study may resume once concerns about safety, protocol compliance, and data quality are addressed, and satisfy the sponsor, IRB and/or Food and Drug Administration (FDA).

10.1.3 CONFIDENTIALITY AND PRIVACY

Participant confidentiality and privacy are strictly held in trust by the participating investigators, their staff, and the sponsor(s) and their interventions. This confidentiality is extended to cover testing of biological samples and genetic tests in addition to the clinical information relating to participants. Therefore, the study protocol, documentation, data, and all other information generated will be held in strict confidence. No information concerning the study or the data will be released to any unauthorized third party without prior written approval of the sponsor.

All research activities will be conducted in as private a setting as possible.

The study monitor, other authorized representatives of the sponsor, representatives of the IRB, regulatory agencies or pharmaceutical company supplying study product may inspect all documents and records required to be maintained by the investigator, including but not limited to, medical records (office, clinic, or hospital) and pharmacy records for the participants in this study. The clinical study site will permit access to such records.

The study participant’s contact information will be securely stored at each clinical site for internal use during the study. At the end of the study, all records will continue to be kept in a secure location for as long a period as dictated by the reviewing IRB, Institutional policies, or sponsor requirements.

Study participant research data, which is for purposes of statistical analysis and scientific reporting, will be transmitted to and stored at the Mayo Clinic. This will not include the participant’s contact or identifying information. Rather, individual participants and their research data will be identified by a unique study identification number. The study data entry and study management systems will be secured, and password protected. At the end of the study, all study databases will be de-identified and archived at the Mayo Clinic.

<u>Certificate of Confidentiality</u>

To further protect the privacy of study participants, a Certificate of Confidentiality will be issued by the National Institutes of Health (NIH). This certificate protects identifiable research information from forced disclosure. It allows the investigator and others who have access to research records to refuse to disclose identifying information on research participation in any civil, criminal, administrative, legislative, or other proceeding, whether at the federal, state, or local level. By protecting researchers and institutions from being compelled to disclose information that would identify research participants, Certificates of Confidentiality help achieve the research objectives and promote participation in studies by helping assure confidentiality and privacy to participants.

10.1.4 FUTURE USE OF STORED SPECIMENS AND DATA

With the participant's approval and as approved by local IRBs, de-identified biological samples will be stored at the Mayo Clinic with the same goal as the sharing of data with the Mayo Clinic. These samples could be used to research the causes of anemia, its complications and other conditions for which individuals with anemia are at increased risk, and to improve treatment. The PI and study team will keep a record that will allow linking the biological specimens with the phenotypic data from each participant, maintaining the blinding of the identity of the participant.

During the conduct of the study, an individual participant can choose to withdraw consent to have biological specimens stored for future research. However, withdrawal of consent with regard to biosample storage may not be possible after the study is completed.

When the study is completed, access to study data and/or samples will be provided through the PI and Mayo Clinic.

10.1.5 KEY ROLES AND STUDY GOVERNANCE

**Principal Investigator**
Matthew A. Warner, MD, Associate Professor of Anesthesiology
Mayo Clinic
200 1st St SW, Rochester, MN 55905
507-284-2511, #36558
warner.matthew@mayo.edu

10.1.6 SAFETY OVERSIGHT

Safety oversight will be conducted in accordance with the Data and Safety Monitoring Plan (DSMP).

10.1.7 CLINICAL MONITORING

Clinical site monitoring is conducted to ensure that the rights and well-being of trial participants are protected, that the reported trial data are accurate, complete, and verifiable, and that the conduct of the trial is in compliance with the currently approved protocol/amendment(s), with International Conference on Harmonisation Good Clinical Practice (ICH GCP), and with applicable regulatory requirement(s).

It is the responsibility of the Principal Investigator to oversee the safety of the study. This safety monitoring will include careful assessment and appropriate reporting of adverse events as noted above, as well as the construction and implementation of a site data and safety-monitoring plan. Medical monitoring will include a regular assessment of the number and type of serious adverse events

10.1.8 QUALITY ASSURANCE AND QUALITY CONTROL

The clinical site will perform internal quality management of study conduct, data and biological specimen collection, documentation and completion. An individualized quality management plan will be developed to describe a site's quality management.

Quality control (QC) procedures will be implemented beginning with the data entry system and data QC checks that will be run on the database will be generated. Any missing data or data anomalies will be communicated to the site(s) for clarification/resolution.

Following written Standard Operating Procedures (SOPs), the monitors will verify that the clinical trial is conducted and data are generated and biological specimens are collected, documented (recorded), and reported in compliance with the protocol, International Conference on Harmonisation Good Clinical Practice (ICH GCP), and applicable regulatory requirements (e.g., Good Laboratory Practices (GLP), Good Manufacturing Practices (GMP)).

The investigational site will provide direct access to all trial related sites, source data/documents, and reports for the purpose of monitoring and auditing by the sponsor, and inspection by local and regulatory authorities.

10.1.9 DATA HANDLING AND RECORD KEEPING

10.1.9.1 DATA COLLECTION AND MANAGEMENT RESPONSIBILITIES

Data collection is the responsibility of the clinical trial staff at the site under the supervision of the site investigator. The investigator is responsible for ensuring the accuracy, completeness, legibility, and timeliness of the data reported. All source documents should be completed in a neat, legible manner to ensure accurate interpretation of data. Hardcopies of the study visit worksheets will be provided for use as source document worksheets for recording data for each participant enrolled in the study. Data collected and entered into electronic case report form (eCRF) derived from source documents should be consistent with the data recorded on the source documents. Clinical data (including adverse events (AEs), concomitant medications, and expected adverse reactions data) and clinical laboratory data will be entered into REDCap electronic data capture. REDCap is HIPAA-compliant with built-in user right controls and audit trails for data security and tracking. The data system includes password protection and internal quality checks, such as automatic range checks, to identify data that appear inconsistent, incomplete, or inaccurate. Clinical data will be entered directly from the source documents.

10.1.9.2 STUDY RECORDS RETENTION

Study documents should be retained for a minimum of 2 years. These documents should be retained for a longer period, however, if required by local regulations. No records will be destroyed without the written consent of the sponsor, if applicable. It is the responsibility of the sponsor to inform the investigator when these documents no longer need to be retained.

10.1.10 PROTOCOL DEVIATIONS

A protocol deviation is any noncompliance with the clinical trial protocol, International Conference on Harmonisation Good Clinical Practice (ICH GCP), or Manual of Procedures (MOP) requirements. The noncompliance may be either on the part of the participant, the investigator, or the study site staff. As a result of deviations, corrective actions are to be developed by the site and implemented promptly.

These practices are consistent with ICH GCP:

- 4.5 Compliance with Protocol, sections 4.5.1, 4.5.2, and 4.5.3
- 5.1 Quality Assurance and Quality Control, section 5.1.1
- 5.20 Noncompliance, sections 5.20.1, and 5.20.2.

It is the responsibility of the site investigator to use continuous vigilance to identify and report deviations. All deviations must be addressed in study source documents, reported to the NHLBI Program Official. Protocol deviations must be sent to the reviewing Institutional Review Board (IRB) per their policies. The site investigator is responsible for knowing and adhering to the reviewing IRB requirements.

10.1.11 PUBLICATION AND DATA SHARING POLICY

This study will be conducted in accordance with the following publication and data sharing policies and regulations:

National Institutes of Health (NIH) Public Access Policy, which ensures that the public has access to the published results of NIH funded research. It requires scientists to submit final peer-reviewed journal manuscripts that arise from NIH funds to the digital archive PubMed Central upon acceptance for publication.

This study will comply with the NIH Data Sharing Policy and Policy on the Dissemination of NIH-Funded Clinical Trial Information and the Clinical Trials Registration and Results Information Submission rule. As such, this trial will be registered at ClinicalTrials.gov, and results information from this trial will be submitted to ClinicalTrials.gov. In addition, every attempt will be made to publish results in peer-reviewed journals. Data from this study may be requested from other researchers within 5 years after the completion of the primary endpoint by contacting the PI.

10.1.12 CONFLICT OF INTEREST POLICY

The independence of this study from any actual or perceived influence, such as by the pharmaceutical industry, is critical. Therefore, any actual conflict of interest of persons who have a role in the design, conduct, analysis, publication, or any aspect of this trial will be disclosed and managed. Furthermore, persons who have a perceived conflict of interest will be required to have such conflicts managed in a way that is appropriate to their participation in the design and conduct of this trial. The study leadership in conjunction with NHLBI has established policies and procedures for all study group members to disclose all conflicts of interest and will establish a mechanism for the management of all reported dualities of interest.

10.2 ADDITIONAL CONSIDERATIONS

Appendix 1: Visual Aid

Anemia Study Participant

Anemia is common in ICU patients and is associated with adverse outcomes during and after hospitalization. The goal of this clinical trial is to mitigate anemia severity and treat anemia in critically ill patients. ________________ is enrolled in the active arm of this study, which consists of:

1. *Optimized Phlebotomy*: micro blood draws, closed loop blood sampling, lab bundling
2. *Pharmacological Therapy*: IV iron +/- EPO (single dose at enrollment)
3. *Clinician Engagement & Decision Support*: visual aid, daily communications (Epic, in-person)

What are we asking from you? Continue to provide routine care for patients not in the active study arm (i.e. do **NOT** adjust practice). For patients in the active arm:

- **Remove invasive lines as soon as possible**
  - Arterial and central venous catheters should be removed when no longer required.
  - Do NOT draw cultures from the line that is being removed.
- **Eliminate/minimize all non-essential laboratory draws**
  - Do NOT schedule daily labs. Assess needs daily.
  - Avoid ABGs to assess PaO2 (SpO2 usually sufficient) or after modifying ventilator settings.
  - Avoid ABGs to assess PaCO2 in patients with clear mentation.
  - Avoid serial lactates in patients that are improving.
  - Avoid frequent sodium checks for hyponatremia in absence of hypertonic saline therapy.
  - Avoid serial coagulation tests as postoperative routine (e.g. Q6H INR, APTT, fibrinogen).
- **Consider lab holidays**
  - No daily CBC unless concern for bleeding or new/worsening infection.
  - No daily electrolytes unless requiring active management (e.g. large-volume diuresis).
- **Bundle labs to prevent multiple phlebotomy episodes**
  - If new lab required, order as "add-on".
  - If "add-on" not possible, please schedule the lab to be drawn when others will be drawn.
- **Avoid blood cultures in those with low pre-test probability of bacteremia**
  - Isolated fever and/or leukocytosis
  - Postop fever < 48 hours from surgery
  - Non-severe infections (i.e. CAP, HAP, cellulitis, cystitis)
- **When blood cultures are indicated, do NOT culture all lines**
  - Only culture from one line + one peripheral stick (2 total cultures)
- **Ensure adequate nutrition**
  - Adequate nutrition is important for erythropoiesis
  - Enteral tubes (NG) may cause mucosal erosion and bleeding; remove when no longer required

Appendix 2: Daily Rounding

Daily rounding checklist

- ☐ *Assess suitability for removal of invasive lines* (arterial line, central line)

- ☐ *Review all scheduled laboratory orders*
  - ☐ Does this patient *really* need labs tomorrow? Consider lab holiday
  - ☐ Ensure essential labs are being drawn together (align scheduling)
  - ☐ Avoid routine scheduling of labs (i.e. daily CBC, electrolytes)

- ☐ *Review the necessity for additional laboratory orders*
  - ☐ Does this patient *really* need blood cultures?
    Do NOT order reflexively for those with low pre-test probability of bacteremia (postop fever < 48 hours from surgery, isolated fever and/or leukocytosis, non-severe infections).
    Do NOT cultures every line. Only culture from 1 invasive line + 1 peripheral stick.
  - ☐ Does this patient *really* need an ABG?
    Do I really need to trend the PaO2? Can I simply assess oxygenation with the SpO2?
    If the patient is lucid/stable, do I really care what the PaCO2 is?
  - ☐ Do I need to repeat the lactate if the patient is improving?

- ☐ *Discontinue unnecessary intravenous fluids*
  Excessive IV fluids cause dilutional anemia (i.e. drop in hemoglobin concentration without any change in the actual red cell mass). Maintenance fluids discouraged for patients able to tolerate oral intake.

- ☐ *Ensure adequate nutrition*
  - ☐ Assess suitability for oral diet / dietary advancement
  - ☐ Assess suitability for removal of enteral lines (NG tube)

- ☐ *Ensure appropriate GI stress ulcer prophylaxis*
  (MV > 48 hours, coagulopathy, recent history of GI bleeding, TBI or traumatic SC injury, severe burns; at least 2 of the following: high dose steroids, occult bleeding ≥ 6 days, ICU stay ≥ 7 days, sepsis).

10.3 ABBREVIATIONS

ADL	Activities of Daily Living
AE	Adverse Event
CFR	Code of Federal Regulations
CLIA	Clinical Laboratory Improvement Amendments
CMP	Clinical Monitoring Plan
COC	Certificate of Confidentiality
COMS	Core Outcome Measurement Set
CONSORT	Consolidated Standards of Reporting Trials
CRF	Case Report Form
DCC	Data Coordinating Center
DHHS	Department of Health and Human Services
DSMP	Data Safety Monitoring Plan
DRE	Disease-Related Event
EC	Ethics Committee
eCRF	Electronic Case Report Forms
EHR	Electronic health record
EPO	Erythropoietin
ESA	Erythropoiesis-stimulating agents
FDA	Food and Drug Administration
FDAAA	Food and Drug Administration Amendments Act of 2007
FFR	Federal Financial Report
GCP	Good Clinical Practice
GLP	Good Laboratory Practices
GMP	Good Manufacturing Practices
HIPAA	Health Insurance Portability and Accountability Act
IB	Investigator's Brochure
ICH	International Conference on Harmonisation
ICMJE	International Committee of Medical Journal Editors
IDE	Investigational Device Exemption
IND	Investigational New Drug Application
IRB	Institutional Review Board
ISM	Independent Safety Monitor
ISO	International Organization for Standardization
ITT	Intention-To-Treat
IV	Intravenous
MedDRA	Medical Dictionary for Regulatory Activities
MOP	Manual of Procedures
MSDS	Material Safety Data Sheet
NCT	National Clinical Trial
NIH	National Institutes of Health
NIH IC	NIH Institute or Center
OHRP	Office for Human Research Protections
PI	Principal Investigator
QA	Quality Assurance
QC	Quality Control
SAE	Serious Adverse Event
SAP	Statistical Analysis Plan
SMC	Safety Monitoring Committee
SOA	Schedule of Activities
SOC	System Organ Class

SOP	Standard Operating Procedure
UP	Unanticipated Problem
US	United States
6MWD	6-minute walk distance

11 REFERENCES

1. Walsh TS, Saleh E-E-D. Anaemia during critical illness. *Br J Anaesth*. 2006;97(3):278-291. doi:10.1093/bja/ael189
2. Thomas J, Jensen L, Nahirniak S, Gibney RTN. Anemia and blood transfusion practices in the critically ill: A prospective cohort review. *Hear Lung J Acute Crit Care*. 2010;39(3):217-225. doi:10.1016/j.hrtlng.2009.07.002
3. Warner MA, Hanson AC, Frank RD, et al. Prevalence and Recovery from Anemia Following Hospitalization for Critical Illness among Adults in Minnesota, 2010-2016. *JAMA Netw Open*. 2020;[Accepted.
4. Hébert PC, Wells G, Blajchman MA, et al. A Multicenter, Randomized, Controlled Clinical Trial of Transfusion Requirements in Critical Care. *N Engl J Med*. 1999;340(6):409-417. doi:10.1056/NEJM199902113400601
5. Needham DM, Davidson J, Cohen H, et al. Improving long-term outcomes after discharge from intensive care unit. *Crit Care Med*. 2012;40(2):502-509. doi:10.1097/CCM.0b013e318232da75
6. Wu Z, McGoogan JM. Characteristics of and Important Lessons from the Coronavirus Disease 2019 (COVID-19) Outbreak in China: Summary of a Report of 72314 Cases from the Chinese Center for Disease Control and Prevention. *JAMA - J Am Med Assoc*. 2020;323(13):1239-1242. doi:10.1001/jama.2020.2648
7. Warner MA, Kor DJ, Frank RD, et al. Anemia in Critically Ill Patients With Acute Respiratory Distress Syndrome and Posthospitalization Physical Outcomes. *J Intensive Care Med*. 1(9). doi:10.1177/0885066620913262
8. Den Elzen WPJ, Willems JM, Westendorp RGJ, De Craen AJM, Assendelft WJJ, Gussekloo J. Effect of anemia and comorbidity on functional status and mortality in old age: Results from the Leiden 85-plus Study. *Cmaj*. 2009;181(3-4):151-157. doi:10.1503/cmaj.090040
9. Denny SD, Kuchibhatla MN, Cohen HJ. Impact of anemia on mortality, cognition, and function in community-dwelling elderly. *Am J Med*. 2006;119(4):327-334. doi:10.1016/j.amjmed.2005.08.027
10. Bandinelli S, Ferrucci L, Corsi AM, et al. Anemia Is Associated with Disability and Decreased Physical Performance and Muscle Strength in the Elderly. *J Am Geriatr Soc*. 2004;52(5):719-724. doi:10.1111/j.1532-5415.2004.52208.x
11. Thein M, Ershler WB, Artz AS, et al. Diminished quality of life and physical function in community-dwelling elderly with anemia. *Medicine (Baltimore)*. 2009;88(2):107-114. doi:10.1097/MD.0b013e31819d89d5
12. Conlon NP, Bale EP, Herbison GP, McCarroll M. Postoperative Anemia and Quality of Life After Primary Hip Arthroplasty in Patients Over 65 Years Old. *Anesth Analg*. 2008;106(4):1056-1061. doi:10.1213/ane.0b013e318164f114
13. Lawrence VA, Silverstein JH, Cornell JE, Pederson T, Noveck H, Carson JL. Higher Hb level is associated with better early functional recovery after hip fracture repair. *Transfusion*. 2003;43(12):1717-1722. doi:10.1046/j.0041-1132.2003.00581.x
14. Foss NB, Kristensen MT, Kehlet H. Anaemia impedes functional mobility after hip fracture surgery. *Age Ageing*. 2008;37(2):173-178. doi:10.1093/ageing/afm161
15. Fukushima T, Nakano J, Ishii S, et al. Influence of Hemoglobin Level on Muscle and Physical Functions, Activities of Daily Living, and Quality of Life in Patients With Hematological Malignancies. *Integr Cancer Ther*. 2019;18:1-7. doi:10.1177/1534735419842196
16. Morishita S, Kaida K, Yamauchi S, et al. Relationship between corticosteroid dose and declines in physical function among allogeneic hematopoietic stem cell transplantation patients. *Support Care Cancer*. 2013;21(8):2161-2169. doi:10.1007/s00520-013-1778-7
17. Peuranp € A € A P, Heli S, Ovaara-Peippo €, Fraser I, Paavonen J, Ritva Hurskainen &. Effects of anemia and iron deficiency on quality of life in women with heavy menstrual bleeding. doi:10.1111/aogs.12394
18. Roubinian NH, Murphy EL, Mark DG, et al. Long-Term Outcomes Among Patients Discharged From the Hospital With Moderate Anemia. *Ann Intern Med*. 2019;170(2):81. doi:10.7326/M17-3253
19. Docherty AB, Turgeon AF, Walsh TS. Best practice in critical care: anaemia in acute and critical illness. *Transfus Med*. 2018;28(2):181-189. doi:10.1111/tme.12505
20. Bateman AP, McArdle F, Walsh TS. Time course of anemia during six months follow up following intensive care discharge and factors associated with impaired recovery of erythropoiesis. *Crit Care Med*. 2009;37(6):1906-1912. doi:10.1097/CCM.0b013e3181a000cf
21. St Sauver JL, Grossardt BR, Finney Rutten LJ, et al. Rochester Epidemiology Project Data Exploration Portal. *Prev*

Chronic Dis. 2018;15:E42. doi:10.5888/pcd15.170242
22. Rocca WA, Yawn BP, St. Sauver JL, Grossardt BR, Melton LJ. History of the Rochester Epidemiology Project: Half a Century of Medical Records Linkage in a US Population. *Mayo Clin Proc*. 2012;87(12):1202-1213. doi:10.1016/J.MAYOCP.2012.08.012
23. Needham DM, Sepulveda KA, Dinglas VD, et al. Core outcome measures for clinical research in acute respiratory failure survivors an international modified delphi consensus study. *Am J Respir Crit Care Med*. 2017;196(9):1122-1130. doi:10.1164/rccm.201702-0372OC
24. Wu Y, Spaulding AC, Borkar S, et al. Reducing Blood Loss by Changing to Small Volume Tubes for Laboratory Testing. *Mayo Clin Proc Innov Qual Outcomes*. 2021;5(1):72-83. doi:10.1016/J.MAYOCPIQO.2020.08.007
25. Siegal DM, Belley-Cote E, Arnold DM, et al. Small-Volume Tubes to Reduce Anemia and Transfusion (STRATUS): A Pragmatic Stepped Wedge Cluster Randomized Trial. *Blood*. 2019;134(Supplement_1):3685-3685. doi:10.1182/BLOOD-2019-130257
26. Barreda Garcia J, Xian JZ, Pedroza C, et al. Pediatric size phlebotomy tubes and transfusions in adult critically ill patients: A pilot randomized controlled trial. *Pilot Feasibility Stud*. 2020;6(1):1-9. doi:10.1186/s40814-020-00657-3
27. Corwin HL, Gettinger A, Pearl RG, et al. Efficacy of Recombinant Human Erythropoietin in Critically Ill Patients. *JAMA*. 2002;288(22):2827. doi:10.1001/jama.288.22.2827
28. Corwin HL, Gettinger A, Fabian TC, et al. Efficacy and Safety of Epoetin Alfa in Critically Ill Patients. *N Engl J Med*. 2007;357(10):965-976. doi:10.1056/NEJMoa071533
29. Corwin HL, Gettinger A, Rodriguez RM, et al. Efficacy of Recombinant Human Erythropoietin in the Critically Ill Patient. *Crit Care Med*. 1998;26(Supplement):23A. doi:10.1097/00003246-199801001-00007
30. Shah A, Fisher SA, Wong H, et al. Safety and efficacy of iron therapy on reducing red blood cell transfusion requirements and treating anaemia in critically ill adults: A systematic review with meta-analysis and trial sequential analysis. *J Crit Care*. 2019;49:162-171. doi:10.1016/j.jcrc.2018.11.005
31. Lasocki S, Asfar P, Jaber S, et al. Impact of treating iron deficiency, diagnosed according to hepcidin quantification, on outcomes after a prolonged ICU stay compared to standard care: a multicenter, randomized, single-blinded trial. *Crit Care*. 2021;25(1):1-10. doi:10.1186/s13054-020-03430-3
32. The IRONMAN Investigators, Litton E, Baker S, et al. Intravenous iron or placebo for anaemia in intensive care: the IRONMAN multicentre randomized blinded trial: A randomized trial of IV iron in critical illness. *Intensive Care Med*. 2016;42(11):1715-1722. doi:10.1007/s00134-016-4465-6
33. Richards T, Clevenger B, Keidan J, et al. PREVENTT: Preoperative intravenous iron to treat anaemia in major surgery: Study protocol for a randomised controlled trial. *Trials*. 2015;16(1). doi:10.1186/s13063-015-0774-2
34. Chertow GM, Mason PD, Vaage-Nilsen O, Ahlme J. Update on adverse drug events associated with parenteral iron. *Nephrol Dial Transpl*. 2006;21:378-382. doi:10.1093/ndt/gfi253
35. Litton E, Latham P, Inman J, Luo J, Allan P. Safety and efficacy of erythropoiesis-stimulating agents in critically ill patients admitted to the intensive care unit: a systematic review and meta-analysis. *Intensive Care Med*. 2019;45(9):1190-1199. doi:10.1007/s00134-019-05686-y
36. M H, R L, C R, et al. Erythropoietin to treat head and neck cancer patients with anaemia undergoing radiotherapy: randomised, double-blind, placebo-controlled trial. *Lancet (London, England)*. 2003;362(9392):1255-1260. doi:10.1016/S0140-6736(03)14567-9
37. JR W, YC U, JA J, et al. Randomized, double-blind, placebo-controlled trial of erythropoietin in non-small-cell lung cancer with disease-related anemia. *J Clin Oncol*. 2007;25(9):1027-1032. doi:10.1200/JCO.2006.07.1514
38. B L-J, V S, M P, et al. Maintaining normal hemoglobin levels with epoetin alfa in mainly nonanemic patients with metastatic breast cancer receiving first-line chemotherapy: a survival study. *J Clin Oncol*. 2005;23(25):5960-5972. doi:10.1200/JCO.2005.06.150
39. Choi MJ, Yee J. Erythropoiesis-Stimulating Agents and Cancer: Myth or Truth. Published online 2019. doi:10.1053/j.ackd.2019.04.001
40. J B, K B, R C, et al. Management of Cancer-Associated Anemia With Erythropoiesis-Stimulating Agents: ASCO/ASH Clinical Practice Guideline Update. *J Clin Oncol*. 2019;37(15):1336-1351. doi:10.1200/JCO.18.02142
41. Warner MA, Shore-Lesserson L, Shander A, Patel SY, Perelman SI, Guinn NR. Perioperative Anemia. *Anesth Analg*.

Published online March 2020:1. doi:10.1213/ANE.0000000000004727

42. Litton E, Latham P, Inman J, Luo J, Allan P. Safety and efficacy of erythropoiesis-stimulating agents in critically ill patients admitted to the intensive care unit: a systematic review and meta-analysis. *Intensive Care Med*. 2019;45(9):1190-1199. doi:10.1007/s00134-019-05686-y
43. Lasocki S, Puy H, Mercier G, et al. Impact of iron deficiency diagnosis using hepcidin mass spectrometry dosage methods on hospital stay and costs after a prolonged ICU stay: Study protocol for a multicentre, randomised, single-blinded medico-economic trial. *Anaesth Crit Care Pain Med*. 2017;36(6):391-396. doi:10.1016/j.accpm.2017.04.009
44. Pieracci FM, Barie PS. Iron and the Risk of Infection. *Surg Infect (Larchmt)*. 2005;6(s1):s41-s46. doi:10.1089/sur.2005.6.s1-41
45. Geisser P, Burckhardt S. The pharmacokinetics and pharmacodynamics of iron preparations. *Pharmaceutics*. 2011;3(1):12-33. doi:10.3390/pharmaceutics3010012
46. Warner MA, Shore-Lesserson L, Shander A, Patel SY, Perelman SI, Guinn NR. Perioperative Anemia. *Anesth Analg*. Published online March 2020:1. doi:10.1213/ane.0000000000004727
47. Spahn DR, Schoenrath F, Spahn GH, et al. Effect of ultra-short-term treatment of patients with iron deficiency or anaemia undergoing cardiac surgery: a prospective randomised trial. *Lancet*. 2019;6736(18):1-12. doi:10.1016/S0140-6736(18)32555-8
48. Pieracci FM, Henderson P, Rodney JRM, et al. Randomized, Double-Blind, Placebo-Controlled Trial of Effects of Enteral Iron Supplementation on Anemia and Risk of Infection during Surgical Critical Illness. *Surg Infect (Larchmt)*. 2009;10(1):9-19. doi:10.1089/sur.2008.043
49. Pieracci FM, Stovall RT, Jaouen B, et al. A multicenter, randomized clinical trial of IV iron supplementation for anemia of traumatic critical illness*. *Crit Care Med*. 2014;42(9):2048-2057. doi:10.1097/CCM.0000000000000408
50. Dinglas VD, Faraone LN, Needham DM. Understanding patient-important outcomes after critical illness. *Curr Opin Crit Care*. 2018;24(5):401-409. doi:10.1097/MCC.0000000000000533
51. Roberts RO, Geda YE, Knopman DS, et al. The Mayo Clinic Study of Aging: Design and sampling, participation, baseline measures and sample characteristics. *Neuroepidemiology*. 2008;30(1):58-69. doi:10.1159/000115751

The Practical Anemia Bundle for Sustained Blood Recovery (PABST-BR) in Critical Illness Randomized Clinical Trial

Protocol Number: IRB # 21-006511

Principal Investigator: Matthew A. Warner, MD

Funded by: NHLBI

Initial version: 5 October 2021

Revised: 11 October 2022

Table of Contents

STATEMENT OF COMPLIANCE 1
1 PROTOCOL SUMMARY 1
1.1 Synopsis 1
1.2 Schema 3
1.3 Schedule of Activities (SoA) 4
2 INTRODUCTION 4
2.1 Study Rationale 4
2.2 Background 5
2.3 Risk/Benefit Assessment 7
2.3.1 Known Potential Risks 7
2.3.2 Known Potential Benefits 8
2.3.3 Assessment of Potential Risks and Benefits 8
3 OBJECTIVES AND ENDPOINTS 10
4 STUDY DESIGN 11
4.1 Overall Design 11
4.2 Scientific Rationale for Study Design 13
4.3 Justification for Dose 13
4.4 End of Study Definition 13
5 STUDY POPULATION 14
5.1 Inclusion Criteria 14
5.2 Exclusion Criteria 14
5.3 Lifestyle Considerations 14
5.4 Screen Failures 15
5.5 Strategies for Recruitment and RetentioN 15
6 STUDY INTERVENTION 16
6.1 Study Intervention(s) Administration 16
6.1.1 Study Intervention Description 16
6.2 Preparation/Handling/Storage/Accountability 17
6.2.1 Acquisition and accountability 17
6.2.2 Formulation, Appearance, Packaging, and Labeling 17
6.2.3 Product Storage and Stability 17
6.2.4 Preparation 18
6.3 Measures to Minimize Bias: Randomization and Blinding 18
6.4 Study Intervention Compliance 18
6.5 Concomitant Therapy 18
6.5.1 Rescue Medicine 18
7 STUDY INTERVENTION DISCONTINUATION AND PARTICIPANT DISCONTINUATION/WITHDRAWAL. 18
7.1 Discontinuation of Study Intervention 18
7.2 Participant Discontinuation/Withdrawal from the Study 19

7.3 Lost to Follow-Up 19
8 STUDY ASSESSMENTS AND PROCEDURES 19
8.1 Efficacy Assessments 20
8.2 Safety and Other Assessments 20
8.3 Adverse Events and Serious Adverse Events 20
8.3.1 Definition of Adverse Events (AE) 20
8.3.2 Definition of Serious Adverse Events (SAE) 21
8.3.3 Classification of an Adverse Event 21
8.3.4 Time Period and Frequency for Event Assessment and Follow-Up 22
8.3.5 Adverse Event Reporting 22
8.3.6 Serious Adverse Event Reporting 23
8.3.7 Reporting Events to Participants 23
8.3.8 Events of Special Interest 23
8.3.9 Reporting of Pregnancy 23
8.4 Unanticipated Problems 23
8.4.1 Definition of Unanticipated Problems (UP) 23
8.4.2 Unanticipated Problem Reporting 23
8.4.3 Reporting Unanticipated Problems to Participants 24
9 STATISTICAL CONSIDERATIONS 24
9.1 Statistical Hypotheses 24
9.2 Sample Size Determination 24
9.3 Populations for Analyses 25
9.4 Statistical Analyses 25
9.4.1 General Approach 25
9.4.2 Analysis of the Primary Efficacy Endpoint(s) 25
9.4.3 Analysis of the Secondary Endpoint(s) 26
9.4.4 Safety Analyses 27
9.4.5 Baseline Descriptive Statistics 27
9.4.6 Planned Interim Analyses 27
9.4.7 Sub-Group Analyses 27
9.4.8 Tabulation of Individual participant Data 27
9.4.9 Exploratory Analyses 27
10 SUPPORTING DOCUMENTATION AND OPERATIONAL CONSIDERATIONS 27
10.1 Regulatory, Ethical, and Study Oversight Considerations 27
10.1.1 Informed Consent Process 27
10.1.2 Study Discontinuation and Closure 28
10.1.3 Confidentiality and Privacy 28
10.1.4 Future Use of Stored Specimens and Data 29
10.1.5 Key Roles and Study Governance 29
10.1.6 Safety Oversight 29
10.1.7 Clinical Monitoring 29

10.1.8 Quality Assurance and Quality Control 30
10.1.9 Data Handling and Record Keeping 30
10.1.10 Protocol Deviations 31
10.1.11 Publication and Data Sharing Policy 31
10.1.12 Conflict of Interest Policy 31
10.2 Additional Considerations 31
10.3 Abbreviations 35
10.4 Protocol Amendment History .... **Error! Bookmark not defined.**
11 REFERENCES 37

STATEMENT OF COMPLIANCE

The trial will be carried out in accordance with International Conference on Harmonisation Good Clinical Practice (ICH GCP) and the following:

- United States (US) Code of Federal Regulations (CFR) applicable to clinical studies (45 CFR Part 46, 21 CFR Part 50, 21 CFR Part 56, 21 CFR Part 312, and/or 21 CFR Part 812)

National Institutes of Health (NIH)-funded investigators and clinical trial site staff who are responsible for the conduct, management, or oversight of NIH-funded clinical trials have completed Human Subjects Protection and ICH GCP Training.

The protocol, informed consent form(s), recruitment materials, and all participant materials will be submitted to the Institutional Review Board (IRB) for review and approval. Approval of both the protocol and the consent form must be obtained before any participant is enrolled. Any amendment to the protocol will require review and approval by the IRB before the changes are implemented to the study. In addition, all changes to the consent form will be IRB-approved; a determination will be made regarding whether a new consent needs to be obtained from participants who provided consent, using a previously approved consent form.

1 PROTOCOL SUMMARY

1.1 SYNOPSIS

Title: The Practical Anemia Bundle for SusTained Blood Recovery (PABST-BR) in Critical Illness Randomized Clinical Trial

Study Description: The goal of this investigation is to test a multi-faceted anemia prevention and targeted treatment bundle (optimized phlebotomy, decision support, targeted pharmacologic treatment) to attenuate anemia development and promote hemoglobin and functional recovery in adults with critical illness. We hypothesize that the bundle will improve hemoglobin recovery and functional outcomes through 3 months post-hospitalization.

Objectives: Primary Objective: To assess the efficacy of the intervention on mean difference in hemoglobin concentrations at 1 month after hospitalization.
Secondary Objectives:
To assess the impact of the intervention on:

- hemoglobin concentrations through 3-months post hospitalization
- functional outcomes (Core Outcome Measurement Set [COMS], PROMIS-FATIGUE) at 1 and 3 months post-hospitalization
- RBC transfusions through 3 months post-hospitalization
- hospital readmissions through 12 months post-hospitalization
- mortality through 12 months post-hospitalization

Endpoints: Primary Endpoint: Hemoglobin concentrations (mean difference in hemoglobin concentrations at 1-month post-hospitalization)
Secondary Endpoints:

- Hemoglobin concentrations (through 3 months post-hospitalization)
- Functional outcomes (EQ-5D, PROMIS-FATIGUE, 6-minute walk distance, daily step counts and energy expenditure, activities of daily living survey, MOCA-BLIND, HADS, IES-R) at 1 and 3-months postop-hospitalization
- RBC transfusions through 3-months post-hospitalization

	• Readmissions in first 12-months post-hospitalization<br>• All-cause mortality through 12 months post-hospitalization
**Study Population:**	100 patients, male and females, age $\geq$ 18 years, moderate-to-severe comorbid illness burden, moderate-to-severe acute illness burden, in ICUs at Mayo Clinic Rochester
**Phase:**	2
**Description of Sites/Facilities Enrolling Participants:**	ICUs at Mayo Clinic Rochester, MN; a large tertiary care academic medical center
**Description of Study Intervention:**	Participants will be randomized 1:1 to active intervention vs. standard of care using a stratified permuted block design by anemia type (iron-responsive vs. inflammatory) and ICU admission indication (surgical vs. non-surgical).<br><br>The intervention arm is multi-faceted with 3 primary components:<br>1) **Optimized phlebotomy**, defined by:<br>• minimal volume draws<br>• closed-loop blood sampling<br>• bundling of similarly timed labs, all performed by a dedicated phlebotomy team independent from the treatment team<br><br>2) **Decision support aids**, including:<br>• visual and electronic alerts reminding the care team to minimize non-essential laboratory testing, mitigate patient-specific bleeding risk<br>• daily communication with the ICU care team during clinical rounds to reiterate the purpose of the study<br><br>3) **Pharmacologic anemia treatment** (given immediately following randomization) targeted to 2 broad groups:<br>1) Anemia responsive to iron supplementation (i.e. 1000 mg IV low molecular weight iron dextran), and<br>2) Anemia of inflammation requiring erythropoietic stimulation (i.e. 40,000 units of subcutaneous erythropoietin +/- iron supplementation as needed to augment iron stores prior to EPO dosing)
**Study Duration:**	2 years
**Participant Duration:**	3 months post-hospitalization, EMR review by study staff at 12 months post-hospitalization

1.2 SCHEMA

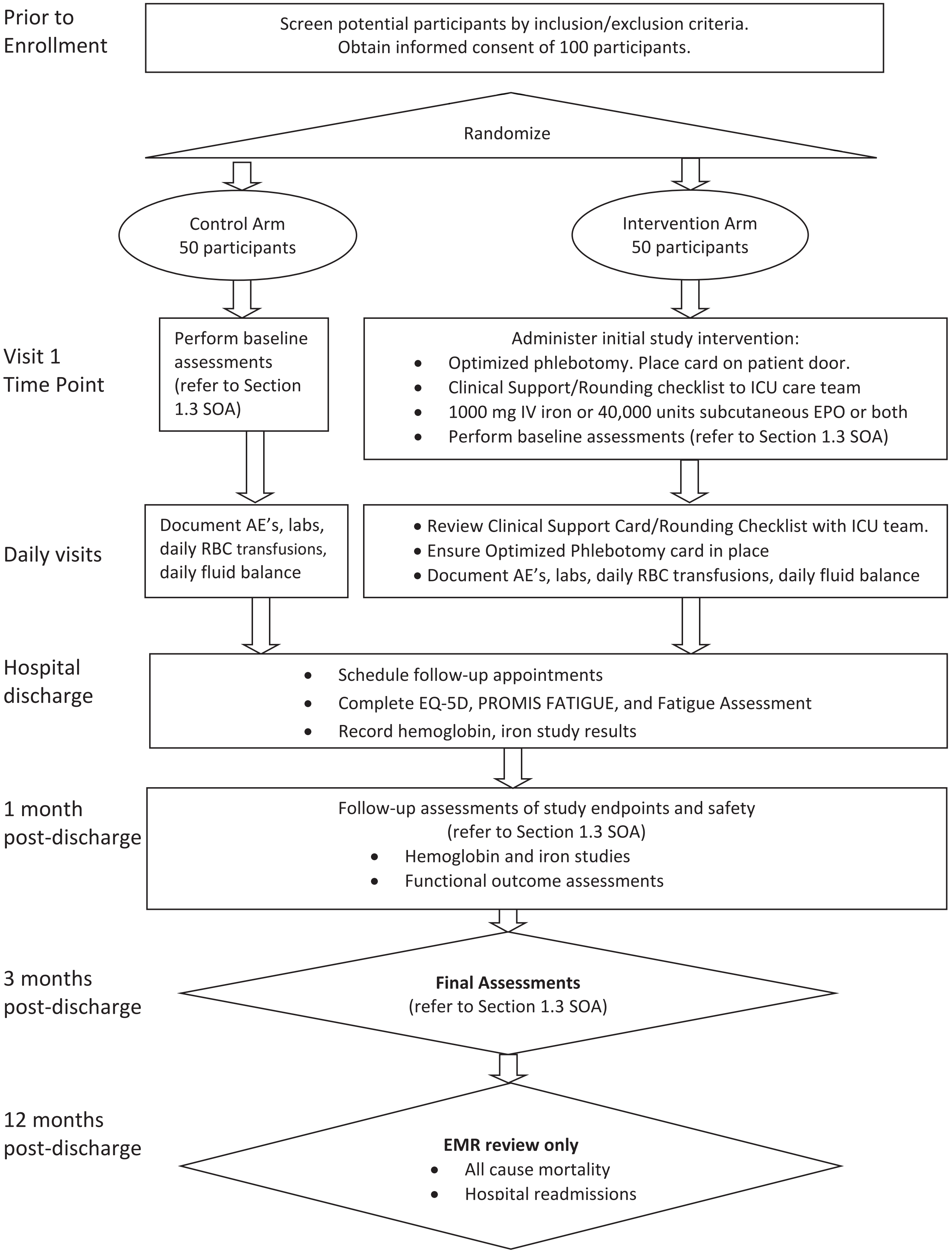
Prior to Enrollment
Screen potential participants by inclusion/exclusion criteria.
Obtain informed consent of 100 participants.
Randomize
Control Arm
50 participants
Intervention Arm
50 participants
Visit 1 Time Point
Perform baseline assessments (refer to Section 1.3 SOA)
Administer initial study intervention:
• Optimized phlebotomy. Place card on patient door.
• Clinical Support/Rounding checklist to ICU care team
• 1000 mg IV iron or 40,000 units subcutaneous EPO or both
• Perform baseline assessments (refer to Section 1.3 SOA)
Daily visits
Document AE's, labs, daily RBC transfusions, daily fluid balance
• Review Clinical Support Card/Rounding Checklist with ICU team.
• Ensure Optimized Phlebotomy card in place
• Document AE's, labs, daily RBC transfusions, daily fluid balance
Hospital discharge
• Schedule follow-up appointments
• Complete EQ-5D, PROMIS FATIGUE, and Fatigue Assessment
• Record hemoglobin, iron study results
1 month post-discharge
Follow-up assessments of study endpoints and safety
(refer to Section 1.3 SOA)
• Hemoglobin and iron studies
• Functional outcome assessments
3 months post-discharge
Final Assessments
(refer to Section 1.3 SOA)
12 months post-discharge
EMR review only
• All cause mortality
• Hospital readmissions

1.3 SCHEDULE OF ACTIVITIES (SOA)

Procedures	Enrollment/ Baseline Study day 1	Daily visits throughout hospitalization	Hospital Discharge	1-month post-hospitalization +/- 7 days	3-months post-hospitalization +/- 14 days	12-months post-hospitalization EMR review
Informed consent	X					
Demographics	X					
Medical history	X					
Randomization	X					
Administer study intervention	X					
Vital signs- (BP, HR, RR, O2 sat)	X					
Height	X					
Weight	X		X			
Pregnancy test if needed	X					
Anemia labs[a]	X	X	X	X	X	
Platelet count[d]	X		X			
White blood cell count[d]	X		X			
Creatinine[d]	X		X			
Functional outcome assessment [b]	X		X	X	X	
Con medication review [c]	X	X				
Fluid balance	X		X			
RBC transfusions documented	X	X	X	X	X	
Daily phlebotomies for labs (excluding RMGs)		X	X			
Fatigue assessment			X	X	X	
AE review and evaluation	X	X	X	X	X	
Research Samples[e]	X			X	X	
Hospital readmissions documentation				X	X	X
All cause mortality status						X
Complete Case Report Forms	X	X	X	X	X	X

a. **Hemoglobin, ferritin, and transferrin saturation will be obtained for all patients at enrollment, hospital discharge, 1-month, and 3-months post-hospitalization.** Other anemia labs to be reported at these and other timepoints include, if available Hemoglobin, MCV, RDW, ferritin, iron, transferrin saturation, reticulocyte hemoglobin, absolute reticulocyte count; record all values available recognizing that not all non-hemoglobin values will be available at each interval.
b. Functional outcomes according to the Core Outcome Measurement Set will be obtained in the following fashion: **Enrollment** – ADL survey (by patient or proxy); at **Hospital discharge** – EQ-5D, PROMIS-FATIGUE; **at 1-month and 3-months** – EQ-5D, PROMIS-FATIGUE, 6MWD, ADL Survey, MoCA-BLIND, HADS, IES-R, activity monitoring (optional study component).
c. Concomitant meds review to include y/n for antiplatelet agents (i.e. aspirin, clopidogrel), anticoagulants (i.e. heparin, warfarin, direct oral anticoagulants, low molecular weight heparins), iron, and erythropoiesis stimulating agent use (i.e. darbepoetin, EPO). Daily visits only need to report if they received iron or EPO/darbepoetin.
d. Platelets, WBC and Creatinine will be collected if done as standard of care. Baseline results used should be from within 24 hrs prior to enrollment. Hospital discharge results used should be the closest to discharge up to 72 hours prior.
e. Research samples (10 ml phlebotomy) will be obtained at enrollment, 1-month, and 3-months and stored for potential biomarker assessment (i.e. hepcidin, IL-6, C-reactive protein) for future mechanistic studies on anemia development and recovery in critical illness survivors.

2 INTRODUCTION

2.1 STUDY RATIONALE

Anemia is common in the critically ill and is associated with poor patient outcomes both during and after hospitalization. Recent data suggests that anemia may also represent a potentially modifiable risk factor for impaired post-hospitalization physical function. The goal of this investigation is to test a multi-faceted anemia prevention and targeted treatment bundle (optimized phlebotomy practice, decision support, targeted pharmacologic anemia treatment) to attenuate anemia development and promote hemoglobin and functional recovery in the setting of critical illness. Specifically, we aim to assess the impact of the intervention on hemoglobin concentrations and functional outcomes (i.e. physical, cognitive, mental health) through 3-months after hospitalization.

2.2 BACKGROUND

Anemia is remarkably common in the critically ill.[1–3] Over the last 20 years, clinicians have become more tolerant of anemia during hospitalization and critical illness, a phenomenon driven in large part by the landmark Transfusion Requirements in Critical Care (TRICC) trial,[4] which revealed similar 30-day mortality with restrictive versus liberal red blood cell (RBC) transfusion strategies. However, we now realize that survival of critical illness does not guarantee the quality of the life preserved. Up to 50% of survivors have substantial functional deficits in one or multiple domains related to physical function, cognition, mental health, and quality of life.[5] This is increasingly relevant given the growing number of survivors of COVID-19-related critical illness.[6] It is therefore paramount that we identify modifiable risk factors for impaired functional recovery in ICU survivors, of which anemia may be one potential target.[7] Importantly, anemia has consistently been linked with impaired functional outcomes in non-critically ill populations, including the elderly,[8–11] post-surgical patients,[12–14] and those with hematologic disease[15,16] and menstrual bleeding.[17] However, our knowledge of post-hospitalization recovery from anemia in survivors of critical illness and its relationship with patient outcomes is limited, despite increasing anemia prevalence at the time of hospital discharge.[3,18,19]

Regarding post-hospitalization hemoglobin recovery, a prospective investigation of 19 critically ill patients with anemia discovered that approximately 50% remained anemic 6-months later.[20] In our data from 6460 survivors of critical illness enrolled in large population-based health study,[21,22] 80% of subjects were discharged from the hospital with anemia.[3] At 12 months post-hospitalization, only half of those alive with available hemoglobin assessments had recovered to non-anemic status, with recovery varying in accordance with anemia severity at hospital discharge. These data confirm that for many patient's anemia persists long after the resolution of critical illness. Additionally, higher hemoglobin concentrations at hospital discharge were associated with reduced post-hospitalization mortality in adjusted analyses (HR 0.95 [95% CI 0.90-0.99], per 1 g/dL increase; p=0.020), suggesting that anemia may have important implications for downstream clinical outcomes. Preliminary data from this cohort also shows that hemoglobin concentrations are strongly associated with unplanned hospital readmissions in the first 30-days after hospital discharge, with each 1 g/dL increase in hemoglobin associated with a 15% reduction in the instantaneous hazard for readmission after multivariable adjustment (HR 0.85, 95% CI 0.78, 0.93; p< 0.001; unpublished). Patient readmission status over time by the severity of anemia at hospital discharge for critical illness survivors is shown in Figure 1.

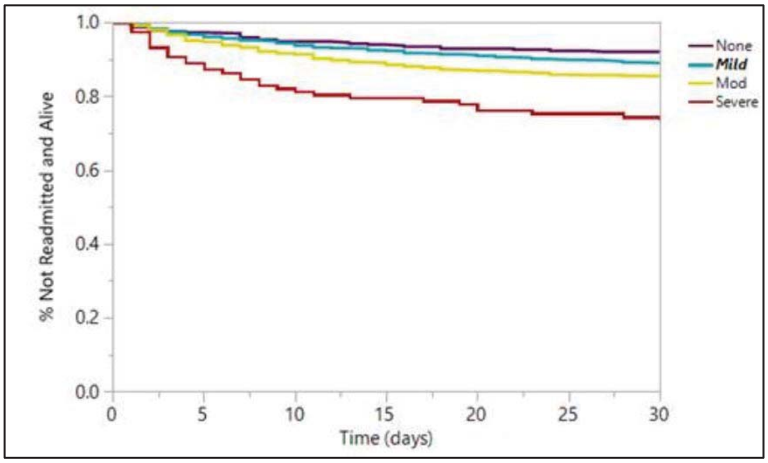

Figure 1. The percentage of patients still alive and not readmitted to the hospital (y-axis) over time (x-axis) is shown for critical illness survivors without anemia (hemoglobin >12 g/dL females, >13 g/dL males) and with mild (hemoglobin 10-12 g/dL female, 10-13 g/dL male), moderate (hemoglobin 8-10 g/dL), and severe anemia (hemoglobin < 8 g/dl) at hospital discharge.

While the relationships between anemia and functional recovery after critical illness remain incompletely defined, our recent data from a multi-center prospective cohort of 195 survivors of acute respiratory distress syndrome discovered that anemia after critical illness was associated with impaired physical function 3-months later,[7] including reductions in ambulatory capacity (i.e. 6-minute walk distance [6MWD]) and activities of daily living (ADLs). This suggests that anemia may represent a modifiable risk factor for improved physical outcomes after critical illness. However, relationships with other functional outcomes (cognition, mental health, quality of life) remain unknown, and it is unclear what effect the extent and timing of recovery from anemia after critical illness may have on outcomes. These multidimensional functional outcomes have been deemed of the highest importance for contemporary critical illness research.[23]

Anemia management strategies include prevention, attenuation, and treatment. Prevention and attenuation strategies are largely related to minimizing iatrogenic anemia development or progression secondary to phlebotomy and hemodilution. Most prominently, this includes the use of low-volume blood sampling strategies. Observational suggests that these low-volume blood draw strategies may decrease iatrogenic blood loss and transfusions,[24] though clinical trial data is limited.[25,26] In addition to low-volume phlebotomy, the use of clinical decision support is another method to promote appropriate lab utilization and to minimize excessive blood draws, though this has not been formally studied in critically ill patients. Regarding non-transfusion-based anemia treatment options, iron and erythropoietin (EPO) have been used in numerous clinical trials in the setting of critical illness.[27–32] While these therapies have consistently augmented hemoglobin recovery during hospitalization, they have had inconsistent results on RBC reductions and mortality, for which reason they have not been widely adopted into clinical practice. However, we now recognize that survival of hospitalization is not the final hurdle for critical illness survivors, and there is increasing recognition that anemia may contribute to persistent post-hospitalization impairments in daily functioning.[7] As such, strategies to augment hemoglobin recovery during critical illness may favorably influence post-hospitalization outcomes, though this remains incompletely studied. In one recent randomized clinical trial of intravenous iron with or without EPO therapy administered at the time of ICU discharge versus standard care, there was no difference between groups in the primary outcome of post-hospitalization length of stay. However, patients receiving the treatment had a significant reduction in 90-day mortality (17% vs. 8%). Additionally, there is growing evidence in surgical patients that anemia treatment can have positive consequences for patients that extend beyond hospitalization.[33] Hence, future research is clearly warranted.

Briefly, this is a randomized clinical trial of a multifaceted anemia prevention and treatment bundle versus standard of care to assess the impact of the intervention on hemoglobin recovery and post-hospitalization functional outcomes. Each patient randomized to the intervention will receive 3 treatment components: 1) optimized phlebotomy practice; 2) clinical decision support; and 3) pharmacologic anemia treatment. With regards to pharmacologic anemia treatment, patients may receive either a single dose of intravenous (IV) iron therapy in isolation if they have an iron-responsive anemia or a single dose of EPO if they have an anemia of inflammation requiring erythropoietic stimulation. Further, some patients receiving EPO may also receive a single dose of IV iron to replenish iron stores prior to EPO administration

(i.e. if serum ferritin is < 1000 ng/ml at the time of randomization). Patients randomized to the standard of care arm will receive usual ICU cares.

2.3 RISK/BENEFIT ASSESSMENT

2.3.1 KNOWN POTENTIAL RISKS

There is minimal patient risk associated with the employment of 1) optimized phlebotomy practices, or 2) decision-support aids. Changes to phlebotomy practice include: default low-volume blood sampling (e.g. 0.1-2 ml for most lab draws rather than 3-10 ml), which exposes the patient to no direct risk, and utilization of closed-loop blood sampling systems in order to minimize blood draw waste, again with no direct risk to the patient. Low-volume blood sampling is already used clinically in our ICU environments for patients with severe anemia. In rare circumstances (<1%), a low-volume sample may be insufficient for laboratory testing and additional phlebotomy may be required. Closed-loop blood sampling for those with pre-existing arterial or central venous catheters provides a method of returning "waste blood" directly to the patient rather than disposing of this "waste" volume, which typically is 10 ml per sample. It is routinely used throughout our pediatric ICU practices with no harm to patients, including no increased risk of infection. It has not been adopted into adult ICU practice given higher cost. At 1 and 3-month follow-up, patients will again undergo phlebotomy testing. There is a risk of patient discomfort with the additional blood draws at these follow-up assessments, which will be disclosed at the time of enrollment. Patients will retain the right to refuse these blood draws or any other follow-up measures. Additionally, a total of 5cc of blood will be removed for laboratory sampling at each follow-up assessment; this is not expected to have any substantial negative clinical consequence.

The third component of the clinical intervention is the utilization of EPO and/or iron for the treatment of anemia, which does carry tangible patient risks. **Iron** will be administered as a single 1000 mg dose of IV low molecular-weight iron dextran. This is an approved therapy for patients with iron-deficiency anemia that is utilized in current clinical practice for critically ill patients with anemia and contraindications to transfusion therapies (i.e. Jehovah's witness patients) and post-surgical patients after large volume blood loss. The estimated incidence of SAEs with newer IV iron formulations, such as low molecular weight iron dextran, is less than 1 in 250,000 administrations.[34] Nevertheless, immediate risks of IV iron are real and include allergic reactions (<1%) and non-allergic infusions reactions (e.g. myalgias, arthralgias, dizziness, <1%). Additionally, as a long-term risk, repeated doses of iron administration, particularly to patients with iron storage disorders (i.e. hemochromatosis) or those requiring frequent and recurrent RBC transfusions, can culminate in iron overload. Patients with hemochromatosis or elevated iron stores (i.e. ferritin > 1000 ng/ml) will not be eligible to receive iron therapies in this study.

EPO (i.e. Epoetin alpha), the erythropoiesis stimulating agent utilized in this trial, will be administered as a single 40,000 unit subcutaneous injection after iron supplementation, though iron supplementation will be withheld for those with ferritin > 1000 ng/ml. It has been used in previous clinical trials of anemia management in critical illness, and it is utilized in our current clinical practice for critically ill anemic and post-surgical patients with contraindications to transfusion therapies (i.e. Jehovah's witness patients). There are short-term risks associated with this therapy, including minor non-allergic adverse reactions (nausea, dizziness, high blood pressure, pruritis; estimated <10%) and major rare adverse reactions (e.g. deep venous thrombosis, uncontrolled hypertension, myocardial infarction; estimated <1%). There is a black box warning for myocardial infarction, stroke, venous thromboembolism, vascular access thrombosis, and mortality when targeting hemoglobin levels >11 g/dL, data which is derived from <u>repeated</u> use of EPO to achieve near-normal hemoglobin levels for patients with chronic kidney disease. In a recent systematic review and meta-analysis of 21 trials in critical illness, the relative risk (RR) for mortality with EPO was 0.82 (95% CI 0.71-0.94).[35] There were no significant differences in serious adverse events (RR 1.11, 95% CI 0.94-1.31) or VTE (RR 1.17, 95% CI 0.87-1.58), though these data do not exclude the potential for a clinically significant increase in the risk for adverse events with EPO therapy. As a longer-term risk, EPO may theoretically increase the risk of tumor progression or recurrence in patients with cancer. However, these concerns are derived from trials in non-surgical oncologic patients utilizing large doses of ESAs for extended periods of time, particularly when targeting normal hemoglobin levels.[36–38] However, the

overwhelming evidence suggests that EPO use has no significant impact on cancer progression or other adverse oncologic outcomes, particularly when used in low-doses and when pre-treatment hemoglobin concentrations do not exceed 12 g/dL.[39,40] To this end, EPO is used extensively for preoperative anemia management in patients undergoing oncologic surgery.[41] Additionally, EPO is now recommended for the treatment of anemia in critical ill adults by the French Society of Anesthesia & Intensive Care Medicine (SFAR).[42]

2.3.2 KNOWN POTENTIAL BENEFITS

Immediate potential benefits of the intervention include reduced development of iatrogenic anemia (e.g. less blood taken for phlebotomy) and higher hemoglobin concentrations. Additionally, reduced volume phlebotomy techniques have shown transfusion reductions.[24] Further, studies consistently link treatment with iron and/or EPO to higher hemoglobin recovery during critical illness. While the impact of iron on RBC transfusions is equivocal in critical illness, EPO reduces RBC transfusion requirements and may reduce mortality.[42]

Regarding intermediate and long-range potential benefits, observational data suggests that patients with higher hemoglobin concentrations may have improved hospital and post-hospitalization outcomes.[7] Recent clinical trial data also suggests that anemia treatment with iron +/- EPO may reduce mortality through 1-year after hospitalization.[43] Further, trial data in surgical patients suggests that anemia treatment with IV iron leads to fewer hospital readmissions.[33] Hence, patients in the intervention arm may potentially benefit by 1) achieving higher hemoglobin levels, 2) experiencing improvement in functional outcomes after hospitalization (physical, cognitive, mental health, quality of life), 3) experiencing fewer hospital readmissions, and 4) experiencing greater long-term survival. All patients will receive the benefit of being formally evaluated by trained study personnel after critical illness. There are no other direct benefits to participation.

2.3.3 ASSESSMENT OF POTENTIAL RISKS AND BENEFITS

There is negligible patient risk associated with optimized phlebotomy and clinical decision support. Despite being used safely in multiple clinical trials in critical illness, there are tangible risks of IV iron and EPO therapies. Risks for pharmacologic anemia treatments (IV iron, EPO) will be minimized in multiple ways. First, we will exclude patients at highest risk for potential harm from these therapies, as outlined in the table below:

Exclusion Criteria	Rationale
Iron (IV) or Erythropoiesis-Stimulating Agents (ESA) use in 30 days prior to admission	Already receiving anemia treatment
Pregnancy or breastfeeding	Unclear safety of EPO
Weight <40 kg	Unable to standardize EPO dosing
Uncontrolled sepsis*	Potential infectious risk with IV iron
Allergy to IV iron or erythropoietin	Risk for allergic reaction
Inability to receive VTE chemoprophylaxis, apart from those with recent bleeding or surgery	Increased thrombosis risk with EPO
Suspected or active thrombosis or myocardial ischemia	Incremental risk with EPO
Uncontrolled hypertension (SBP ≥190 or DBP ≥110)	Risk for worsening with EPO
Stroke within 3 months prior	Risk for cerebral ischemia with EPO
Mechanical circulatory support devices (e.g. ECMO, ventricular assist device, Impella)	Risk for device thrombosis with EPO

Hb-hemoglobin. VTE-venous thromboembolism; SBP- systolic blood pressure; DBP-diastolic blood pressure.
*Uncontrolled sepsis defined by <48 hours of appropriate antimicrobial therapy and/or lack of definitive source control, given theoretical risk for worsening infection with iron supplementation.[44]

Additionally, pharmacological treatments will be targeted to the underlying etiology of anemia to enhance safety. For example, anemia in acute blood loss and iron deficiency is iron-restricted and likely to respond to iron supplementation alone; hence, the potential benefits of IV iron in this group are high while the potential harms of EPO administration (e.g. thrombosis) likely outweigh any incremental benefit on hemoglobin recovery. Further, IV iron will be given as a single total dose infusion, which provides complete restoration of usable iron for >4 weeks.[45,46] For anemias requiring erythropoietic stimulation, EPO will be given as a single 40,000 unit dose after ensuring restoration of iron stores, a strategy that has resulted in sustained hemoglobin improvement and is widely used in anemia clinics.[46,47] Patients with laboratory evidence (i.e. ferritin > 1000 ng/mL) or clinical conditions (i.e. hemochromatosis) indicating states of iron overload will not receive iron supplementation prior to EPO therapy. A single EPO dose carries a lower risk for thrombotic complications than repeat dosing, which is particularly relevant in the critically ill.

Regarding the safety of IV iron (i.e. low molecular weight iron dextran), this medication is used extensively in modern medical practice. The estimated incidence of serious adverse events (SAEs) is less than 1 in 250,000 administrations.[34] The medication will be administered by critical care nurses who have received training in iron administration in accordance with institutional medication administration protocols. A small test dose of 25 mg will be given over 5 minutes to ensure no symptoms of adverse reaction with continuous assessment by the bedside nurse, including continuous monitoring of pulse oximetry, heart rate, and blood pressure (continuously for those with an invasive arterial catheter, and every 5 minutes for those without) and visual inspection of the patient for rashes or signs of physical and/or respiratory distress. At the discretion of the clinical team, patients deemed to be at high-risk for infusion reactions (e.g. inflammatory arthritis) may be given steroid premedication (e.g. 125 mg methylprednisolone). If a patient develops or is suspected of developing an infusion reaction, therapy will be immediately halted. All infusion reactions will be immediately reported to study personnel for accurate characterization and reporting. Those deemed to have a minor infusion reaction (i.e. rash in absence of hemodynamic or respiratory compromise) will be observed for 15 minutes for signs of clinical progression. If symptoms abate within 15 minutes, the infusion will be restarted at a lower rate. If the patient has persistent mild symptoms, recurrent symptoms, and/or urticaria, they will be treated with an H2-blocker antihistamine (e.g. ranitidine 50 mg), in accordance with institutional policy, prior to restarting the infusion at a lower rate. If there is concern for a moderate infusion reaction or further symptom progression (e.g. hypotension, worsening rash), patients may also receive IV steroids (e.g. methylprednisolone 1-2 mg/kg) +/- a 1L bolus of intravenous isotonic crystalloid (at discretion of ICU team) in addition to H2 blockers. Symptoms should abate completely prior to re-challenging with IV iron, and an alternative iron formulation with comparable dosing should be considered (e.g. iron sucrose). Should a severe reaction be observed (i.e. respiratory distress, anaphylaxis), the patient will receive immediate treatment with IV epinephrine (e.g. 0.1 mg) and additional cardiopulmonary support as dictated by the ICU treatment team. Patients without any apparent reaction to IV iron will be observed clinically for 1 hour post-infusion for the development of delayed reactions with blood pressure measurements at least every 15 minutes, continuous pulse oximetry, and telemetry. Those experiencing infusion reactions will be observed for longer times as dictated by the severity of the reaction. It is commonly thought that iron may predispose patients to bacterial infections;[44] however, this has not been shown in multiple clinical trials in the critically ill.[32,48,49] Out of an abundance of caution, we will exclude patients with uncontrolled sepsis, defined as <48 hours of appropriate antimicrobial therapy and/or lack of definitive source control.

Regarding EPO, several actions are being taken to mitigate risk: 1) excluding patients with relative contraindications to EPO therapy (e.g. pregnancy, active thrombosis, an inability to receive VTE pharmacoprophylaxis, active myocardial ischemia, poorly controlled hypertension, recent stroke); 2) tailoring pharmacotherapy to anemia etiology, such that EPO is not administered to patients that are likely to mount an appropriate erythropoietic response with iron therapy alone; and 3) providing only a single dose of EPO therapy for those receiving this therapy. Of note, the optimal dosing of EPO is unknown in the critically ill, though clinical trials in surgical patients have shown that a single dose of EPO results in sustained hemoglobin improvement.[47] All adverse events will be closely evaluated by study personnel as outlined in the data safety monitoring plan. Similar to iron, EPO will be administered by critical care nurses in accordance with institutional medical administration protocols. Iron will be co-administered immediately prior to EPO to ensure adequate iron stores for EPO-induced augmentation of bone marrow erythropoiesis. Patients will be continuously

monitored at the time of administration for immediate adverse effects (e.g. cutaneous reactions, hypertension, dizziness, nausea). Those without immediate reaction will be observed clinically for 1 hour post-infusion for the development of delayed reactions with blood pressure measurements at least every 15 minutes, continuous pulse oximetry, and telemetry. Those experiencing infusion reactions will be observed for longer times as dictated by the severity of the reaction.

Confidentiality of all participants in the proposed research will be fully protected. Participant privacy and confidentiality will be maintained by conducting the proposed activities in accordance with strict institutional guidelines, which require that formal approval be obtained from all appropriate committees before medical records are reviewed or patient contact is initiated. All study records will be kept in a password-protected study folder and/or locked file cabinets. Individual participants are identified in all computer files and analyses only by a unique study number, which bears no relationship to personal identifiers including name, initials, address, telephone number, social security number, or patient number. All study staff will be trained in HIPAA requirements. Moreover, there is intensive orientation on the confidentiality of medical records and protected health information. Data sharing policies include the requirement that all “identifiers” be removed. All data are tracked in databases by anonymous but linkable study numbers. No identifiable information is explicitly released. During hospitalization, patients will continue to receive care as directed by the primary clinical team. Any adverse reactions to iron and/or EPO will be managed by the primary clinical team per unit-specific protocol. Dosing regiments for these medications have been tailored to minimize any potential risks. Additionally, patients at greatest potential for risk will be excluded. There is minimal risk to patients at follow-up assessments; however, if a patient needs medical evaluation, there is a medical emergency response team immediately available (within 5 minutes) to direct cares, which may include emergency department transfer and/or hospital admission.

3 OBJECTIVES AND ENDPOINTS

OBJECTIVES	ENDPOINTS	JUSTIFICATION FOR ENDPOINTS
Primary		
To assess the efficacy of the intervention on mean difference in hemoglobin concentrations at 1 month post-hospitalization	Hemoglobin concentrations [Time frame: 1 month post-hospitalization]	Hemoglobin concentrations are widely available to infer changes in RBC mass from anemia management interventions
Secondary		
To assess the impact of the intervention on hemoglobin concentrations through 3-months post hospitalization	Hemoglobin concentrations [Time frame: through 3 months post-hospitalization]	Hemoglobin concentrations are widely available to infer changes in RBC mass from anemia management interventions
To assess the impact of the intervention on phlebotomy draws and volumes throughout hospitalization	Phlebotomy draws and volumes [Time frame: through hospitalization]	Phlebotomy draws and volumes are utilized to assess the efficacy of optimized phlebotomy and clinical decision support
To assess the impact of the intervention on quality of life after critical illness	EQ-5D [Time frame: hospital discharge, 1 month, and 3 months post-hospitalization]	Quality of life patient-reported outcome recommended as part of Core Outcome Measurement Set (COMS) for ICU survivors
To assess the impact of the intervention on fatigue after critical illness	PROMIS-Fatigue [Time frame: hospital discharge, 1 month, and 3 months post-hospitalization]	Anemia-related fatigue patient-reported outcome

OBJECTIVES	ENDPOINTS	JUSTIFICATION FOR ENDPOINTS
To assess the impact of the intervention on physical function after critical illness	6-minute walk distance, activity-monitoring daily step count and energy expenditure (optional), activities of daily living survey [Time frame: 1 and 3-months post-hospitalization]	Recommended as part of Core Outcome Measurement Set (COMS) for ICU survivors
To assess the impact of the intervention on cognitive function after critical illness	MoCA-BLIND [Time frame: 1 and 3-months post-hospitalization]	Recommended as part of Core Outcome Measurement Set (COMS) for ICU survivors
To assess the impact of the intervention on mental health after critical illness	HADS, IES-R [Time frame: 1 and 3-months post-hospitalization]	Recommended as part of Core Outcome Measurement Set (COMS) for ICU survivors
To assess the impact of the intervention on RBC transfusions	Allogeneic RBC transfusions (units) [Time frame: hospitalization, through 3-months post-hospitalization]	Anemia management may directly result in reductions in RBC transfusions
To assess the impact of the intervention on unplanned hospital readmissions through 12-months post-hospitalization.	Unplanned hospital readmissions [Time frame: 12 months]	Previous data suggests that anemia treatment with iron therapies may reduce hospital readmissions
To assess the impact of the intervention on mortality through 12-months post-hospitalization	All-cause mortality [Time frame: 12 months]	Previous data suggests that IRON and EPO may reduce mortality during and after critical illness

4 STUDY DESIGN

4.1 OVERALL DESIGN

This is a pragmatic, open-label, single-center, randomized phase 2 pilot clinical trial for superiority of a multi-faceted anemia prevention and treatment strategy assessing the impact of the intervention on hemoglobin concentrations and functional outcomes after hospitalization. We hypothesize that the multifaceted anemia intervention will increase hemoglobin concentrations and will improve multi-faceted functional recovery after hospitalization when compared to standard care.

Patients satisfying inclusion/exclusion criteria, or their legal proxies, will be approached by trained study coordinators for written informed consent. Participants will be randomized 1:1 to active intervention vs. standard care using a stratified permuted block design by anemia etiology (iron-responsive or not) and ICU admission indication (surgical vs. non-surgical). The intervention arm is multi-faceted with 3 primary components: 1) **Optimized phlebotomy**, defined by minimal volume draws (i.e. 0.1-2 ml vs. standard 3-10 ml per laboratory order) and closed-loop blood sampling (eliminates 10 ml waste volume per draw), all performed by a dedicated phlebotomy team independent from the treatment team; 2) **Decision support aids**, including visual and electronic alerts reminding the care team to minimize non-essential laboratory testing and mitigate patient-specific bleeding risk (e.g. stress ulcer prophylaxis in high-risk patients); and 3) **Pharmacologic anemia treatment** (given immediately following enrollment when hemoglobin first observed <10 g/dL) targeted to 2 broad groups: 1) anemias responsive to iron supplementation alone (i.e. acute blood loss, true iron deficiency [i.e. ferritin <100 ng/ml, transferrin saturation < 20%]), and 2) anemias requiring erythropoietic stimulation (e.g. anemia of inflammation, anemia of renal disease). Etiology of anemia will be determined immediately prior to randomization upon review of laboratory values (i.e. iron studies) and clinical history (i.e. admission diagnoses, surgery/acute blood loss) with treatment decisions adjudicated pre-randomization by the PI or Co-I's. Subjects then randomized to the intervention arm will receive pharmacologic anemia treatment in accordance with their anemia

etiology. Patients with anemias responsive to iron supplementation will receive a single total dose infusion (1000 mg) of intravenous (IV) iron dextran. Patients with anemias requiring erythropoietic stimulation will receive a single dose of 40,000 units of subcutaneous EPO, which will be immediately preceded by 1000 mg of IV iron dextran to replenish usable iron stores if the ferritin level is < 1000 ng/ml. All patients randomized to the intervention group will receive either IV iron, EPO, or both. Targeted pharmacologic anemia therapies are being employed given that anemia in acute blood loss/post-surgery and true iron deficiency is iron-restricted and responds to iron supplementation alone; hence, the potential harms of EPO (e.g. thrombosis) likely outweigh benefits.

Outcome assessment:

In-person outcome assessments will occur at 1 month and 3 months post-hospitalization, with investigators and outcome assessors blinded to treatment allocation. The **primary outcome** will be the mean difference in hemoglobin between groups at 1 month. Differences in hemoglobin concentrations will also be assessed at ICU discharge, hospital discharge, and 3 month follow-up. **Secondary outcomes** will include changes in phlebotomy practice (i.e. number of draws, total volume of draws) during hospitalization and functional outcomes at 1 and 3 months, employing the NHLBI-funded Core Outcome Measurement Set (COMS) for survivors of critical illness (aim 2b).[23,50] This includes validated measures of physical function (6MWD, ADL survey), cognition (Montreal Cognitive Assessment Blind), mental health (Hospital Anxiety and Depression Scale, Impact of Events Scale-Revised), and quality of life (EQ-5D). Additionally, as an optional study component, we will solicit patients to wear an activity monitor (Actigraph) on their wrist for 4 consecutive days at 1 and 3-months, from which we will capture daily step counts and energy expenditure measurements. We will also assess several Epic-embedded patient-reported outcomes (i.e. PROMIS) with multidimensional computerized adaptive testing (i.e. PROMIS CAT), laying the groundwork for validation in critical illness research. Differences in RBC transfusion rates will be assessed through 3 month follow-up. We will also assess hospital readmissions and mortality through 12-months post-hospitalization. Research samples obtained will be stored for potential biomarker assessment (i.e. hepcidin, IL-6, C-reactive protein) for future mechanistic studies on anemia development and recovery in critical illness survivors.

Sample size estimation:

Power/sample size are calculated to detect a difference in the primary endpoint of hemoglobin at 1 month follow-up. Preliminary data show mean (standard deviation) hemoglobin levels of 10.8 (1.5) g/dL at 1 month among 636 patients with similar inclusion/exclusion criteria receiving standard care. A total sample size of 74 (37 per group) provides 80% power to detect 1.0 g/dL improvement using a two-sample unequal variances t-test with 2-sided alpha of 0.05 to compare 1 month hemoglobin between randomized arms. Actual power is expected to be higher under the analysis approach, adjusting for pre-randomization prognostic variables to reduce residual variation. If adjustment variables account for 25% of the variation in 1 month hemoglobin (R^2 for relationship between pre-randomization adjustment variables and outcome = 0.25), the sample size of 37 per group provides 90% power to detect a 1.0 g/dL improvement using analysis of covariance with 2-sided alpha of 0.05. While the analysis uses a linear mixed-effects model (LMM), power is not appreciably different for the LMM as compared to the unequal variances t-test or ANCOVA described here. Given expected dropout of up to 25% (death, loss to follow-up) resulting in loss of information, 100 subjects will be enrolled.

Statistical considerations:

The primary outcome is hemoglobin measured repeatedly on subjects at ICU discharge, hospital discharge, and 1 month and 3 months. The longitudinal trajectory of hemoglobin will be analyzed with a LMM. The primary parameter of interest is a treatment group by time interaction to estimate the effect of treatment at each follow up time-point. The treatment effect on 1 month hemoglobin is the primary outcome; other time-points will reflect secondary outcomes. As the functional form of hemoglobin over time is unknown, a discrete time representation will be used. The model will

adjust for pre-randomization hemoglobin, age, sex, anemia etiology, and medical vs surgical ICU setting to reduce residual variation and improve precision of the estimated treatment effect.

Dropout or non-response including skipped study visit and death represent two forms of missing data. Dropout or non-response unrelated to death at ICU discharge and hospital discharge is expected to be negligible but could occur with withdrawal of consent. We assume dropout (unrelated to death) while in the ICU is missing completely at random (MCAR); patients withdrawing consent prior to observation of hemoglobin outcome at ICU discharge will be excluded from the analysis. Those dropping out after ICU discharge are assumed missing at random (MAR) with missingness possibly related to adjustment covariates, arm, or prior observed hemoglobin values. Analyses using LMM assume dropout, nonresponse, or skipped visits are MAR.

We anticipate up to 10% ICU mortality (despite exclusion of those not expected to survive hospitalization); such subjects will not have observed hemoglobin outcomes. We also anticipate additional post-ICU discharge mortality. In the primary analysis, we assume missing data due to death is MAR. Those with ICU mortality will have ICU discharge hemoglobin multiply imputed under the MAR assumption. Thereafter, LMMs assume additional missing data at other times are MAR. In secondary approaches to the analysis of hemoglobin, we use a worst-case imputation approach, to impute hemoglobins as the worst possible outcome following death. After imputation, if residuals are not reasonably normally distributed, a generalized linear mixed effects proportional odds model will be used or individual timepoints may be assessed by Wilcoxon rank-sum test without covariate adjustment. A similar approach using proportional odds models or Wilcoxon rank-sum test will apply to functional outcomes which are not expected to satisfy regression assumptions including normality of residuals. Similar approaches assuming MAR and MNAR will be applied to missing functional outcome data.

4.2 SCIENTIFIC RATIONALE FOR STUDY DESIGN

This is a randomized parallel arm clinical trial comparing the multifaceted anemia intervention against control (standard care). The control group will receive standard care without an active placebo for several reasons: 1) the multifaceted nature of the study intervention makes it difficult to design a placebo for each unique intervention element, particularly clinical decision support and optimized phlebotomy); 2) the expenses associated with administering a placebo for pharmacologic agents (i.e. iron, EPO) are considerable and would not be possible through the NIH K-23 funding mechanism; and 3) this is a pilot trial which will inform a larger, multi-center definitive clinical trial in which we would have the resources available for placebo administration of pharmacologic therapies. Superiority will be assessed as the goal is to improve anemia in the critically ill, given that the problem of anemia is extremely prevalent in the ICU, rather than to prove non-inferiority against standard care.

4.3 JUSTIFICATION FOR DOSE

IV iron given as a single total dose infusion of 1000 mg of IV low molecular weight iron dextran, which provides complete restoration of usable iron for >4 weeks and is the most commonly used dose in clinical practice for patients with iron deficiency.[45,46] For anemias requiring erythropoietic stimulation, EPO will be given as a single 40,000 unit dose after ensuring restoration of iron stores, a strategy that has resulted in sustained hemoglobin improvement and is widely used in anemia clinics.[46,47] A single EPO dose carries a lower risk for thrombotic complications than repeat dosing, which is particularly relevant in the critically ill. Defining optimal treatment dosing and duration remains a priority for future research.

4.4 END OF STUDY DEFINITION

A participant is considered to have completed the study if they have completed all phases of the study including the last visit shown in the Schedule of Activities (SoA), Section 1.3. Data on mortality and hospital readmission will be extracted at 12-months post-hospitalization for each patient by review of the electronic medical record.

5 STUDY POPULATION

5.1 INCLUSION CRITERIA

To be eligible to participate in this study, an individual must meet all the following criteria:

1. Provision of signed and dated informed consent form (may be completed by legal proxies for those patients unable to provide consent, i.e. sedation/intubation)
2. Stated willingness to comply with all study procedures and availability for the duration of the study, including follow-up assessments
3. Male or female, age ≥ 18 years
4. Current ICU admission at Mayo Clinic Rochester
5. Current ICU duration ≤ 7 days
6. Patients embedded in the local or regional Mayo Clinic Health System to facilitate post-hospitalization outcome assessment
7. Moderate-to-severe anemia (i.e. hemoglobin concentration < 10 g/dL) at the time of enrollment, with the hemoglobin concentration assessed no more than 24 hours prior to enrollment. If RBC transfusion has been administered between the qualifying hemoglobin assessment and enrollment, a repeat hemoglobin will be required prior to enrollment to ensure that it remains < 10 g/dL.

5.2 EXCLUSION CRITERIA

An individual who meets any of the following criteria will be excluded from participation in this study:

1. IV iron or any ESA use (i.e. darbepoetin, Aranesp, erythropoietin, Epogen, Procrit, Retacrit) within 30 days of enrollment. Oral iron is permitted (e.g. oral iron supplements, multivitamin with iron)
2. Severe anemia prior to hospitalization (i.e. hemoglobin consistently <9 g/dL in the 90 days prior to admission). If hemoglobin is not available or if hemoglobin is ≥9 g/dL at any time within 90 days prior to enrollment, then this criterion is not met, and the patient may be enrolled
3. Known allergic reactions to IV iron or any EPO agent
4. Inability to complete outcome assessments (i.e. not expected to survive hospitalization, unable to make follow-up appointments, non-ambulatory status preceding hospitalization, dementia or other severe cognitive impairment, visual impairment i.e. blind or legally blind, non-English speaking)
5. Pregnancy or breastfeeding at time of enrollment given unclear safety of EPO
6. Inability to receive pharmacologic venous thromboembolic prophylaxis except in patients with acute blood loss anemia (i.e. recent surgery or gastrointestinal bleeding, extracranial bleeding)
7. Active or suspected thrombosis (i.e. DVT, pulmonary embolism, acute arterial thrombus) within 3 months except in patients with acute blood loss anemia (i.e. recent surgery, gastrointestinal bleeding, extracranial bleeding)
8. Uncontrolled sepsis (i.e. lack of definitive source control and/or <48 hours of appropriate antimicrobial therapy)
9. Having received ≥10 units of allogeneic RBCs in the 48 hours before enrollment
10. Acute coronary syndrome (STEMI or NSTEMI) or ischemic stroke within 3 months except in patients with acute blood loss anemia (i.e. recent surgery, gastrointestinal bleeding, extracranial bleeding)
11. Weight less than 40 kg
12. Concerns with study enrollment expressed by the clinical team

5.3 LIFESTYLE CONSIDERATIONS

Not applicable

5.4 SCREEN FAILURES

Screen failures are defined as participants who consent to participate in the clinical trial but are not subsequently randomly assigned to the study intervention or entered in the study. A minimal set of screen failure information is required to ensure transparent reporting of screen failure participants, to meet the Consolidated Standards of Reporting Trials (CONSORT) publishing requirements and to respond to queries from regulatory authorities. Minimal information includes demography, screen failure details, and eligibility criteria.

Individuals who do not meet the criteria for participation in this trial (screen failure) because of subsequent hemoglobin assessments which prove exclusionary (i.e. hemoglobin > 10 g/dL) may be rescreened as long as they remain in the ICU with a duration not to exceed 7 days at the time of enrollment. Rescreened participants should be assigned the same participant number as for the initial screening.

5.5 STRATEGIES FOR RECRUITMENT AND RETENTION

Recruitment: Trained study coordinators from the Anesthesia Clinical Research Unit will receive daily electronic alerts notifying them of Mayo Clinic Rochester ICU patients ≥ 18 years of age with a most recent hemoglobin value <10 g/dL (infrastructure for these near real-time electronic alerts [2-5 minute delay] are already in place through the ICU DataMart). Patients may be present in any adult ICU. These patients will then be screened through Electronic Health Record (EHR) review by study staff to ascertain eligibility in accordance with inclusion/exclusion criteria. Patients meeting inclusion/exclusion criteria will be approached by study coordinators in their patient room, or by telephone if indicated based on current clinical and/or IRB policies in the setting of COVID-19-related clinical practice/research modifications. Study coordinators will meet with patient or their legal proxies in the case of a patient's inability to directly communicate with study personnel (i.e. deep sedation). The study coordinator will review the research study in detail, explaining the purpose of the study including iron and erythropoietin administration, additional necessary phlebotomy and needed follow up post hospital dismissal. It will be made clear that all participants will receive routine clinical care regardless of whether they 1) agree to participate in the trial, or 2) are randomized to the active treatment arm. The informed consent discussion will occur between the patient and a member of the study team at the patient's bedside during ICU admission as soon as the patient meets eligibility criteria (i.e. within 24 hours). The patient/proxy will be allowed time to have all their questions answered questions answered and assure no exclusions to enrollment are identified. Written informed consent will be obtained by study staff with specific training in this procedure. Signed consent forms will be scanned into the patient's HER and a copy will be provided to the study participant. No information will be used from patients that have not given consent to use their medical information. In the event the LAR consents on behalf of the subject, the study coordinator will make sure and visit with the subject once functional status is reestablished to assure the subject wants to continue with participation. By this time, the subject may/may not have already received study drug, so all they would be affirming is their willingness to continue with the follow up visits/labs/questionnaires.

Retention: A high-level of patient follow-up (>75%) is necessary for success, including in-person evaluations for hemoglobin laboratory draws. To achieve this, we have limited the study to residents residing locally (i.e. patients that receive routine medical cares in Mayo Clinic Rochester or the regional Mayo Clinic Health System), a unique population with a high-level of community engagement in clinical research and a high-level of post-hospitalization medical cares obtained primarily at the Mayo Clinic and its affiliated regional sites. Similar studies of Olmsted County residents, including those with laboratory draws, have achieved >90% retention.[51] Additionally, we are partnering with the Mayo Clinic ICU Rehabilitation Program (MCIRP). The MCIRP is a post-ICU clinic staffed by a physician, advanced ICU care nurse practitioner, ICU pharmacist, and occupational therapist, with the recent addition of a nurse coordinator. In the current care model, survivors of critical illness, identified through real-time electronic data "sniffers", and their family members are approached by MCIRP team members for recruitment prior to hospital discharge. Patients are evaluated within 6 months of hospital discharge with a less than 5% no-show rate for enrolled participants and greater than 90% retention for additional visits. Multi-domain functional outcomes (physical, cognitive, mental health, quality of life) are

assessed at each study visit in accordance with the NHLBI-funded improveLTO Core Outcome Measure Set (*http://improvelto.com).* Of note, with COVID-19 precautions, most MCIRP visits have been moved to virtual visitations. We will work with the MCIRP to ensure appropriate follow-up of all enrolled patients. Our goal remains in-person evaluations for all study participants in an outpatient setting. Of note, all core functional outcomes can be assessed over the phone with exception of 6MWD. *Additional* retention in this clinical trial will be facilitated through employment of published cohort retention tools for longitudinal post-hospitalization critical care outcomes research (*http://improvelto.com)*, which have resulted in >90% cohort retention through 12 months.[21] This includes utilization of a defined cohort retention protocol, careful collection of multiple unique sources of patient contact including proxies, frequent patient engagement, reminder notifications (e.g. reminder phone calls starting 2 weeks before each follow-up appointment and subsequently a phone call 1 week and 1 day before the *appointment)*, remuneration (i.e. $25 per follow-up visit – 1 month and 3 months post-hospitalization), and vouchers for parking (4-hour parking passes, given at each follow-up visit). Recognizing that some patients may die or be lost to contact prior to outcome assessment (~25%), we have increased our study sample size from 74 to 100 participants.

6 STUDY INTERVENTION

6.1 STUDY INTERVENTION(S) ADMINISTRATION

6.1.1 STUDY INTERVENTION DESCRIPTION

The intervention arm is multi-faceted with 3 primary components:

1) **Optimized phlebotomy**, defined by minimal volume draws (i.e. 0.1-2 ml vs. standard 3-10 ml per laboratory order) and closed-loop blood sampling, all performed by a dedicated phlebotomy team independent from the treatment team. This intervention simply requires communication with the phlebotomy team rather than any direct change to patient care.
2) **Decision support**. This includes:
    a. A visual clinical support tool (appendix) urging the clinical team to minimize laboratory assessments and optimize patient bleeding risk. This will be available by the clinical workstations for the ICU team and at the patient's bedside.
    b. A daily rounding checklist (appendix) accompanied by brief daily discussion (2-minutes) between trained study coordinators and the ICU care team during clinical rounds to reiterate the purpose of the study. The checklist will be available at the clinical workstations for the ICU team.
    c. Epic direct messages sent twice per day (targeting day and night providers) with the information from the visual support tool to retitrate the purpose of the study.
3) **Pharmacologic anemia** treatment. These therapies will be given immediately following enrollment and targeted to 2 broad groups:
    a. <u>Anemias responsive to iron supplementation</u> alone. This includes patients with anemia secondary to acute blood loss $\geq$ 500 ml and patients with iron deficiency anemia (i.e. ferritin <100 ng/ml or transferrin saturation < 20%).
        - Patients with iron-responsive anemias will receive a single total dose infusion (1000 mg) of intravenous (IV) low molecular weight iron dextran (INFeD)
        - This medication is diluted in 500 ml of 0.9% saline. A 25 mg test dose (12.5) ml is administered over 5 minutes to ensure no immediate hypersensitivity reaction, followed by the completion of the infusion over 1-2 hours (not to exceed 1000 mg/hr). This medication will be given in accordance with Mayo Clinic administration guidelines.
    b. <u>Anemias requiring erythropoietic stimulation</u>. This includes anemia not secondary to acute blood loss or iron deficiency (e.g. anemia of inflammation, anemia of renal disease). Etiology will be determined immediately *prior to randomization* upon review of laboratory values (i.e. iron studies) and clinical

history (i.e. admission diagnoses, surgery/acute blood loss) with treatment decisions adjudicated pre-randomization by the PI and trial Co-I's should the PI be unavailable.

- Patients without iron-responsive anemias will receive a single dose of 40,000 units of subcutaneous EPO (Retacrit). For those with ferritin less than 1000 ng/ml they will also receive 1000 mg IV iron prior to EPO administration to replenish usable iron, given that iron will invariably be mobilized secondary to EPO stimulation. EPO will be given in accordance with Mayo Clinic administration guidelines.

6.1.2 DOSING AND ADMINISTRATION

Iron dextran shall be administered intravenously as a single dose of 1000 mg (25 mg test dose followed by 975 mg completion dose). EPO shall be administered as a single dose of 40,000 units subcutaneously. There is no patient-specific dose selection. Doses do not need to be modified in relation to meals.

6.2 PREPARATION/HANDLING/STORAGE/ACCOUNTABILITY

6.2.1 ACQUISITION AND ACCOUNTABILITY

This is a multifaceted intervention. Optimized phlebotomy will be coordinated by direct communication to the phlebotomy team by trained study personnel. Clinical decision support will be similarly coordinated by study coordinators. If a patient is randomized to the intervention arm, study coordinators will contact the research pharmacy regarding the need for IV iron +/- EPO. This will be distributed form the research pharmacy to the patient's bedside followed by administration by the bedside critical care nurse. Study coordinators will be available to ensure appropriate administration. Any unused and untampered agents will be immediately returned to the pharmacy.

6.2.2 FORMULATION, APPEARANCE, PACKAGING, AND LABELING

Low molecular weight iron dextran (INFeD®, AbbVie, Inc.), details from package insert: INFeD (iron dextran injection USP) is a dark brown, slightly viscous sterile liquid complex of ferric hydroxide and dextran for intravenous or intramuscular use. Each mL contains the equivalent of 50 mg of elemental iron (as an iron dextran complex), approximately 0.9% sodium chloride, in water for injection. Sodium hydroxide and/or hydrochloric acid may have been used to adjust pH. The pH of the solution is between 5.2 and 6.5.

EPO (Retacrit®, Hospira, Inc), details from package insert: Epoetin alfa-epbx is a 165-amino acid erythropoiesis-stimulating glycoprotein manufactured by recombinant DNA technology. It has a molecular weight of approximately 30,400 daltons and is produced in Chinese Hamster Ovary (CHO) cell line. The product contains the identical amino acid sequence of isolated natural erythropoietin. RETACRIT (epoetin alfa-epbx) injection for intravenous or subcutaneous administration is a sterile, clear, colorless solution in single-dose vials, formulated with an isotonic sodium chloride/sodium phosphate buffered solution. Each 1 mL single-dose vial of 2,000, 3,000, 4,000, and 10,000 Units of epoetin alfa-epbx contains calcium chloride dihydrate (0.01 mg), glycine (7.5 mg), isoleucine (1 mg), leucine (1 mg), L-glutamic acid (0.25 mg), phenylalanine (0.5 mg), polysorbate 20 (0.1 mg), sodium chloride (2.4 mg), sodium phosphate dibasic anhydrous (4.9 mg), sodium phosphate monobasic monohydrate (1.3 mg), and threonine (0.25 mg), in Water for Injection, USP. Each 1 mL vial of 40,000 Units of epoetin alfa-epbx contains calcium chloride dihydrate (0.01 mg), glycine (7.5 mg), isoleucine (1 mg), leucine (1 mg), L-glutamic acid (0.25 mg), phenylalanine (0.5 mg), polysorbate 20 (0.1 mg), sodium chloride (2.2 mg), sodium phosphate dibasic anhydrous (5.7 mg), Reference ID: 4263015 sodium phosphate monobasic monohydrate (1.5 mg), and threonine (0.25 mg), in Water

6.2.3 PRODUCT STORAGE AND STABILITY

Iron (INFeD®): Store at 20°C to 25°C (68°F to 77°F); excursions permitted to 15°C to 30°C (59°F to 86°F).

EPO (Retacrit®): Store at 36°F to 46°F (2°C to 8°C). Do not freeze. Do not shake. Do not use RETACRIT that has been shaken or frozen. Store RETACRIT vials in the original carton until use to protect from light.

6.2.4 PREPARATION

No preparation (thawing, diluting, mixing, reconstitution) is required.

6.3 MEASURES TO MINIMIZE BIAS: RANDOMIZATION AND BLINDING

Participants will be randomized 1:1 to active intervention vs. standard of care using a stratified permuted block design by anemia etiology (iron responsive vs. not) and ICU admission indication (surgical vs. non-surgical). Randomization will occur electronically using the REDCap randomization module. Randomization lists will be developed by the study statisticians and uploaded directly to REDCap. Study staff involved in patient care are unable to access randomization lists in advance and therefore are unlikely to predict future randomization/assignment. Clinicians and subjects will not be blinded to study interventions. However, all data analysts will be blinded to treatment allocation while the study is ongoing.

6.4 STUDY INTERVENTION COMPLIANCE

Compliance will be assessed by the subject's receipt of pharmacologic therapy (iron +/- EPO). This will be assessed on study day 1 by research personnel. The proportion of subjects successfully receiving the intervention will be reported (number, %). Similarly, the proportion of subjects seen for 1-month and 3-month follow-up assessments will be reported.

6.5 CONCOMITANT THERAPY

For this protocol, a prescription medication is defined as a medication that can be prescribed only by a properly authorized/licensed clinician. Medications to be reported at baseline are all concomitant prescription medications, over-the-counter medications and supplements. Medications to be reported at daily visits are only to include if the subject has received iron or EPO/darbepoetin on these days.

Subjects who received iron therapies (exclusive or multivitamins with iron) or erythropoiesis stimulating agents (i.e. darbepoetin , erythropoietin) within 30 days will be excluded, as these subjects have already been receiving anemia treatment prior to enrollment.

6.5.1 RESCUE MEDICINE

Not applicable

7 STUDY INTERVENTION DISCONTINUATION AND PARTICIPANT DISCONTINUATION/WITHDRAWAL.

7.1 DISCONTINUATION OF STUDY INTERVENTION

Discontinuation from the pharmacologic therapies (i.e. iron, EPO) does not mean discontinuation from the study, and remaining study procedures should be completed as indicated by the study protocol. If a clinically significant finding is identified (including, but not limited to severe allergic reactions during medication administration) after enrollment, the investigator or qualified designee will determine if any change in participant management is needed. Any new clinically relevant finding will be reported as an adverse event (AE).

The data to be collected at the time of study intervention discontinuation will include the following:

- Reason for discontinuation
- Date and time of discontinuation
- Subject-initiated or investigator-initiated discontinuation
- Changes in vital signs or clinical status that prompted discontinuation
- Adverse events

7.2 PARTICIPANT DISCONTINUATION/WITHDRAWAL FROM THE STUDY

Participants are free to withdraw from participation in the study at any time upon request.
An investigator may discontinue or withdraw a participant from the study for the following reasons:

- Significant study intervention non-compliance
- If any clinical adverse event (AE), laboratory abnormality, or other medical condition or situation occurs such that continued participation in the study would not be in the best interest of the participant
- If the participant meets an exclusion criterion (either newly developed or not previously recognized) that precludes further study participation

The reason for participant discontinuation or withdrawal from the study will be recorded on the appropriate Case Report Form (CRF). Subjects who sign the informed consent form and are randomized but do not receive the study intervention may be replaced. Subjects who sign the informed consent form, and are randomized and receive the study intervention, and subsequently withdraw, or are withdrawn or discontinued from the study, will not be replaced.

7.3 LOST TO FOLLOW-UP

A participant will be considered lost to follow-up if they fail to return for 2 scheduled post-hospitalization visits and is unable to be contacted by the study site staff.

The following actions must be taken if a participant fails to return to the clinic for a required study visit:

- The site will attempt to contact the participant and reschedule the missed visit within 1 week and counsel the participant on the importance of maintaining the assigned visit schedule and ascertain if the participant wishes to and/or should continue in the study.
- Before a participant is deemed lost to follow-up, the investigator or designee will make every effort to regain contact with the participant (where possible, 3 telephone calls and, if necessary, a certified letter to the participant's last known mailing address or local equivalent methods). These contact attempts should be documented in the participant's medical record or study file.
- Should the participant continue to be unreachable, they will be considered to have withdrawn from the study with a primary reason of lost to follow-up.

8 STUDY ASSESSMENTS AND PROCEDURES

8.1 EFFICACY ASSESSMENTS

Study subjects will undergo the following procedures/evaluations:

- **Baseline assessments**. Medical history, surgical history, demographic features, height, weight, concomitant medication review and cumulative fluid balance, including previous exposure to blood transfusions, ADL survey.
- **Biological specimen collection and laboratory evaluations**. Following enrollment, study labs will be obtained according to table 1.3. CBC and creatinine is often done daily as standard of care. If the subject has a CBC and creatinine value obtained within the prior 24 hrs, this result may be used. However, if they received a unit of blood after that timepoint, the CBC would need to be repeated. All subjects will return for followup assessments and blood draws as noted in table 1.3. The research blood samples for storage and future research will be obtained (10 ml per subject) at enrollment and post-hospitalization assessments for future mechanistic studies. These samples will be cold-stored.
- **Administration of questionnaires or other instruments**. Subjects will undergo functional outcome assessments in accordance with the NHLBI-supported COMS of critical illness. These measures, either comprehensively or in part (as outlined in 1.3), will be obtained at enrollment, hospital discharge, and 1-month and 3-months post-hospitalization.

8.2 SAFETY AND OTHER ASSESSMENTS

Subjects will undergo the following procedures/evaluations:

- **Vital signs**. Temperature, pulse, respirations, blood pressure, continuous telemetry, oxygen saturations will be monitored during administration of iron and EPO for those in the intervention group as outlined in section 2.3. All subjects will receive standard ICU monitoring throughout their stay in the ICU.

- **Weight**. Subjects < 40 kg will be excluded due to unclear safety of 40,000 unit of EPO in persons with low weights

- **Assessment of adverse events.** Medical records will be reviewed daily for evaluation of adverse events. Adverse events will be indicated on the data forms for the study and on the specific adverse event report forms and all serious adverse events will be reported to the IRB within 24 hours of the research team learning about the event followed by a more detailed written report to the IRB. The following information about adverse events will be collected: 1) the onset and resolution of the event, 2) an assessment of the severity or intensity of the event, 3) an assessment of the relationship of the event to the intervention, and 4) any action taken because of event. The PI will report all potentially related SAEs to the DSMB and to NHLBI within 7 days of discovery.

8.3 ADVERSE EVENTS AND SERIOUS ADVERSE EVENTS

8.3.1 DEFINITION OF ADVERSE EVENTS (AE)

Adverse event means any untoward medical occurrence associated with the use of an intervention in humans, whether or not considered intervention-related (21 CFR 312.32 (a)).

As our subject population is by definition ‘critically ill’, it is expected that they will have many unrelated adverse health events during their hospital stay. Therefore, we will limit the scope of our adverse event monitoring and recording to focus on the following conditions:

- ➢ Study drug infusion reactions

- Venous thromboembolic disease
- Myocardial infarction
- Non-hemorrhagic stroke
- Bloodstream infections
- Hypertensive urgency or emergency in the 1 hour after study drug administration (i.e. SBP $\geq$ 200 or SBP increase by > 40 mmHg from the time of infusion start)

8.3.2 DEFINITION OF SERIOUS ADVERSE EVENTS (SAE)

An adverse event (AE) or suspected adverse reaction is considered "serious" if, in the view of either the investigator or sponsor, it results in any of the following outcomes: death, a life-threatening adverse event, inpatient hospitalization or prolongation of existing hospitalization, or a persistent or significant incapacity or substantial disruption of the ability to conduct normal life functions.

Serious adverse events (SAEs) will be defined as:

- Death, believed to be related to the study procedures, or a death that is unexpected considering the acuity of a subject.
- A life-threatening experience, including severe infusion reactions, believed to be related to the study procedures.
- Persistent or significant disability or incapacity that is of greater frequency or severity than what would be normally expected in the course of critical illness.
- An event that jeopardizes the human subject and may require medical or surgical treatment to prevent one of the preceding outcomes and is not expected in the subject's ICU course.

8.3.3 CLASSIFICATION OF AN ADVERSE EVENT

8.3.3.1 SEVERITY OF EVENT

For adverse events (AEs), the following guidelines will be used to describe severity.

- **Mild** – Events require minimal or no treatment and do not interfere with the participant's clinical course.
- **Moderate** – Events result in a low level of inconvenience or concern with the therapeutic measures. Moderate events may cause some interference with the subject's clinical course.
- **Severe** – Events interrupt a participant's clinical course and may require systemic drug therapy or other treatment. Severe events are usually potentially life-threatening or incapacitating.

8.3.3.2 RELATIONSHIP TO STUDY INTERVENTION

All adverse events (AEs) must have their relationship to study intervention assessed by the clinician who examines and evaluates the participant based on temporal relationship and his/her clinical judgment. The degree of certainty about causality will be graded using the categories below. In a clinical trial, the study product must always be suspect.

- **Definitely Related** – There is clear evidence to suggest a causal relationship, and other possible contributing factors can be ruled out. The clinical event occurs in a plausible time relationship to study intervention administration and cannot be explained by concurrent disease or other drugs or chemicals. The response to withdrawal of the study intervention (dechallenge) should be clinically plausible. The event must be

pharmacologically or phenomenologically definitive, with use of a satisfactory rechallenge procedure if necessary.

- **Probably Related** – There is evidence to suggest a causal relationship, and the influence of other factors is unlikely. The clinical event occurs within a reasonable time after administration of the study intervention, is unlikely to be attributed to concurrent disease or other drugs or chemicals, and follows a clinically reasonable response on withdrawal (dechallenge). Rechallenge information is not required to fulfill this definition.
- **Potentially Related** – There is some evidence to suggest a causal relationship (e.g., the event occurred within a reasonable time after administration of the trial medication). However, other factors may have contributed to the event (e.g., the participant's clinical condition, other concomitant events). Although an AE may rate only as "possibly related" soon after discovery, it can be flagged as requiring more information and later be upgraded to "probably related" or "definitely related", as appropriate.
- **Unlikely to be related** – A clinical event whose temporal relationship to study intervention administration makes a causal relationship improbable (e.g., the event did not occur within a reasonable time after administration of the study intervention) and in which other drugs or chemicals or underlying disease provides plausible explanations (e.g., the participant's clinical condition, other concomitant treatments).
- **Not Related** – The AE is completely independent of study intervention administration, and/or evidence exists that the event is definitely related to another etiology. There must be an alternative, definitive etiology documented by the clinician.

8.3.3.3 EXPECTEDNESS

The PI will be responsible for determining whether an adverse event (AE) is expected or unexpected. An AE will be considered unexpected if the nature, severity, or frequency of the event is not consistent with the risk information previously described for the study intervention.

8.3.4 TIME PERIOD AND FREQUENCY FOR EVENT ASSESSMENT AND FOLLOW-UP

The occurrence of an adverse event (AE) or serious adverse event (SAE) may come to the attention of study personnel during study visits and interviews of a study participant presenting for medical care, or upon review by a study monitor.

As noted earlier, for this study we plan to observe for a specific set of AEs and this information will be captured on the appropriate case report form (CRF). Information to be collected includes event description, time of onset, clinician's assessment of severity, relationship to study product (assessed only by those with the training and authority to make a diagnosis), and time of resolution/stabilization of the event. All AEs will be followed to adequate resolution.

Changes in the severity of an AE will be documented to allow an assessment of the duration of the event at each level of severity to be performed.

Study coordinators will record all listed reportable events with start dates occurring any time after informed consent is obtained through 3-months after hospitalization. At each study visit, the investigator will inquire about the occurrence of AE/SAEs since the last visit. Events will be followed for outcome information until resolution or stabilization.

8.3.5 ADVERSE EVENT REPORTING

All adverse events will be indicated on the data forms for the study and on the specific adverse event report forms and all serious adverse events will be reported to the IRB within 24 hours of the research team learning about the event followed by a more detailed written report to the IRB. The following information about adverse events will be collected: 1) the onset and resolution of the event, 2) an assessment of the severity or intensity of the event, 3) an

assessment of the relationship of the event to the intervention, and 4) any action taken because of event. The PI will report all potentially related SAEs to the NHLBI immediately after discovery (within 7 days).

8.3.6 SERIOUS ADVERSE EVENT REPORTING

The study clinician will immediately report to the IRB any serious adverse event, whether or not considered study intervention related, including those listed in the protocol or investigator brochure and must include an assessment of whether there is a reasonable possibility that the study intervention caused the event. Study endpoints that are serious adverse events (e.g., all-cause mortality) must be reported in accordance with the protocol unless there is evidence suggesting a causal relationship between the study intervention and the event (e.g., death from anaphylaxis). In that case, the investigator must immediately report the event to the sponsor. All serious adverse events (SAEs) will be followed until satisfactory resolution or until the site investigator deems the event to be chronic or the participant is stable.

8.3.7 REPORTING EVENTS TO PARTICIPANTS

Study coordinators will disclose any adverse events that are probably or definitively-related to the enrolled subject after review by the PI.

8.3.8 EVENTS OF SPECIAL INTEREST

Not applicable

8.3.9 REPORTING OF PREGNANCY

Not applicable

8.4 UNANTICIPATED PROBLEMS

8.4.1 DEFINITION OF UNANTICIPATED PROBLEMS (UP)

The Office for Human Research Protections (OHRP) considers unanticipated problems involving risks to participants or others to include, in general, any incident, experience, or outcome that meets **all** the following criteria:

- Unexpected in terms of nature, severity, or frequency given (a) the research procedures that are described in the protocol-related documents, such as the Institutional Review Board (IRB)-approved research protocol and informed consent document; and (b) the characteristics of the participant population being studied;
- Related or possibly related to participation in the research ("possibly related" means there is a reasonable possibility that the incident, experience, or outcome may have been caused by the procedures involved in the research); and
- Suggests that the research places participants or others at a greater risk of harm (including physical, psychological, economic, or social harm) than was previously known or recognized.

8.4.2 UNANTICIPATED PROBLEM REPORTING

The investigator will report unanticipated problems (UPs) to the reviewing Institutional Review Board (IRB) and to the principal investigator (PI). The UP report will include the following information:

- Protocol identifying information: protocol title and number, PI's name, and the IRB project number;
- A detailed description of the event, incident, experience, or outcome;
- An explanation of the basis for determining that the event, incident, experience, or outcome represents an UP;
- A description of any changes to the protocol or other corrective actions that have been taken or are proposed in response to the UP.

To satisfy the requirement for prompt reporting, UPs will be reported using the following timeline:

- UPs that are serious adverse events (SAEs) will be reported to the IRB within 7-days of the investigator becoming aware of the event.
- Any other UP will be reported to the IRB and to the DCC/study sponsor within 10-days of the investigator becoming aware of the problem.
- All UPs should be reported to appropriate institutional officials (as required by an institution's written reporting procedures), the supporting agency head (or designee), and the Office for Human Research Protections (OHRP) within 10 days of the IRB's receipt of the report of the problem from the investigator.

8.4.3 REPORTING UNANTICIPATED PROBLEMS TO PARTICIPANTS

Study coordinators will disclose UPs that are probably or definitively-related to the enrolled subject.

9 STATISTICAL CONSIDERATIONS

9.1 STATISTICAL HYPOTHESES

In this study, we will perform a pilot pragmatic clinical trial testing a multi-faceted anemia intervention (optimized phlebotomy practice, decision support, targeted pharmacologic anemia treatment) to attenuate anemia development and promote functional recovery in the setting of critical illness

Primary endpoint: To assess the impact of the intervention on 1 month post-hospitalization hemoglobin concentrations.

Secondary endpoint: To assess the impact of the intervention on post-hospitalization functional outcomes and hemoglobin concentrations measured at 3 months.

Hypotheses: A multifaceted anemia intervention will increase hemoglobin concentrations and will improve functional recovery through 3 months after hospitalization.

9.2 SAMPLE SIZE DETERMINATION

Power/sample size are calculated to detect a difference in the primary endpoint of hemoglobin at 1 month follow-up. Preliminary data show mean (standard deviation) hemoglobin levels of 10.8 (1.5) g/dL at 1 month among 636 subjects with similar inclusion/exclusion criteria receiving standard care. A total sample size of 74 (37 per group) provides 80% power to detect 1.0 g/dL improvement using a two-sample unequal variances t-test with 2-sided alpha of 0.05 to compare 1 month hemoglobin between randomized arms. Actual power is expected to be higher under the analysis approach, adjusting for pre-randomization prognostic variables to reduce residual variation. If adjustment variables account for 25% of the variation in 1 month hemoglobin (R^2 for relationship between pre-randomization adjustment variables and outcome = 0.25), the sample size of 37 per group provides 90% power to detect a 1.0 g/dL improvement using analysis of covariance with 2-sided alpha of 0.05. While the analysis uses a linear mixed-effects model (LMM), power is not appreciably different for the LMM as compared to the unequal variances t-test or ANCOVA described here.

Given expected dropout of up to 25% (death, loss to follow-up) resulting in loss of information, 100 subjects will be enrolled.

The study has not been powered for exploratory outcomes such as RBC transfusion, mortality, and readmissions, but this data will serve for hypothesis-generation for future, larger clinical trials.

9.3 POPULATIONS FOR ANALYSES

As the primary approach, subjects will be analyzed using an intention-to-treat (ITT) approach. Secondarily, analysis will be performed using modified intention-to-treat.

9.4 STATISTICAL ANALYSES

9.4.1 GENERAL APPROACH

Baseline demographic and clinical characteristics will be presented using descriptive statistics, as applicable. Unadjusted outcome summary statistics will be described for each randomized group. Continuous variables will be summarized by mean (standard deviation), median (25th, 75th) percentiles, and range. Categorical variables will be summarized by percentage.

The primary analysis dataset consists of all randomized participants and will be analyzed using intention-to-treat (ITT) principles such that each subject is analyzed based on their allocated arm. Mortality and dropout may occur, and subjects will be analyzed as described in 9.4.2 and 9.4.3.

A secondary analysis approach may consider a per-protocol analysis, analyzing those participants who completed study procedures without major protocol violations. The per-protocol analysis definition will be finalized prior to database lock and data analysis. The ITT analysis will be considered primary.

9.4.2 ANALYSIS OF THE PRIMARY EFFICACY ENDPOINT(S)

The primary outcome is hemoglobin measured repeatedly on subjects at ICU discharge, hospital discharge, and 1 month and 3 months. The longitudinal trajectory of hemoglobin will be analyzed with a LMM. The primary parameter of interest is a treatment group by time interaction to estimate the effect of treatment at each follow up time-point. The treatment effect on 1 month hemoglobin is the primary outcome; other time-points will reflect secondary outcomes. As the functional form of hemoglobin over time is unknown, a discrete time representation will be used. The model will adjust for pre-randomization hemoglobin, age, sex, anemia etiology, and medical vs surgical ICU setting to reduce residual variation and improve precision of the estimated treatment effect.

Dropout or non-response including skipped study visit and death represent two forms of missing data. Dropout or non-response unrelated to death at ICU discharge and hospital discharge is expected to be negligible but could occur with withdrawal of consent. We assume dropout (unrelated to death) while in the ICU is missing completely at random (MCAR); subjects withdrawing consent prior to observation of hemoglobin outcome at ICU discharge will be excluded from the analysis. Those dropping out after ICU discharge are assumed missing at random (MAR) with missingness possibly related to adjustment covariates, arm, or prior observed hemoglobin values. Analyses using LMM assume dropout, nonresponse, or skipped visits are MAR.

We anticipate up to 10% ICU mortality (despite exclusion of those not expected to survive hospitalization); such subjects will not have observed hemoglobin outcomes. We also anticipate additional post-ICU discharge mortality. In the primary analysis, we assume missing data due to death is MAR. Those with ICU mortality will have ICU discharge hemoglobin multiply imputed under the MAR assumption. Thereafter, LMMs assume additional missing data at other times are MAR. In secondary approaches to the analysis of hemoglobin, we use a worst-case imputation approach, to impute

hemoglobins as the worst possible outcome following death. After imputation, if residuals are not reasonably normally distributed, a generalized linear mixed effects proportional odds model will be used or individual timepoints may be assessed by Wilcoxon rank-sum test without covariate adjustment.

In the primary analysis, the point estimate, 95% confidence interval, and p-value will be reported from the LMM for the 1 month hemoglobin comparison. A point estimate in the direction favoring higher hemoglobin for the intervention arm and two-sided p-value<0.05 will reject the null hypothesis of no treatment benefit in favor of the conclusion that intervention improves 1 month hemoglobin.

9.4.3 ANALYSIS OF THE SECONDARY ENDPOINT(S)

Secondary and exploratory endpoints (besides hemoglobin at 3 months) include EQ-5D [Time frame: hospital discharge, 1 month, and 3 months post-hospitalization], PROMIS-Fatigue [Time frame: hospital discharge, 1 month, and 3 months post-hospitalization], 6-minute walk distance, activity monitoring (i.e. daily step counts, daily energy expenditure), and activities of daily living survey [Time frame: 1 and 3-months post-hospitalization], MoCA-BLIND [Time frame: 1 and 3-months post-hospitalization], HADS and IES-R [Time frame: 1 and 3-months post-hospitalization].

Activity Monitoring: During the initial consent process for the study, subjects will be given the option to also consent for activity monitoring at 1- and 3-months post-hospitalization. Activity monitors (AMs) are wearable devices (Actigraph) that will be provided to participants by mail. Participants will be asked to wear these monitors to measure physical activity and sedentary behavior in the free-living environment. The monitors will be worn over 4 consecutive days, which will occur during the 1- and 3-month assessment intervals. Devices will be distributed to patients either in-person during their 1- and 3-month appointments or delivered via mail. Participants will be given a pre-paid envelope to mail the activity monitors back after 4 consecutive days of wear. A valid AM hour is defined as ≤30 minutes of consecutive 'zero' values (no activity) and a valid AM day is defined as ≥10 wear hours per day. Subjects will be asked to repeat wearing the AM if a data collection contains ≤2 valid days across 4 days, or if the AM has malfunctioned. AMs are attached with straps worn on the non-dominant wrist. All activity monitors can be worn comfortably under or over clothing. The activity monitors are to be put on in the morning upon waking and removed at night before bed. The sensors are not waterproof and will be removed for bathing and swimming. The risks from wearing an AM are no different than those normally encountered in the free-living environment. Instructions for participants are provided in Appendix 3.

Analyses will use Wilcoxon rank-sum tests to compare the distribution of each outcome between groups at each timepoint since assumptions including normality of residuals are not expected to be satisfied for these outcomes. Additional, when applicable, a cutpoint defining a clinically actionable adverse outcome may be defined, for example, defining depression by HADS-Depression ≥8. Binary outcomes will be summarized as proportion and compared by randomized arm using a Chi-square test. Death and dropout will be assumed MAR in the primary approach and multiple imputation will be used to impute missing values. Analyses will be conducted on each imputed dataset and results combined to reflect uncertainty due to missing data. An additional analysis will consider a worst-value imputation for mortality, assigning death the worst possible outcome.

Mortality and readmission through 1 year post-discharge will be described by randomized arm using cumulative incidence estimates, censoring subjects at last known contact with the Mayo medical system when status is unknown at 1 year. Inferential analyses will use log-rank tests and Gray's test for mortality and readmission outcomes, respectively.

Since the goal is to provide robust data for further clinical trial evaluation, secondary and exploratory outcomes will be assessed without adjustment for multiplicity, with conclusions from each based on a two-sided alpha level 0.05 statistical test.

9.4.4 SAFETY ANALYSES

All AEs and SAEs will be presented and compared between groups. Mortality and hospital readmission rates will also be presented and compared between groups.

9.4.5 BASELINE DESCRIPTIVE STATISTICS

Descriptive statistics will be utilized to present baseline demographic, clinical, and laboratory features for both groups. No inferential statistics will be utilized.

9.4.6 PLANNED INTERIM ANALYSES

Not applicable

9.4.7 SUB-GROUP ANALYSES

We do not expect treatment effect heterogeneity of this intervention. However, exploratory analyses will assess potential for heterogeneity of treatment effect using interaction analyses in LMM models for the primary endpoint. An interaction term between randomized arm and each of sex, age, anemic etiology, and surgical vs. medical admissions, will be evaluated separately. The estimate of the treatment effect will be reported in subgroups using linear contrasts of with the interaction analysis when evidence supports a statistically significant interaction.

9.4.8 TABULATION OF INDIVIDUAL PARTICIPANT DATA

Not applicable.

9.4.9 EXPLORATORY ANALYSES

Multiple exploratory analyses have been planned as outlined throughout the protocol. These will be utilized to inform a definitive multicenter phase III clinical trial.

10 SUPPORTING DOCUMENTATION AND OPERATIONAL CONSIDERATIONS

10.1 REGULATORY, ETHICAL, AND STUDY OVERSIGHT CONSIDERATIONS

10.1.1 INFORMED CONSENT PROCESS

10.1.1.1 CONSENT/ASSENT AND OTHER INFORMATIONAL DOCUMENTS PROVIDED TO PARTICIPANTS

Consent forms describing in detail the study intervention, study procedures, and risks are given to the participant and written documentation of informed consent is required prior to starting intervention/administering study intervention. The following consent materials are submitted with this protocol: Consent form.

10.1.1.2 CONSENT PROCEDURES AND DOCUMENTATION

Informed consent is a process that is initiated prior to the individual's agreeing to participate in the study and continues throughout the individual's study participation. Consent forms will be Institutional Review Board (IRB)-approved and the

participant/LAR will be asked to read and review the document. The study coordinator will explain the research study to the participant &/or LAR and answer any questions that may arise. A verbal explanation will be provided in terms suited to the participant's comprehension of the purposes, procedures, and potential risks of the study and of their rights as research participants. Participants/LAR will have the opportunity to carefully review the written consent form and ask questions prior to signing. The participants should have the opportunity to discuss the study with their family or surrogates or think about it prior to agreeing to participate. The participant/LAR will sign the informed consent document prior to any procedures being done specifically for the study. Participants must be informed that participation is voluntary and that they may withdraw from the study at any time, without prejudice. A copy of the informed consent document will be given to the participants for their records. The informed consent process will be conducted and documented in the source document (including the date), and the form signed, before the participant undergoes any study-specific procedures. The rights and welfare of the participants will be protected by emphasizing to them that the quality of their medical care will not be adversely affected if they decline to participate in this study.

10.1.2 STUDY DISCONTINUATION AND CLOSURE

This study may be temporarily suspended or prematurely terminated if there is sufficient reasonable cause. Written notification, documenting the reason for study suspension or termination, will be provided by the suspending or terminating party to study participants, investigator, funding agency, and regulatory authorities. If the study is prematurely terminated or suspended, the PI will promptly inform study participants, IRB, and sponsor and will provide the reason(s) for the termination or suspension. Study participants will be contacted, as applicable, and be informed of changes to study visit schedule.

Circumstances that may warrant termination or suspension include, but are not limited to:

- Determination of unexpected, significant, or unacceptable risk to participants
- Demonstration of efficacy that would warrant stopping
- Insufficient compliance to protocol requirements
- Data that are not sufficiently complete and/or evaluable
- Determination that the primary endpoint has been met
- Determination of futility

Study may resume once concerns about safety, protocol compliance, and data quality are addressed, and satisfy the sponsor, IRB and/or Food and Drug Administration (FDA).

10.1.3 CONFIDENTIALITY AND PRIVACY

Participant confidentiality and privacy are strictly held in trust by the participating investigators, their staff, and the sponsor(s) and their interventions. This confidentiality is extended to cover testing of biological samples and genetic tests in addition to the clinical information relating to participants. Therefore, the study protocol, documentation, data, and all other information generated will be held in strict confidence. No information concerning the study or the data will be released to any unauthorized third party without prior written approval of the sponsor.

All research activities will be conducted in as private a setting as possible.

The study monitor, other authorized representatives of the sponsor, representatives of the IRB, regulatory agencies or pharmaceutical company supplying study product may inspect all documents and records required to be maintained by the investigator, including but not limited to, medical records (office, clinic, or hospital) and pharmacy records for the participants in this study. The clinical study site will permit access to such records.

The study participant's contact information will be securely stored at each clinical site for internal use during the study. At the end of the study, all records will continue to be kept in a secure location for as long a period as dictated by the reviewing IRB, Institutional policies, or sponsor requirements.

Study participant research data, which is for purposes of statistical analysis and scientific reporting, will be transmitted to and stored at the Mayo Clinic. This will not include the participant's contact or identifying information. Rather, individual participants and their research data will be identified by a unique study identification number. The study data entry and study management systems will be secured, and password protected. At the end of the study, all study databases will be de-identified and archived at the Mayo Clinic.

Certificate of Confidentiality

To further protect the privacy of study participants, a Certificate of Confidentiality will be issued by the National Institutes of Health (NIH). This certificate protects identifiable research information from forced disclosure. It allows the investigator and others who have access to research records to refuse to disclose identifying information on research participation in any civil, criminal, administrative, legislative, or other proceeding, whether at the federal, state, or local level. By protecting researchers and institutions from being compelled to disclose information that would identify research participants, Certificates of Confidentiality help achieve the research objectives and promote participation in studies by helping assure confidentiality and privacy to participants.

10.1.4 FUTURE USE OF STORED SPECIMENS AND DATA

With the participant's approval and as approved by local IRBs, de-identified biological samples will be stored at the Mayo Clinic with the same goal as the sharing of data with the Mayo Clinic. These samples could be used to research the causes of anemia, its complications and other conditions for which individuals with anemia are at increased risk, and to improve treatment. The PI and study team will keep a record that will allow linking the biological specimens with the phenotypic data from each participant, maintaining the blinding of the identity of the participant.

During the conduct of the study, an individual participant can choose to withdraw consent to have biological specimens stored for future research. However, withdrawal of consent with regard to biosample storage may not be possible after the study is completed.

When the study is completed, access to study data and/or samples will be provided through the PI and Mayo Clinic.

10.1.5 KEY ROLES AND STUDY GOVERNANCE

**Principal Investigator**
Matthew A. Warner, MD, Associate Professor of Anesthesiology
Mayo Clinic
200 1st St SW, Rochester, MN 55905
507-284-2511, #36558
warner.matthew@mayo.edu

10.1.6 SAFETY OVERSIGHT

Safety oversight will be conducted in accordance with the Data and Safety Monitoring Plan (DSMP).

10.1.7 CLINICAL MONITORING

Clinical site monitoring is conducted to ensure that the rights and well-being of trial participants are protected, that the reported trial data are accurate, complete, and verifiable, and that the conduct of the trial is in compliance with the currently approved protocol/amendment(s), with International Conference on Harmonisation Good Clinical Practice (ICH GCP), and with applicable regulatory requirement(s).

It is the responsibility of the Principal Investigator to oversee the safety of the study. This safety monitoring will include careful assessment and appropriate reporting of adverse events as noted above, as well as the construction and implementation of a site data and safety-monitoring plan. Medical monitoring will include a regular assessment of the number and type of serious adverse events

10.1.8 QUALITY ASSURANCE AND QUALITY CONTROL

The clinical site will perform internal quality management of study conduct, data and biological specimen collection, documentation and completion. An individualized quality management plan will be developed to describe a site's quality management.

Quality control (QC) procedures will be implemented beginning with the data entry system and data QC checks that will be run on the database will be generated. Any missing data or data anomalies will be communicated to the site(s) for clarification/resolution.

Following written Standard Operating Procedures (SOPs), the monitors will verify that the clinical trial is conducted and data are generated and biological specimens are collected, documented (recorded), and reported in compliance with the protocol, International Conference on Harmonisation Good Clinical Practice (ICH GCP), and applicable regulatory requirements (e.g., Good Laboratory Practices (GLP), Good Manufacturing Practices (GMP)).

The investigational site will provide direct access to all trial related sites, source data/documents, and reports for the purpose of monitoring and auditing by the sponsor, and inspection by local and regulatory authorities.

10.1.9 DATA HANDLING AND RECORD KEEPING

10.1.9.1 DATA COLLECTION AND MANAGEMENT RESPONSIBILITIES

Data collection is the responsibility of the clinical trial staff at the site under the supervision of the site investigator. The investigator is responsible for ensuring the accuracy, completeness, legibility, and timeliness of the data reported. All source documents should be completed in a neat, legible manner to ensure accurate interpretation of data. Hardcopies of the study visit worksheets will be provided for use as source document worksheets for recording data for each participant enrolled in the study. Data collected and entered into electronic case report form (eCRF) derived from source documents should be consistent with the data recorded on the source documents. Clinical data (including adverse events (AEs), concomitant medications, and expected adverse reactions data) and clinical laboratory data will be entered into REDCap electronic data capture. REDCap is HIPAA-compliant with built-in user right controls and audit trails for data security and tracking. The data system includes password protection and internal quality checks, such as automatic range checks, to identify data that appear inconsistent, incomplete, or inaccurate. Clinical data will be entered directly from the source documents.

10.1.9.2 STUDY RECORDS RETENTION

Study documents should be retained for a minimum of 2 years. These documents should be retained for a longer period, however, if required by local regulations. No records will be destroyed without the written consent of the sponsor, if applicable. It is the responsibility of the sponsor to inform the investigator when these documents no longer need to be retained.

10.1.10 PROTOCOL DEVIATIONS

A protocol deviation is any noncompliance with the clinical trial protocol, International Conference on Harmonisation Good Clinical Practice (ICH GCP), or Manual of Procedures (MOP) requirements. The noncompliance may be either on the part of the participant, the investigator, or the study site staff. As a result of deviations, corrective actions are to be developed by the site and implemented promptly.

These practices are consistent with ICH GCP:

- 4.5 Compliance with Protocol, sections 4.5.1, 4.5.2, and 4.5.3
- 5.1 Quality Assurance and Quality Control, section 5.1.1
- 5.20 Noncompliance, sections 5.20.1, and 5.20.2.

It is the responsibility of the site investigator to use continuous vigilance to identify and report deviations. All deviations must be addressed in study source documents, reported to the NHLBI Program Official. Protocol deviations must be sent to the reviewing Institutional Review Board (IRB) per their policies. The site investigator is responsible for knowing and adhering to the reviewing IRB requirements.

10.1.11 PUBLICATION AND DATA SHARING POLICY

This study will be conducted in accordance with the following publication and data sharing policies and regulations:

National Institutes of Health (NIH) Public Access Policy, which ensures that the public has access to the published results of NIH funded research. It requires scientists to submit final peer-reviewed journal manuscripts that arise from NIH funds to the digital archive PubMed Central upon acceptance for publication.

This study will comply with the NIH Data Sharing Policy and Policy on the Dissemination of NIH-Funded Clinical Trial Information and the Clinical Trials Registration and Results Information Submission rule. As such, this trial will be registered at ClinicalTrials.gov, and results information from this trial will be submitted to ClinicalTrials.gov. In addition, every attempt will be made to publish results in peer-reviewed journals. Data from this study may be requested from other researchers within 5 years after the completion of the primary endpoint by contacting the PI.

10.1.12 CONFLICT OF INTEREST POLICY

The independence of this study from any actual or perceived influence, such as by the pharmaceutical industry, is critical. Therefore, any actual conflict of interest of persons who have a role in the design, conduct, analysis, publication, or any aspect of this trial will be disclosed and managed. Furthermore, persons who have a perceived conflict of interest will be required to have such conflicts managed in a way that is appropriate to their participation in the design and conduct of this trial. The study leadership in conjunction with NHLBI has established policies and procedures for all study group members to disclose all conflicts of interest and will establish a mechanism for the management of all reported dualities of interest.

10.2 ADDITIONAL CONSIDERATIONS

Appendix 1: Visual Aid

Anemia Study Participant

Anemia is common in ICU patients and is associated with adverse outcomes during and after hospitalization. The goal of this clinical trial is to mitigate anemia severity and treat anemia in critically ill patients.

_________________ is enrolled in the active arm of this study, which consists of:

1. *Optimized Phlebotomy*: micro blood draws, closed loop blood sampling, lab bundling
2. *Pharmacological Therapy*: IV iron +/- EPO (single dose at enrollment)
3. *Clinician Engagement & Decision Support*: visual aid, daily communications (Epic, in-person)

What are we asking from you? Continue to provide routine care for patients not in the active study arm (i.e. do **NOT** adjust practice). For patients in the active arm:

- **Remove invasive lines as soon as possible**
  - Arterial and central venous catheters should be removed when no longer required.
  - Do NOT draw cultures from the line that is being removed.
- **Eliminate/minimize all non-essential laboratory draws**
  - Do NOT schedule daily labs. Assess needs daily.
  - Avoid ABGs to assess PaO2 (SpO2 usually sufficient) or after modifying ventilator settings.
  - Avoid ABGs to assess PaCO2 in patients with clear mentation.
  - Avoid serial lactates in patients that are improving.
  - Avoid frequent sodium checks for hyponatremia in absence of hypertonic saline therapy.
  - Avoid serial coagulation tests as postoperative routine (e.g. Q6H INR, APTT, fibrinogen).
- **Consider lab holidays**
  - No daily CBC unless concern for bleeding or new/worsening infection.
  - No daily electrolytes unless requiring active management (e.g. large-volume diuresis).
- **Bundle labs to prevent multiple phlebotomy episodes**
  - If new lab required, order as "add-on".
  - If "add-on" not possible, please schedule the lab to be drawn when others will be drawn.
- **Avoid blood cultures in those with low pre-test probability of bacteremia**
  - Isolated fever and/or leukocytosis
  - Postop fever < 48 hours from surgery
  - Non-severe infections (i.e. CAP, HAP, cellulitis, cystitis)
- **When blood cultures are indicated, do NOT culture all lines**
  - Only culture from one line + one peripheral stick (2 total cultures)
- **Ensure adequate nutrition**
  - Adequate nutrition is important for erythropoiesis
  - Enteral tubes (NG) may cause mucosal erosion and bleeding; remove when no longer required

Appendix 2: Daily Rounding

Daily rounding checklist

- ☐ *Assess suitability for removal of invasive lines* (arterial line, central line)

- ☐ *Review all scheduled laboratory orders*
  - ☐ Does this patient *really* need labs tomorrow? Consider lab holiday
  - ☐ Ensure essential labs are being drawn together (align scheduling)
  - ☐ Avoid routine scheduling of labs (i.e. daily CBC, electrolytes)

- ☐ *Review the necessity for additional laboratory orders*
  - ☐ Does this patient *really* need blood cultures?
    Do NOT order reflexively for those with low pre-test probability of bacteremia (postop fever < 48 hours from surgery, isolated fever and/or leukocytosis, non-severe infections).
    Do NOT cultures every line. Only culture from 1 invasive line + 1 peripheral stick.
  - ☐ Does this patient *really* need an ABG?
    Do I really need to trend the PaO2? Can I simply assess oxygenation with the SpO2?
    If the patient is lucid/stable, do I really care what the PaCO2 is?
  - ☐ Do I need to repeat the lactate if the patient is improving?

- ☐ *Discontinue unnecessary intravenous fluids*
  Excessive IV fluids cause dilutional anemia (i.e. drop in hemoglobin concentration without any change in the actual red cell mass). Maintenance fluids discouraged for patients able to tolerate oral intake.

- ☐ *Ensure adequate nutrition*
  - ☐ Assess suitability for oral diet / dietary advancement
  - ☐ Assess suitability for removal of enteral lines (NG tube)

- ☐ *Ensure appropriate GI stress ulcer prophylaxis*
  (MV > 48 hours, coagulopathy, recent history of GI bleeding, TBI or traumatic SC injury, severe burns; at least 2 of the following: high dose steroids, occult bleeding ≥ 6 days, ICU stay ≥ 7 days, sepsis).

Appendix 3: Activity monitor instructions

When to wear the activity monitor:
(the activity monitor will be worn for 4 consecutive days)

Start day____________________________

End day_____________________________

Wear the monitor during waking hours only for the consecutive days specified above. They are not waterproof, so please remove them during showering, swimming, or any other activity that would submerge them in water and put them back on after you have completed that activity.

You **do not** need to alter any of your normal activities during the days of data collection, and you **do not** need to keep track of your activities. We want you to do all your normal activities to the best of your ability.

General Information

The activity monitor will arrive encased in the wrist band with which it is to be used in. It will additionally arrive already turned "on", and there will be no need to turn them "off" or "on". A blinking light may be visible from the activity monitor and should not cause alarm.

<u>Putting the Activity Monitors ON</u>

The activity monitor will be worn on the non-dominant wrist on an elastic wrist band. **The monitor can be worn over or under clothes, whichever is most comfortable for you**.

Attach the strap as shown in Figure 1. The strap should be tightened around the wrist so it is comfortable but will not move around during activities. If there is any shifting or loosening of the wrist band throughout the day, you should reposition and/or retighten the strap. Please do not remove the sensor from the wrist band at any time.

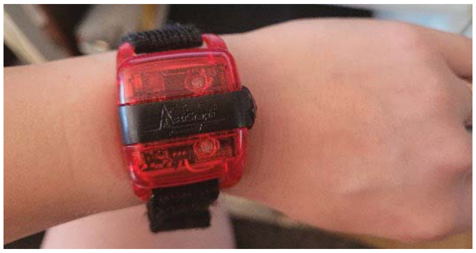

Figure 1. Activity monitor placement for wrist.

If you have any questions, call or email XXXXX at XXXXX or *XXXX@mayo.edu*.

10.3 ABBREVIATIONS

ADL	Activities of Daily Living
AE	Adverse Event
CFR	Code of Federal Regulations
CLIA	Clinical Laboratory Improvement Amendments
CMP	Clinical Monitoring Plan
COC	Certificate of Confidentiality
COMS	Core Outcome Measurement Set
CONSORT	Consolidated Standards of Reporting Trials
CRF	Case Report Form
DCC	Data Coordinating Center
DHHS	Department of Health and Human Services
DSMP	Data Safety Monitoring Plan
DRE	Disease-Related Event
EC	Ethics Committee
eCRF	Electronic Case Report Forms
EHR	Electronic health record
EPO	Erythropoietin
ESA	Erythropoiesis-stimulating agents
FDA	Food and Drug Administration
FDAAA	Food and Drug Administration Amendments Act of 2007
FFR	Federal Financial Report
GCP	Good Clinical Practice
GLP	Good Laboratory Practices
GMP	Good Manufacturing Practices
HIPAA	Health Insurance Portability and Accountability Act
IB	Investigator's Brochure
ICH	International Conference on Harmonisation
ICMJE	International Committee of Medical Journal Editors
IDE	Investigational Device Exemption
IND	Investigational New Drug Application
IRB	Institutional Review Board
ISM	Independent Safety Monitor
ISO	International Organization for Standardization
ITT	Intention-To-Treat
IV	Intravenous
MedDRA	Medical Dictionary for Regulatory Activities
MOP	Manual of Procedures
MSDS	Material Safety Data Sheet
NCT	National Clinical Trial
NIH	National Institutes of Health
NIH IC	NIH Institute or Center
OHRP	Office for Human Research Protections
PI	Principal Investigator
QA	Quality Assurance
QC	Quality Control
SAE	Serious Adverse Event
SAP	Statistical Analysis Plan
SMC	Safety Monitoring Committee
SOA	Schedule of Activities
SOC	System Organ Class

SOP	Standard Operating Procedure
UP	Unanticipated Problem
US	United States
6MWD	6-minute walk distance

11 REFERENCES

1. Walsh TS, Saleh E-E-D. Anaemia during critical illness. *Br J Anaesth*. 2006;97(3):278-291. doi:10.1093/bja/ael189
2. Thomas J, Jensen L, Nahirniak S, Gibney RTN. Anemia and blood transfusion practices in the critically ill: A prospective cohort review. *Hear Lung J Acute Crit Care*. 2010;39(3):217-225. doi:10.1016/j.hrtlng.2009.07.002
3. Warner MA, Hanson AC, Frank RD, et al. Prevalence and Recovery from Anemia Following Hospitalization for Critical Illness among Adults in Minnesota, 2010-2016. *JAMA Netw Open*. 2020;[Accepted.
4. Hébert PC, Wells G, Blajchman MA, et al. A Multicenter, Randomized, Controlled Clinical Trial of Transfusion Requirements in Critical Care. *N Engl J Med*. 1999;340(6):409-417. doi:10.1056/NEJM199902113400601
5. Needham DM, Davidson J, Cohen H, et al. Improving long-term outcomes after discharge from intensive care unit. *Crit Care Med*. 2012;40(2):502-509. doi:10.1097/CCM.0b013e318232da75
6. Wu Z, McGoogan JM. Characteristics of and Important Lessons from the Coronavirus Disease 2019 (COVID-19) Outbreak in China: Summary of a Report of 72314 Cases from the Chinese Center for Disease Control and Prevention. *JAMA - J Am Med Assoc*. 2020;323(13):1239-1242. doi:10.1001/jama.2020.2648
7. Warner MA, Kor DJ, Frank RD, et al. Anemia in Critically Ill Patients With Acute Respiratory Distress Syndrome and Posthospitalization Physical Outcomes. *J Intensive Care Med*. 1(9). doi:10.1177/0885066620913262
8. Den Elzen WPJ, Willems JM, Westendorp RGJ, De Craen AJM, Assendelft WJJ, Gussekloo J. Effect of anemia and comorbidity on functional status and mortality in old age: Results from the Leiden 85-plus Study. *Cmaj*. 2009;181(3-4):151-157. doi:10.1503/cmaj.090040
9. Denny SD, Kuchibhatla MN, Cohen HJ. Impact of anemia on mortality, cognition, and function in community-dwelling elderly. *Am J Med*. 2006;119(4):327-334. doi:10.1016/j.amjmed.2005.08.027
10. Bandinelli S, Ferrucci L, Corsi AM, et al. Anemia Is Associated with Disability and Decreased Physical Performance and Muscle Strength in the Elderly. *J Am Geriatr Soc*. 2004;52(5):719-724. doi:10.1111/j.1532-5415.2004.52208.x
11. Thein M, Ershler WB, Artz AS, et al. Diminished quality of life and physical function in community-dwelling elderly with anemia. *Medicine (Baltimore)*. 2009;88(2):107-114. doi:10.1097/MD.0b013e31819d89d5
12. Conlon NP, Bale EP, Herbison GP, McCarroll M. Postoperative Anemia and Quality of Life After Primary Hip Arthroplasty in Patients Over 65 Years Old. *Anesth Analg*. 2008;106(4):1056-1061. doi:10.1213/ane.0b013e318164f114
13. Lawrence VA, Silverstein JH, Cornell JE, Pederson T, Noveck H, Carson JL. Higher Hb level is associated with better early functional recovery after hip fracture repair. *Transfusion*. 2003;43(12):1717-1722. doi:10.1046/j.0041-1132.2003.00581.x
14. Foss NB, Kristensen MT, Kehlet H. Anaemia impedes functional mobility after hip fracture surgery. *Age Ageing*. 2008;37(2):173-178. doi:10.1093/ageing/afm161
15. Fukushima T, Nakano J, Ishii S, et al. Influence of Hemoglobin Level on Muscle and Physical Functions, Activities of Daily Living, and Quality of Life in Patients With Hematological Malignancies. *Integr Cancer Ther*. 2019;18:1-7. doi:10.1177/1534735419842196
16. Morishita S, Kaida K, Yamauchi S, et al. Relationship between corticosteroid dose and declines in physical function among allogeneic hematopoietic stem cell transplantation patients. *Support Care Cancer*. 2013;21(8):2161-2169. doi:10.1007/s00520-013-1778-7
17. Peuranp € A € A P, Heli S, Ovaara-Peippo €, Fraser I, Paavonen J, Ritva Hurskainen &. Effects of anemia and iron deficiency on quality of life in women with heavy menstrual bleeding. doi:10.1111/aogs.12394
18. Roubinian NH, Murphy EL, Mark DG, et al. Long-Term Outcomes Among Patients Discharged From the Hospital With Moderate Anemia. *Ann Intern Med*. 2019;170(2):81. doi:10.7326/M17-3253
19. Docherty AB, Turgeon AF, Walsh TS. Best practice in critical care: anaemia in acute and critical illness. *Transfus Med*. 2018;28(2):181-189. doi:10.1111/tme.12505
20. Bateman AP, McArdle F, Walsh TS. Time course of anemia during six months follow up following intensive care discharge and factors associated with impaired recovery of erythropoiesis. *Crit Care Med*. 2009;37(6):1906-1912. doi:10.1097/CCM.0b013e3181a000cf
21. St Sauver JL, Grossardt BR, Finney Rutten LJ, et al. Rochester Epidemiology Project Data Exploration Portal. *Prev*

Chronic Dis. 2018;15:E42. doi:10.5888/pcd15.170242

22. Rocca WA, Yawn BP, St. Sauver JL, Grossardt BR, Melton LJ. History of the Rochester Epidemiology Project: Half a Century of Medical Records Linkage in a US Population. *Mayo Clin Proc*. 2012;87(12):1202-1213. doi:10.1016/J.MAYOCP.2012.08.012
23. Needham DM, Sepulveda KA, Dinglas VD, et al. Core outcome measures for clinical research in acute respiratory failure survivors an international modified delphi consensus study. *Am J Respir Crit Care Med*. 2017;196(9):1122-1130. doi:10.1164/rccm.201702-0372OC
24. Wu Y, Spaulding AC, Borkar S, et al. Reducing Blood Loss by Changing to Small Volume Tubes for Laboratory Testing. *Mayo Clin Proc Innov Qual Outcomes*. 2021;5(1):72-83. doi:10.1016/J.MAYOCPIQO.2020.08.007
25. Siegal DM, Belley-Cote E, Arnold DM, et al. Small-Volume Tubes to Reduce Anemia and Transfusion (STRATUS): A Pragmatic Stepped Wedge Cluster Randomized Trial. *Blood*. 2019;134(Supplement_1):3685-3685. doi:10.1182/BLOOD-2019-130257
26. Barreda Garcia J, Xian JZ, Pedroza C, et al. Pediatric size phlebotomy tubes and transfusions in adult critically ill patients: A pilot randomized controlled trial. *Pilot Feasibility Stud*. 2020;6(1):1-9. doi:10.1186/s40814-020-00657-3
27. Corwin HL, Gettinger A, Pearl RG, et al. Efficacy of Recombinant Human Erythropoietin in Critically Ill Patients. *JAMA*. 2002;288(22):2827. doi:10.1001/jama.288.22.2827
28. Corwin HL, Gettinger A, Fabian TC, et al. Efficacy and Safety of Epoetin Alfa in Critically Ill Patients. *N Engl J Med*. 2007;357(10):965-976. doi:10.1056/NEJMoa071533
29. Corwin HL, Gettinger A, Rodriguez RM, et al. Efficacy of Recombinant Human Erythropoietin in the Critically Ill Patient. *Crit Care Med*. 1998;26(Supplement):23A. doi:10.1097/00003246-199801001-00007
30. Shah A, Fisher SA, Wong H, et al. Safety and efficacy of iron therapy on reducing red blood cell transfusion requirements and treating anaemia in critically ill adults: A systematic review with meta-analysis and trial sequential analysis. *J Crit Care*. 2019;49:162-171. doi:10.1016/j.jcrc.2018.11.005
31. Lasocki S, Asfar P, Jaber S, et al. Impact of treating iron deficiency, diagnosed according to hepcidin quantification, on outcomes after a prolonged ICU stay compared to standard care: a multicenter, randomized, single-blinded trial. *Crit Care*. 2021;25(1):1-10. doi:10.1186/s13054-020-03430-3
32. The IRONMAN Investigators, Litton E, Baker S, et al. Intravenous iron or placebo for anaemia in intensive care: the IRONMAN multicentre randomized blinded trial: A randomized trial of IV iron in critical illness. *Intensive Care Med*. 2016;42(11):1715-1722. doi:10.1007/s00134-016-4465-6
33. Richards T, Clevenger B, Keidan J, et al. PREVENTT: Preoperative intravenous iron to treat anaemia in major surgery: Study protocol for a randomised controlled trial. *Trials*. 2015;16(1). doi:10.1186/s13063-015-0774-2
34. Chertow GM, Mason PD, Vaage-Nilsen O, Ahlme J. Update on adverse drug events associated with parenteral iron. *Nephrol Dial Transpl*. 2006;21:378-382. doi:10.1093/ndt/gfi253
35. Litton E, Latham P, Inman J, Luo J, Allan P. Safety and efficacy of erythropoiesis-stimulating agents in critically ill patients admitted to the intensive care unit: a systematic review and meta-analysis. *Intensive Care Med*. 2019;45(9):1190-1199. doi:10.1007/s00134-019-05686-y
36. M H, R L, C R, et al. Erythropoietin to treat head and neck cancer patients with anaemia undergoing radiotherapy: randomised, double-blind, placebo-controlled trial. *Lancet (London, England)*. 2003;362(9392):1255-1260. doi:10.1016/S0140-6736(03)14567-9
37. JR W, YC U, JA J, et al. Randomized, double-blind, placebo-controlled trial of erythropoietin in non-small-cell lung cancer with disease-related anemia. *J Clin Oncol*. 2007;25(9):1027-1032. doi:10.1200/JCO.2006.07.1514
38. B L-J, V S, M P, et al. Maintaining normal hemoglobin levels with epoetin alfa in mainly nonanemic patients with metastatic breast cancer receiving first-line chemotherapy: a survival study. *J Clin Oncol*. 2005;23(25):5960-5972. doi:10.1200/JCO.2005.06.150
39. Choi MJ, Yee J. Erythropoiesis-Stimulating Agents and Cancer: Myth or Truth. Published online 2019. doi:10.1053/j.ackd.2019.04.001
40. J B, K B, R C, et al. Management of Cancer-Associated Anemia With Erythropoiesis-Stimulating Agents: ASCO/ASH Clinical Practice Guideline Update. *J Clin Oncol*. 2019;37(15):1336-1351. doi:10.1200/JCO.18.02142
41. Warner MA, Shore-Lesserson L, Shander A, Patel SY, Perelman SI, Guinn NR. Perioperative Anemia. *Anesth Analg*.

Published online March 2020:1. doi:10.1213/ANE.0000000000004727

42. Litton E, Latham P, Inman J, Luo J, Allan P. Safety and efficacy of erythropoiesis-stimulating agents in critically ill patients admitted to the intensive care unit: a systematic review and meta-analysis. *Intensive Care Med*. 2019;45(9):1190-1199. doi:10.1007/s00134-019-05686-y
43. Lasocki S, Puy H, Mercier G, et al. Impact of iron deficiency diagnosis using hepcidin mass spectrometry dosage methods on hospital stay and costs after a prolonged ICU stay: Study protocol for a multicentre, randomised, single-blinded medico-economic trial. *Anaesth Crit Care Pain Med*. 2017;36(6):391-396. doi:10.1016/j.accpm.2017.04.009
44. Pieracci FM, Barie PS. Iron and the Risk of Infection. *Surg Infect (Larchmt)*. 2005;6(s1):s41-s46. doi:10.1089/sur.2005.6.s1-41
45. Geisser P, Burckhardt S. The pharmacokinetics and pharmacodynamics of iron preparations. *Pharmaceutics*. 2011;3(1):12-33. doi:10.3390/pharmaceutics3010012
46. Warner MA, Shore-Lesserson L, Shander A, Patel SY, Perelman SI, Guinn NR. Perioperative Anemia. *Anesth Analg*. Published online March 2020:1. doi:10.1213/ane.0000000000004727
47. Spahn DR, Schoenrath F, Spahn GH, et al. Effect of ultra-short-term treatment of patients with iron deficiency or anaemia undergoing cardiac surgery: a prospective randomised trial. *Lancet*. 2019;6736(18):1-12. doi:10.1016/S0140-6736(18)32555-8
48. Pieracci FM, Henderson P, Rodney JRM, et al. Randomized, Double-Blind, Placebo-Controlled Trial of Effects of Enteral Iron Supplementation on Anemia and Risk of Infection during Surgical Critical Illness. *Surg Infect (Larchmt)*. 2009;10(1):9-19. doi:10.1089/sur.2008.043
49. Pieracci FM, Stovall RT, Jaouen B, et al. A multicenter, randomized clinical trial of IV iron supplementation for anemia of traumatic critical illness*. *Crit Care Med*. 2014;42(9):2048-2057. doi:10.1097/CCM.0000000000000408
50. Dinglas VD, Faraone LN, Needham DM. Understanding patient-important outcomes after critical illness. *Curr Opin Crit Care*. 2018;24(5):401-409. doi:10.1097/MCC.0000000000000533
51. Roberts RO, Geda YE, Knopman DS, et al. The Mayo Clinic Study of Aging: Design and sampling, participation, baseline measures and sample characteristics. *Neuroepidemiology*. 2008;30(1):58-69. doi:10.1159/000115751

Statistical Analysis Plan

Sample size estimation:

Power/sample size are calculated to detect a difference in hemoglobin at 1 month follow-up. Preliminary data show mean (standard deviation) hemoglobin levels of 10.8 (1.5) g/dL at 1 month among 636 patients with similar inclusion/exclusion criteria receiving standard care. A total sample size of 74 (37 per group) provides 80% power to detect 1.0 g/dL difference using a two-sample unequal variances t-test with 2-sided alpha of 0.05 to compare 1 month hemoglobin between randomized arms. Actual power is expected to be higher adjusting for pre-randomization prognostic variables to reduce residual variation. If adjustment variables account for 25% of the variation in 1 month hemoglobin, the sample size of 37 per group provides 90% power to detect 1.0 g/dL difference using analysis of covariance. While the analysis will employ a linear mixed-effects model (LMM), power is not appreciably different. Given expected dropout of up to 25% (death, loss to follow-up), 100 subjects will be enrolled.

Statistical considerations:

As the primary approach, subjects will be analyzed using an intention-to-treat (ITT) approach, including all patients randomized and analyzed by their assigned arm. Secondarily, analyses may be performed using modified intention-to-treat (mITT), allowing exclusion of patients who withdraw consent prior to ICU discharge as this is unlikely to be related to assigned study arm. The longitudinal trajectory of hemoglobin will be analyzed with a LMM. The primary parameter of interest is a treatment group by time interaction to estimate the effect of treatment at each follow up time-point, with 1 month hemoglobin serving as the primary outcome. The model will

adjust for pre-randomization hemoglobin, age, sex, anemia etiology, and medical vs surgical ICU setting to reduce residual variation and improve precision.

Dropout or non-response including skipped study visit and death represent two forms of missing data. We assume dropout (unrelated to death) while in the ICU is missing completely at random (MCAR); patients withdrawing consent prior to observation of hemoglobin outcome at ICU discharge will be excluded from mITT analyes. Those dropping out after ICU discharge are assumed missing at random (MAR) with missingness possibly related to adjustment covariates, arm, or prior observed hemoglobin values. We anticipate up to 10% ICU mortality despite exclusion of those not expected to survive hospitalization; such subjects will not have observed hemoglobin outcomes. We also anticipate post-ICU discharge mortality. Those with ICU mortality will have ICU discharge hemoglobin multiply imputed under the MAR assumption. Thereafter, LMMs assume additional missing data at other times are MAR. In secondary approaches to the analysis of hemoglobin, we will consider a worst-case imputation approach assigning a value of 0 g/dL following death. If residuals are not reasonably normally distributed, a generalized linear mixed effects proportional odds model will be used or individual timepoints may be assessed by Wilcoxon rank-sum test without covariate adjustment.

Similar analytical approaches using proportional odds models or Wilcoxon rank-sum test will apply to the assessment of functional outcomes which are not expected to satisfy regression assumptions including normality of residuals, with additional adjustment for baseline ADLs for physical outcomes. When applicable, a cutpoint defining a clinically actionable adverse outcome may be defined (e.g., depression as HADS-Depression ≥8). Binary outcomes will be summarized as proportion and compared by randomized arm using a Chi-square test. Mortality and readmission through 1-year post-discharge will be described by randomized arm using

cumulative incidence estimates, censoring subjects at last known contact with the healthcare system when status is unknown at 1 year. Inferential analyses will use log-rank tests and Gray's test for mortality and readmission outcomes, respectively.

We do not expect treatment effect heterogeneity of this intervention. However, exploratory analyses will assess potential for heterogeneity of treatment effect using interaction analyses in LMM models for the primary endpoint. An interaction term between randomized arm and each of sex, age, anemia etiology, and surgical vs. medical admissions, will be evaluated separately. The estimate of the treatment effect will be reported in subgroups using linear contrasts with the interaction analysis when evidence supports a statistically significant interaction.

In the primary analysis, the point estimate, 95% confidence interval, and p-value will be reported from the LMM for the 1-month hemoglobin comparison. Since the goal is to provide robust data for further clinical trial evaluation, secondary and exploratory outcomes will be assessed without adjustment for multiplicity, with conclusions from each based on a two-sided alpha level 0.05 statistical test. There are no planned interim analyses.
