# Supplementary material for: Practical Anemia Bundle and Hemoglobin Recovery in Critical Illness: A Randomized Clinical Trial
Source: JAMA Netw Open. 2025 Mar 28;8(3):e252353. doi: 10.1001/jamanetworkopen.2025.2353 (PMC11953759; doi:10.1001/jamanetworkopen.2025.2353)
Supplement: Supplement 2. — eTable 1. Additional Patient and Hospitalization Characteristics eTable 2. Demographic, Clinical, and Laboratory Features for Those With and Without 1-Month Post-Hospitalization Hemoglobin Assessments eTable 3. Estimated Treatment Effect for Hemoglobin Using Worst-Case Imputation (No Change From Baseline) Approach eTable 4. Adverse Events eTable 5. Phlebotomy Summaries eTable 6. EQ-5D-3L Domain Scores eTable 7. Interaction Analyses eFigure 1. Hemoglobin Values Over Time for Individual Participants and Study Groups eFigure 2. 6-Minute Walk Distances at 1- and 3-Months [file jamanetwopen-e252353-s002.pdf]

Supplemental Online Content

Warner MA, Johnson ML, Hanson AC, et al. Practical anemia bundle and hemoglobin recovery in critical illness: a randomized clinical trial. *JAMA Netw Open*. Published online March 28, 2025. doi:10.1001/jamanetworkopen.2025.2353

eTable 1. Additional Patient and Hospitalization Characteristics

eTable 2. Demographic, Clinical, and Laboratory Features for Those With and Without 1-Month Post-Hospitalization Hemoglobin Assessments

eTable 3. Estimated Treatment Effect for Hemoglobin Using Worst-Case Imputation (No Change From Baseline) Approach

eTable 4. Adverse Events

eTable 5. Phlebotomy Summaries

eTable 6. EQ-5D-3L Domain Scores

eTable 7. Interaction Analyses

eFigure 1. Hemoglobin Values Over Time for Individual Participants and Study Groups

eFigure 2. 6-Minute Walk Distances at 1- and 3-Months

This supplemental material has been provided by the authors to give readers additional information about their work.

© 2025 Warner MA et al. *JAMA Network Open*

eTable 1. Additional patient and hospitalization characteristics

	Standard of Care (N=51)	Intervention (N=49)	Total (N=100)
Surgery type, n=65			
Cardiac	21 (67.7%)	25 (73.5%)	46 (70.8%)
General surgery/abdominal	5 (16.1%)	3 (8.8%)	8 (12.3%)
Vascular	0 (0.0%)	3 (8.8%)	3 (4.6%)
Orthopedic/spine	1 (3.2%)	2 (5.9%)	3 (4.6%)
Trauma (non-ortho)	1 (3.2%)	1 (2.9%)	2 (3.1%)
Thoracic	1 (3.2%)	0 (0.0%)	1 (1.5%)
Other	2 (6.5%)	0 (0.0%)	2 (3.1%)
EBL (among post-operative, cc), n=65	808 (225, 1300)	828 (581, 1179)	808 (400, 1250)
Non-operative admission diagnosis type, n=35			
Cardiovascular	10 (50.0%)	6 (40.0%)	16 (45.7%)
Gastrointestinal	0 (0.0%)	6 (40.0%)	6 (17.1%)
Respiratory (including focal pneumonia)	5 (25.0%)	0 (0.0%)	5 (14.3%)
Genitourinary	1 (5.0%)	0 (0.0%)	1 (2.9%)
Infectious disease	0 (0.0%)	1 (6.7%)	1 (2.9%)
Other	4 (20.0%)	2 (13.3%)	6 (17.1%)
Pre-enrollment hospital length of stay (days)	3 (2, 4)	2 (1, 4)	3 (2, 4)
Baseline laboratory values			
Hemoglobin (g/dL)	8.8 (8.4, 9.4)	8.9 (8.3, 9.2)	8.9 (8.4, 9.4)
Ferritin (mcg/L), n=99	238 (136, 437)	202 (126, 407)	223 (129, 430)
Transferrin (%), n=99	12.0 (8.0, 19.8)	12.0 (9.0, 17.0)	12.0 (8.0, 18.5)
Mean corpuscle volume (fL)	91.4 (87.8, 93.6)	91.2 (89.4, 94.9)	91.2 (89.1, 94.4)
Red cell distribution width (%)	14.3 (13.3, 15.5)	14.4 (13.6, 15.1)	14.4 (13.3, 15.4)
Medications			
Aspirin (any dose)	38 (74.5%)	30 (61.2%)	68 (68.0%)
Plavix (clopidogrel)	10 (19.6%)	11 (22.4%)	21 (21.0%)
Heparin infusion	25 (49.0%)	33 (67.3%)	58 (58.0%)
Heparin Sub-Q	29 (56.9%)	25 (51.0%)	54 (54.0%)
Coumadin (warfarin)	10 (19.6%)	4 (8.2%)	14 (14.0%)
Lovenox (enoxaparin)	5 (9.8%)	4 (8.2%)	9 (9.0%)
Chronic comorbidities			
Hypertension	38 (74.5%)	32 (65.3%)	70 (70.0%)
Coronary artery disease	23 (45.1%)	23 (46.9%)	46 (46.0%)
Upper GI disease	18 (35.3%)	15 (30.6%)	33 (33.0%)
Diabetes mellitus	20 (39.2%)	11 (22.4%)	31 (31.0%)
Depression	15 (29.4%)	16 (32.7%)	31 (31.0%)

© 2025 Warner MA et al. *JAMA Network Open*

	Standard of Care (N=51)	Intervention (N=49)	Total (N=100)
Chronic kidney disease	14 (27.5%)	11 (22.4%)	25 (25.0%)
Congestive heart failure	13 (25.5%)	6 (12.2%)	19 (19.0%)
Anxiety	9 (17.6%)	9 (18.4%)	18 (18.0%)
Chronic obstructive pulmonary disease	5 (9.8%)	7 (14.3%)	12 (12.0%)
History of stroke	2 (3.9%)	7 (14.3%)	9 (9.0%)
Peripheral vascular disease	5 (9.8%)	3 (6.1%)	8 (8.0%)
Cancer, solid organ	4 (7.8%)	4 (8.2%)	8 (8.0%)
History of GI bleeding	1 (2.0%)	6 (12.2%)	7 (7.0%)
Chronic liver disease	2 (3.9%)	4 (8.2%)	6 (6.0%)
Alcohol abuse	2 (3.9%)	3 (6.1%)	5 (5.0%)
Post-traumatic stress disorder	4 (7.8%)	0 (0.0%)	4 (4.0%)
Venous thromboembolic disease	2 (3.9%)	2 (4.1%)	4 (4.0%)
Hematological malignancy, n=99	0 (0.0%)	1 (2.1%)	1 (1.0%)
Treatment interventions at time of consent			
Mechanical ventilation	2 (3.9%)	3 (6.1%)	5 (5.0%)
Non-invasive ventilation	3 (5.9%)	4 (8.2%)	7 (7.0%)
Renal replacement therapy	1 (2.0%)	1 (2.0%)	2 (2.0%)
Vasopressor infusions	14 (27.5%)	13 (26.5%)	27 (27.0%)
Inotropes	1 (2.0%)	1 (2.0%)	2 (2.0%)
Mechanical ventilation during hospitalization	46 (90.2%)	43 (87.8%)	89 (89.0%)
Post-enrollment RBC transfusion	20 (39.0%)	24 (49.0%)	44 (44.0%)
Post-enrollment ICU length of stay (days)	1 (0, 2)	1 (0, 2)	1 (0, 2)
Post-enrollment hospital length of stay (days)	6 (4, 7)	5 (3, 8)	5 (4, 8)

* Values are frequency (percentage) for categorical variables and median (25th, 75th percentile) for continuous variables.

© 2025 Warner MA et al. *JAMA Network Open*

eTable 2. Demographic, clinical, and laboratory features for those with and without 1- month post-hospitalization hemoglobin assessments

	No Hb at 1 month (N=12)	Hb measured at 1 month (N=88)	Absolute Std. diff
Age (years)	67 (52, 76)	68 (62, 71)	0.174
Female sex	5 (41.7%)	38 (43.2%)	0.031
Body Mass Index (kg/m^2)	28.6 (26.2, 30.3)	31.1 (27.2, 34.5)	0.455
Ethnicity			0.453
Hispanic or Latino	0 (0.0%)	1 (1.1%)	
Not Hispanic or Latino	11 (91.7%)	87 (98.9%)	
Unknown	1 (8.3%)	0 (0.0%)	
Prior hospital length of stay (days)	1.5 (1, 4)	2 (1, 4)	0.337
APACHE II score	14 (11, 19)	15 (12, 19)	0.075
SOFA score	4 (2, 5)	4 (2, 6)	0.219
ICU admission type, surgical	8 (66.7%)	68 (77.3%)	0.238
ICU admitting diagnosis, post-operative	7 (58.3%)	58 (65.9%)	0.157
EBL >= 500 mL prior to enrollment	6 (50.0%)	54 (61.4%)	0.230
RBC transfusion prior to enrollment	4 (33.3%)	42 (47.7%)	0.296
RBC units prior to enrollment, n=46	2 (2, 3)	2 (1, 3)	0.199
Hemoglobin (g/dL)	8.8 (8.4, 9.0)	8.9 (8.4, 9.4)	0.193
Ferritin (mcg/L), n=99	194 (59, 440)	229 (135, 429)	0.035
Transferrin saturation (%), n=99	12.0 (8.5, 14.0)	12.0 (8.0, 19.0)	0.196
Platelets (x10^9/L)	148 (111, 224)	149 (118, 202)	0.022
White blood count (x10^9/L)	10.6 (8.9, 12.5)	12.0 (8.6, 15.9)	0.201
Creatinine (mg/dL)	1.0 (0.9, 1.2)	1.0 (0.8, 1.4)	0.329
Pre-enrollment hospital length of stay (days)	2 (1, 4)	2 (1, 4)	0.337
Mechanical ventilation during hospitalization	8 (66.7%)	81 (92.0%)	0.660
Post-enrollment ICU length of stay (days)	1 (0, 2)	1 (0, 2)	0.314
Post-enrollment hospital length of stay (days)	6 (3, 11)	5 (4, 8)	0.388

* Values are frequency (percentage) for categorical variables and median (25th, 75th percentile) for continuous variables.
BMI – body mass index; APACHE – Acute Physiology and Chronic Health Evaluation; SOFA – sequential organ failure assessment; ICU – intensive care unit; EBL – estimated blood loss; RBC – red blood cell

© 2025 Warner MA et al. *JAMA Network Open*

eTable 3. Estimated treatment effect for hemoglobin using worst-case imputation (no change from baseline) approach*

Time point	Treatment effect (g/dL) Estimate (95% CI)
ICU discharge	0.01 (-0.34, 0.40)
Hospital discharge	0.22 (-0.15, 0.59)
1-month	0.36 (-0.23, 1.00)
3-months	0.56 (-0.26, 1.39)

Estimates are for increase in hemoglobin associated with treatment at the given time-point. Confidence intervals are estimated from 1000 bootstrap samples.

eTable 4. Adverse events

	Standard Care (N=8 among 6 participants)	Intervention (N=5 among 5 participants)
Bloodstream infection	2 (2)	0
Myocardial infarction	2 (2)	1
Non-hemorrhagic stroke	0	2
Venous thromboembolic disease	4 (4)	2
Severity of adverse event		
Mild	0	2
Moderate	5 (5)	3
Severe	3 (2)	0
Serious adverse event	3 (2)	1
Adverse event related to study drug		
Unlikely related	1 (1)	1
Unrelated	7 (5)	4
Event relating to any other study procedure		
Unlikely related	1 (1)	1
Unrelated	7 (5)	4
Individual patient outcome, n=11		
Recovered alive without sequelae	3	3
Recovered alive with sequelae	1	2
Still under treatment for event	1	0
Died	1	0

* Data are summarized as total number of events (total number of participants). 1 participant in the control group experienced 3 adverse events (bloodstream infection, MI, and venous thromboembolic disease), 2 severe and 1 moderate, 2 defined as serious (prolonged hospitalization, death), all unrelated to study drug or procedure, over an 18-day period.

eTable 5. Phlebotomy Summaries†

	Standard Care (N=51)	Intervention (N=49)	p-value
Totals During Hospitalization			
Total Unique Specimens	46 (20, 74)	32 (24, 55)	0.352
Total Unique Draws	31 (12, 54)	22 (15, 43)	0.412
Total Volume Drawn (mL)	142 (68, 195)	32 (22, 54)	<.001
Total Waste (mL)	10 (0, 32)	0 (0, 0)	<.001
Total Specimen Volume Drawn (mL)	117 (61, 168)	32 (22, 54)	<.001
Total Redraws	0 (0, 2)	0 (0, 2)	0.934
Totals over first 7 days			
Total Unique Specimens	41 (20, 59)	31 (23, 49)	0.406
Total Unique Draws	29 (12, 44)	20 (14, 39)	0.454
Total Volume Drawn (mL)	120 (66, 169)	29 (21, 52)	<.001
Total Waste (mL)	10 (0, 20)	0 (0, 0)	<.001
Total Specimen Volume Drawn (mL)	101 (61, 147)	29 (21, 52)	<.001
Total Redraws	0 (0, 2)	0 (0, 2)	0.695
Day 1*, n = 99			
Total Unique Specimens	6 (4, 8)	6 (5, 8)	0.362
Total Unique Draws	4 (3, 6)	5 (4, 6)	0.171
Total Volume Drawn (mL)	19 (13, 28)	7 (7, 10)	<.001
Total Waste (mL)	0 (0, 0)	0 (0, 0)	0.001
Total Specimen Volume Drawn (mL)	17 (13, 24)	7 (7, 10)	<.001
Day 2			
Total Unique Specimens	8 (6, 12)	9 (5, 13)	0.691
Total Unique Draws	6 (4, 9)	7 (3, 8)	0.811
Total Volume Drawn (mL)	26 (14, 35)	4 (3, 9)	<.001
Total Waste (mL)	0 (0, 8)	0 (0, 0)	<.001
Total Specimen Volume Drawn (mL)	20 (13, 31)	4 (3, 9)	<.001
Day 3, n=97			
Total Unique Specimens	6 (5, 10)	6 (4, 8)	0.185
Total Unique Draws	5 (2, 8)	4 (2, 6)	0.138
Total Volume Drawn (mL)	22 (12, 30)	5 (3, 9)	<.001
Total Waste (mL),	0 (0, 0)	0 (0, 0)	0.001
Total Specimen Volume Drawn (mL)	17 (11, 27)	5 (3, 9)	<.001
Day 4, n=90			
Total Unique Specimens	7 (4, 10)	5 (3, 8)	0.098
Total Unique Draws	5 (2, 7)	3 (1, 6)	0.072
Total Volume Drawn (mL)	19 (11, 30)	4 (3, 9)	<.001

© 2025 Warner MA et al. *JAMA Network Open*

Total Waste (mL)	0 (0, 0)	0 (0, 0)	0.016
Total Specimen Volume Drawn (mL)	18 (11, 25)	4 (3, 9)	<.001
Day 5, n=75			
Total Unique Specimens	6 (4, 8)	4 (2, 9)	0.245
Total Unique Draws	4 (2, 7)	2 (1, 6)	0.156
Total Volume Drawn (mL)	13 (9, 20)	4 (2, 8)	<.001
Total Waste (mL)	0 (0, 0)	0 (0, 0)	0.023
Total Specimen Volume Drawn (mL)	12 (9, 20)	4 (2, 8)	<.001
Day 6, n=62			
Total Unique Specimens	5 (3, 7)	5 (3, 7)	0.892
Total Unique Draws	4 (2, 6)	3 (2, 6)	0.812
Total Volume Drawn (mL)	14 (9, 19)	4 (2, 8)	<.001
Total Waste (mL)	0 (0, 0)	0 (0, 0)	0.015
Total Specimen Volume Drawn (mL)	11 (9, 16)	4 (2, 8)	<.001
Day 7, n=51			
Total Unique Specimens	6 (3, 7)	4 (2, 9)	0.493
Total Unique Draws	3 (1, 5)	2 (1, 5)	0.616
Total Volume Drawn (mL)	12 (8, 22)	3 (2, 6)	<.001
Total Waste (mL)	0 (0, 0)	0 (0, 0)	0.085
Total Specimen Volume Drawn (mL)	12 (8, 20)	3 (2, 6)	<.001

†Data are summarized as median (25th, 75th percentile)

*Day 1 is defined as time of admission into hospital until midnight.

© 2025 Warner MA et al. *JAMA Network Open*

eTable 6. EQ-5D-3L Domain Scores

	Standard Care (N=51)	Intervention (N=49)	p value
**Mobility (hospital discharge), n=98**			0.167
I have no problems in walking about.	6 (12.0%)	12 (25.0%)	
I have some problems in walking about.	43 (86.0%)	36 (75.0%)	
I am confined to bed.	1 (2.0%)	0 (0.0%)	
**Mobility (at 1 month), n=87**			0.310
I have no problems in walking about.	23 (51.1%)	26 (61.9%)	
I have some problems in walking about.	22 (48.9%)	16 (38.1%)	
I am confined to bed.	0 (0.0%)	0 (0.0%)	
**Mobility (at 3 months), n=82**			1.000
I have no problems in walking about.	33 (80.5%)	33 (80.5%)	
I have some problems in walking about.	8 (19.5%)	8 (19.5%)	
I am confined to bed.	0 (0.0%)	0 (0.0%)	
**Self-care (hospital discharge), n=98**			0.262
I have no problems with self-care.	13 (26.0%)	14 (29.2%)	
I have some problems washing or dressing myself.	32 (64.0%)	33 (68.8%)	
I am unable to wash or dress myself.	5 (10.0%)	1 (2.1%)	
**Self-care (at 1 month), n=87**			0.340
I have no problems with self-care.	42 (93.3%)	41 (97.6%)	
I have some problems washing or dressing myself.	3 (6.7%)	1 (2.4%)	
I am unable to wash or dress myself.	0 (0.0%)	0 (0.0%)	
**Self-care (at 3 months), n=82**			0.556
I have no problems with self-care.	40 (97.6%)	39 (95.1%)	
I have some problems washing or dressing myself.	1 (2.4%)	2 (4.9%)	
I am unable to wash or dress myself.	0 (0.0%)	0 (0.0%)	
**Usual Activities (hospital discharge), n=98**			0.033
I have no problems with performing my usual activities.	3 (6.0%)	8 (16.7%)	
I have some problems with performing my usual activities.	36 (72.0%)	37 (77.1%)	
I am unable to perform my usual activities.	11 (22.0%)	3 (6.2%)	
**Usual activities (at 1 month), n=87**			0.578
I have no problems with performing my usual activities.	16 (35.6%)	17 (40.5%)	
I have some problems with performing my usual activities.	28 (62.2%)	25 (59.5%)	
I am unable to perform my usual activities.	1 (2.2%)	0 (0.0%)	
**Usual activities (at 3 months), n=82**			0.607
I have no problems with performing my usual activities.	30 (73.2%)	32 (78.0%)	

© 2025 Warner MA et al. *JAMA Network Open*

	Standard Care (N=51)	Intervention (N=49)	p value
I have some problems with performing my usual activities.	11 (26.8%)	9 (22.0%)	
I am unable to perform my usual activities.	0 (0.0%)	0 (0.0%)	
**Pain and discomfort (hospital discharge), n=98**			0.981
I have no pain or discomfort.	8 (16.0%)	7 (14.6%)	
I have moderate pain or discomfort.	40 (80.0%)	39 (81.2%)	
I have extreme pain or discomfort.	2 (4.0%)	2 (4.2%)	
**Pain and discomfort (at 1 month), n=87**			0.566
I have no pain or discomfort.	22 (48.9%)	19 (45.2%)	
I have moderate pain or discomfort.	22 (48.9%)	23 (54.8%)	
I have extreme pain or discomfort.	1 (2.2%)	0 (0.0%)	
**Pain and discomfort (at 3 months), n=82**			0.332
I have no pain or discomfort.	31 (75.6%)	27 (65.9%)	
I have moderate pain or discomfort.	10 (24.4%)	14 (34.1%)	
I have extreme pain or discomfort.	0 (0.0%)	0 (0.0%)	
**Anxiety and depression (hospital discharge), n=98**			0.511
I am not anxious or depressed.	23 (46.0%)	26 (54.2%)	
I am moderately anxious or depressed.	24 (48.0%)	21 (43.8%)	
I am extremely anxious or depressed.	3 (6.0%)	1 (2.1%)	
**Anxiety and depression (at 1 month), n=87**			0.188
I am not anxious or depressed.	30 (66.7%)	34 (81.0%)	
I am moderately anxious or depressed.	13 (28.9%)	8 (19.0%)	
I am extremely anxious or depressed.	2 (4.4%)	0 (0.0%)	
**Anxiety and depression (at 3 months), n=82**			0.577
I am not anxious or depressed.	32 (78.0%)	34 (82.9%)	
I am moderately anxious or depressed.	9 (22.0%)	7 (17.1%)	
I am extremely anxious or depressed.	0 (0.0%)	0 (0.0%)	

© 2025 Warner MA et al. *JAMA Network Open*

eTable 7. Interaction analyses

	1-month Hb treatment effect	
**Moderator**	Estimate (95% CI)	Interaction p-value
Sex		0.859
Females	0.79 (-0.09, 1.67)	
Males	0.69 (-0.07, 1.45)	
ICU admission type		0.406
Surgical	0.86 (0.21, 1.51)	
Non-Surgical	0.25 (-1.03, 1.53)	
Age (years)		0.516
60	0.86 (0.17, 1.55)	
65	0.75 (0.17, 1.33)	
70	0.64 (-.01, 1.28)	

* Results are from linear models adjusted for age, sex, ICU admission type, and baseline hemoglobin. Estimates are for the increase in 1 month hemoglobin (g/dL) associated with treatment. P-values for interaction effects are provided for each characteristic.

© 2025 Warner MA et al. *JAMA Network Open*

eFigure 1. Hemoglobin values over time for individual participants and study groups

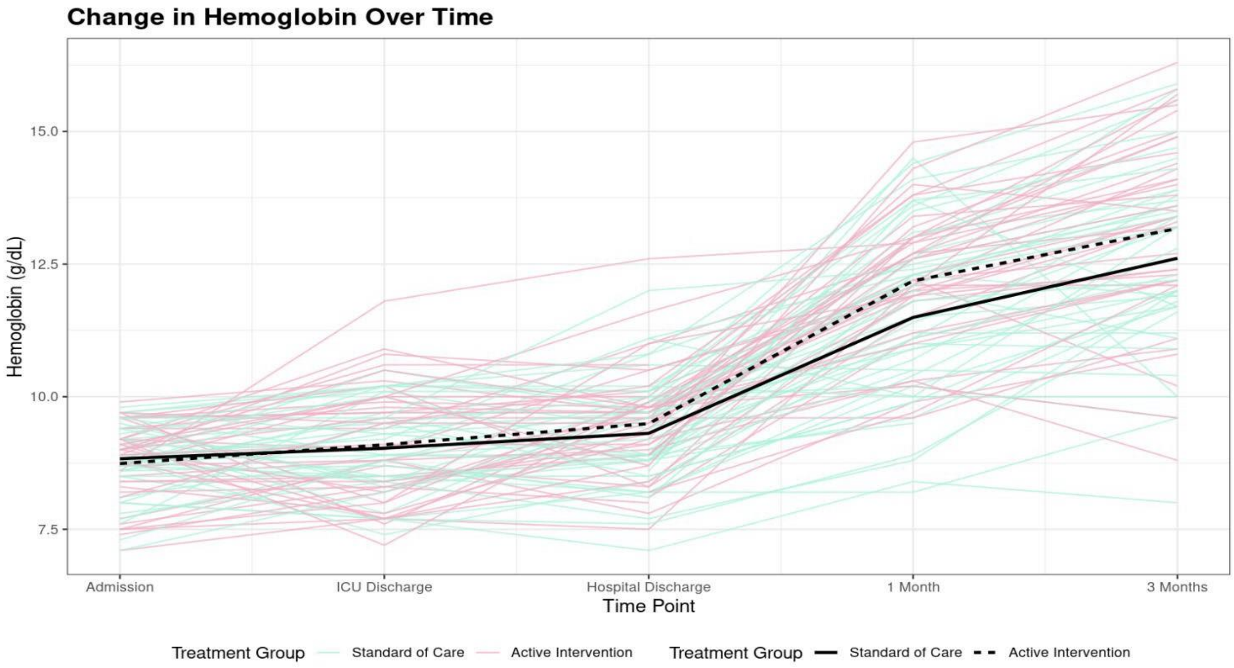

Individual participant hemoglobin trajectories shown in green (standard care) and red (intervention). Mean values displayed in black (solid = standard care; dotted = intervention).

© 2025 Warner MA et al. *JAMA Network Open*

eFigure 2. 6-Minute Walk Distances at 1- and 3-Months

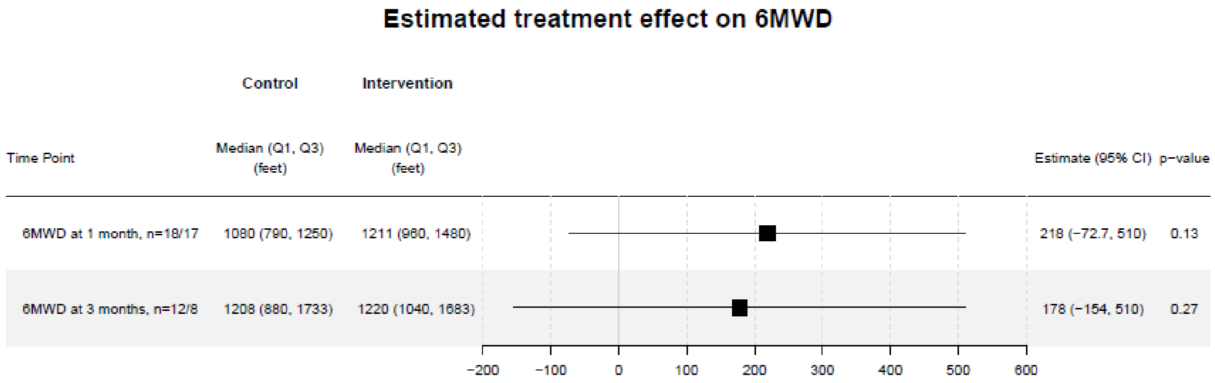

Estimates with 95% confidence intervals represent the increase in mean 6-minute walk distance with intervention.

Analyses are adjusted for baseline KATZ-ADLs.
