# Supplementary material for: Practical Anemia Bundle and Hemoglobin Recovery in Critical Illness: A Randomized Clinical Trial
Source: JAMA Netw Open. 2025 Mar 28;8(3):e252353. doi: 10.1001/jamanetworkopen.2025.2353 (PMC11953759; doi:10.1001/jamanetworkopen.2025.2353)
Supplement: Supplement 3. — Data Sharing Statement [file jamanetwopen-e252353-s003.pdf]

Data Sharing Statement

Warner. Practical Anemia Bundle and Hemoglobin Recovery in Critical Illness. *JAMA Netw Open*. Published March 28, 2025. doi:10.1001/jamanetworkopen.2025.2353

Data
Additional Information: ClinicalTrials.gov. NCT05167734.
https://clinicaltrials.gov/study/NCT05167734
Data available: Yes
Data types: Deidentified participant data, Data dictionary
How to access data: In accordance with National Institutes of Health policy, data will be shared through the National Heart Lung and Blood Institute Data Repository.
When available: With publication

Supporting Documents
Document types: Statistical/analytic code
How to access documents: In accordance with National Institutes of Health policy, data will be shared through the National Heart Lung and Blood Institute Data Repository.
When available: With publication

Additional Information
Who can access the data: Researchers will be required to complete data sharing and confidentiality agreements as established by the sharing repository prior to the release of individual participant data. This must include commitments to not re-identify research participants, to appropriately secure data with strict access rules, to destroy data when analyses are complete, and meet any special requirements as stipulated by the health system involved in the study, and to meet appropriate HIPAA requirements as applicable.
Types of analyses: Any purposes
Mechanisms of data availability: After approval of proposal with data access agreement
